# Synthesis, Larvicidal Activities and Antifungal Activities of Novel Chlorantraniliprole Derivatives and Their Target in the Ryanodine Receptor

**DOI:** 10.3390/molecules20033854

**Published:** 2015-03-02

**Authors:** Qichao Chen, Lixia Xiong, Min Luo, Jin Wang, Changyan Hu, Xiao Zhang, Shujing Yu, Yonghong Li, Dequn Sun

**Affiliations:** 1 Marine College, Shandong University at Weihai, No. 180, Wenhua West Road, Weihai 264209, China; E-Mails: qichao_chen@126.com (Q.C.); Min.Luo@rega.kuleuven.be (M.L.); wangjinsd@yeah.net (J.W.); sdu285@163.com (C.H.); 2 State Key Laboratory of Elemento-Organic Chemistry, Institute of Elemento-Organic Chemistry, Nankai University, Tianjin 300071, China; E-Mails: xionglixia@nankai.edu.cn (L.X.); xiaojun060603@nankai.edu.cn (X.Z.); yushujing@nankai.edu.cn (S.Y.)

**Keywords:** anthranilic diamides, chlorantraniliprole, ryanodine receptor, insecticidal activities, antifungal activities, molecular docking

## Abstract

In order to identify novel chlorantraniliprole derivatives as potential insecticides or fungicides, 25 analogues of chlorantraniliprole were synthesized. The insecticidal activities against oriental armyworm and the antifungal activities against five typical fungi of these derivatives were tested. Compounds **2u**, **2x** and **2y** exhibited good activities against oriental armyworm, especially compounds **2u** and **2x** which showed higher larvicidal activities than indoxacarb. Moreover, all of the tested compounds exhibited activities against five typical fungi. The Ki values of all synthesized compounds were calculated using AutoDock4. The relationship between the Ki values and the results of insecticidal activities against oriental armyworm further indicated that the membrane-spanning domain protein of the ryanodine receptor might contain chlorantraniliprole binding sites.

## 1. Introduction

**Figure 1:**
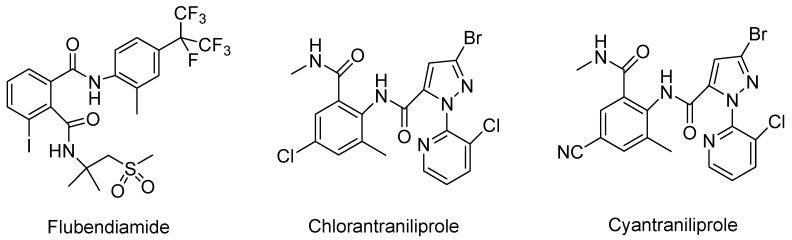
Insecticides acting on the insect ryanodine receptor [8]. Copyright © 2014 Elsevier Ltd.

Lepidopteran pests such as the oriental armyworm (*Mythimna separata*) have become difficult to control because of their emerging resistance to various types of traditional insecticides [1,2]. In order to control the oriental armyworm effectively, there were currently two new types of diamide insecticides with exceptional insecticidal activities on a range of lepidopteran pests, the phthalic diamides, such as flubendiamide [3,4] and the anthranilic diamides, such as chlorantraniliprole [5] and cyantraniliprole (Figure 1) [6].

**Figure 2:**
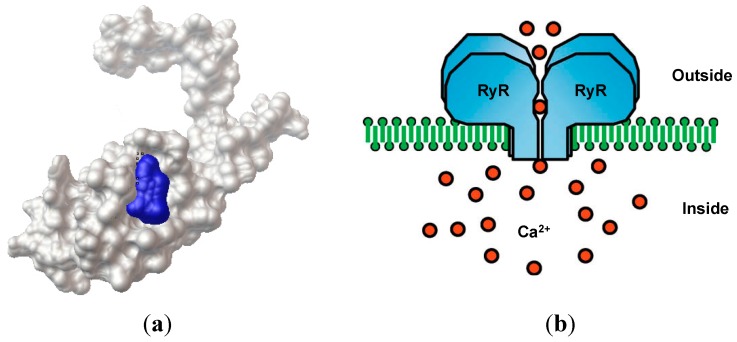
(**a**) Docking orientation of proposed ryanodine receptor(white color) and the chlorantraniliprole (blue color); (**b**) ryanodine receptor (RyR) and its Ca^2+^ release channels [8] Copyright © 2014 Elsevier Ltd.

Chlorantraniliprole, discovered by DuPont, has excellent control of lepidopteran pests, low mammalian toxicity and a favorable environmental profile. The widespread use of chlorantraniliprole on lepidopteran pests in the future may result in pest resistance and residues in the field [7]. Thus, designing novel chlorantraniliprole derivatives as new insecticides has attracted considerable research attention. However, a reasonable design strategy could not be proposed because of the lack of a clear target for chlorantraniliprol. In our previous work [8], we proposed the ryanodine receptor [9] as a possible binding target for chlorantraniliprole and its derivatives using an AutoDock4 analysis, and we initially confirmed that the membrane-spanning domain protein of the ryanodine receptor might have specific binding site(s) for chlorantraniliprole derivatives. Figure 2a shows the relative position relationship between the proposed ryanodine receptor (in white color) and chlorantraniliprole (in blue color). Diamide insecticides activate insect ryanodine (Ry)-sensitive intracellular Ca^2+^ channels by affecting calcium release [10], as seen in Figure 2b. We initially identified the target by comparing the relationship between the insecticidal activities against diamondback moth and the molecular docking results.

Both diamondback moth and oriental armyworm are lepidopteran pests [11]. Therefore, in this study, oriental armyworm was selected for the first time for a bioactivity study, which examines the relationship with the Ki values of molecular docking. On the other hand, it was reported [12] that chlorantraniliprole derivatives might also show antifungal effects. Therefore, in this work the *in vitro* antifungal activity of some chlorantraniliprole derivatives was tested too. The chlorantraniliprole derivatives obtained herein were also used as new probes to bind with the proposed acceptor to further confirm that the membrane-spanning domain protein of ryanodine receptor was the special target binding with chlorantraniliprole and its derivatives.

**Figure 3:**
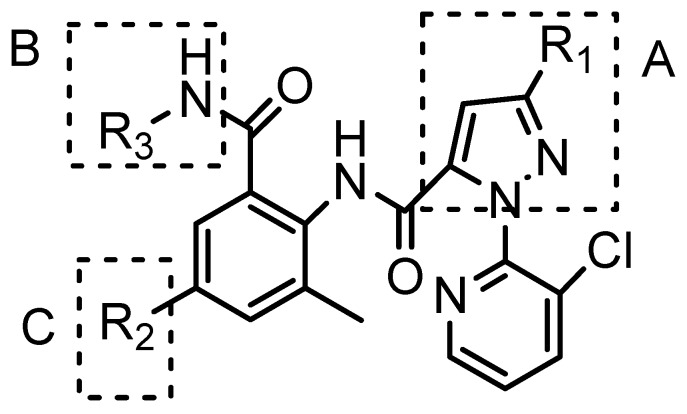
Modifications of chlorantraniliprole derivatives [8]. Copyright © 2014 Elsevier Ltd.

In the previous work [8,13,14,15,16], most modifications of chlorantraniliprole were related to three parts: The pyrazole moiety (part A, Figure 3), amide moiety (part B, Figure 3), and anthraniloyl moiety (part C, Figure 3). Herein, new chlorantraniliprole derivatives were synthesized by modifying the amide-moiety.

## 2. Results and Discussion

### 2.1. Chemistry

**Scheme 1:**
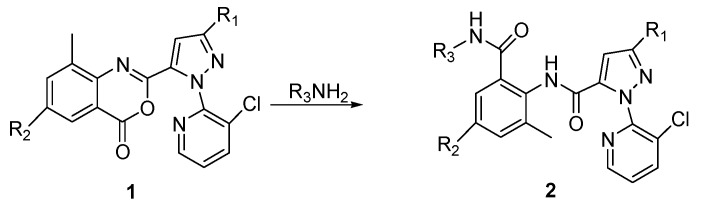
General synthetic route to the title compounds **2a**–**y**.

Compound **1** was obtained by literature methods (Scheme 1) [7,17,18,19]. The nineteen compounds **2b**–**t** were synthesized according to our previous work [8]. Among the new compounds **2a**, **2u**–**y**, compound **2u** was conveniently obtained by treatment of compound **1** with methyl 2-aminoacetate without any other catalyst or base at room temperature. Due to the insolubility of glycine in the reaction solvents (tetrahydrofuran or dichloromethane), a mixture of pyridine and water was selected as solvent in the preparation of compound **2v**. In the case of compounds **2w** and **2x**, a strong base (NaH) was needed, while triethylamine was employed for compound **2w**.

**Table:** 

Compd. No.	R_1_	R_2_	R_3_	Compd. No.	R_1_	R_2_	R_3_
**2a**	Br	Cl	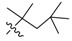	**2n**	Br	Cl	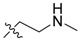
**2b**	Br	Cl	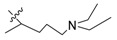	**2o**	Br	CN	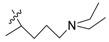
**2c**	Br	Cl	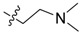	**2p**	Br	CN	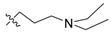
**2d**	Br	Cl	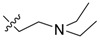	**2q**	Br	CN	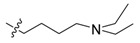
**2e**	Br	Cl	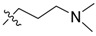	**2r**	Br	CN	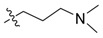
**2f**	Br	Cl	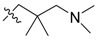	**2s**	Br	CN	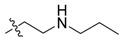
**2g**	Br	Cl	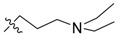	**2t**	Br	CN	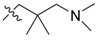
**2h**	Br	Cl	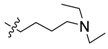	**2u**	Br	Cl	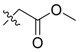
**2i**	Br	Cl	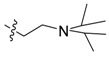	**2v**	Br	Cl	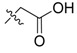
**2j**	Br	Cl	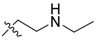	**2w**	Br	Cl	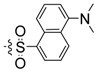
**2k**	Br	Cl	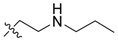	**2x**	Br	Cl	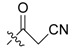
**2l**	Br	Cl	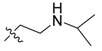	**2y**	Br	Cl	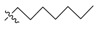
**2m**	Br	Cl	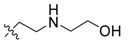				

### 2.2. Larvicidal Activities and Structure-Activity Relationships (SARs)

The results in Table 1 indicate that all of the tested compounds **2a**–**y**, except for four compounds (**2a**, **2q**, **2r** and **2w**) showed the same larvicidal activities (100%) as chlorantraniliprole, indoxacarb and avermectins at the concentrations of 100 and 200 mg/L. These results reveal that most of the chlorantraniliprole derivatives in our study exhibited considerable insecticidal activities against oriental armyworm.

The larvicidal activities of all compounds except for three compounds (**2u**, **2x** and **2y**) were lower than those of chlorantraniliprole and avermectins when the concentration was under 50 mg/L, however, compounds **2u** and **2x** showed obviously better activities (both 100%) than indoxacarb (40%), even when the concentration was lowered to 5 mg/L, while, compound **2y** displayed the same larvicidal activity (40%) as indoxacarb at this concentration. Notably, these two compounds **2u** and **2x** displayed the same activity (100%) as chlorantraniliprole and avermectins at the concentration of 5 mg/L.

**Table 1:** Insecticidal activities of title compounds **2a**–**y**, chlorantraniliprole, indoxacarb and avermectins against oriental armyworm.

Compd. No.	Insecticidal Activities (%) at Different Concentrations					
	Concentrations (mg/L)					
**2a**	60					
**2b**	100	100	40			
**2c**	100	100	100	100	40	
**2d**	100	100	100	100	60	
**2e**	100	100	100	100	60	
**2f**	100	100	100	100	40	
**2g**	100	100	60			
**2h**	100	100	60			
**2i**	100	100	60			
**2j**	100	100	100	100	20	
**2k**	100	100	40			
**2l**	100	100	100	60		
**2m**	100	100	100	100	20	
**2n**	100	100	100	60		
**2o**	100	100	60			
**2p**	100	100	70			
**2q**	100	60				
**2r**	20					
**2s**	100	100	40			
**2t**	100	100	60			
**2u**	100	100	100	100	100	100
**2v**	100	100	40			
**2w**	30					
**2x**	100	100	100	100	100	100
**2y**	100	100	100	100	100	40
**Chlorantraniliprole**	100	100	100	100	100	100
**Indoxacarb**	100	100	100	100	100	40
**Avermectins**	100	100	100	100	100	100

In Table 2, the preliminary structure-activity relationship (SAR) data is summarized. All compounds with cyano groups (R_2_ = CN) instead of a chloride group commonly had low insecticidal activities against oriental armyworm. For example, compounds **2o**, **2p**, **2q**, **2r**, **2s** and **2t** in Table 1 had no insecticidal activities when the concentration was less than 25 mg/L.

Most of the researchers in previous work [20,21,22] preserved the anthranilic amide moiety, which suggested that this structure was an important pharmacophore in those compounds [23]. The number of methylene groups in the amide moiety was also an important element for the insecticidal activity and two methylenes was the most favorable number for high activity. For example, compounds **2c**, **2d**, **2j**, **2l** and **2n** (*n* = 2) had high insecticidal activities (100%), while compounds **2g** and **2h** (*n* = 3 or 4) showed lower activities (60%). Obviously, when R_4_ and R_5_ had the same substituent groups (Table 2) as in compounds **2d**, **2g** and **2h** (R_4_ = R_5_ = ethyl group), the insecticidal activities were dramatically reduced.

**Table 2:** SAR study of typical chlorantraniliprole derivatives. 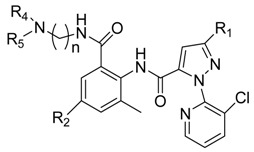

Compd. No.	The Number of Methylenes (n)	R_4_	R_5_	Insecticidal Activities * (%)
**2c**	2	CH_3_	CH_3_	100
**2e**	3	CH_3_	CH_3_	100
**2d**	2	CH_3_CH_2_	CH_3_CH_2_	100
**2g**	3	CH_3_CH_2_	CH_3_CH_2_	60
**2h**	4	CH_3_CH_2_	CH_3_CH_2_	60
**2i**	2	(CH_3_)_2_CH	(CH_3_)_2_CH	60
**2j**	2	CH_3_CH_2_	H	100
**2k**	2	CH_3_CH_2_CH_2_	H	40
**2l**	2	(CH_3_)_2_CH	H	100
**2n**	2	CH_3_	H	100

* The insecticidal activities against oriental armyworm at a concentration of 50 mg/L.

In addition, the substitution of R_4_ or R_5_ at the tail of the amide moiety was a significant factor affecting the insecticidal activities. When either one of R_4_ or R_5_ was substituted by hydrogen, most compounds (**2j**, **2l** and **2n**) displayed relatively high activities (100%), except compound **2k** (40%), while, when both R_4_ and R_5_ were substituted by alkyl groups, most of compounds (**2g**, **2h**, and **2i**) displayed relatively lower activities (60%), except compound **2d** (100%), which indicated that the secondary amine at the tail of the amide moiety was an necessary pharmacophore. In conclusion, the substitution at the terminal positions of the amide moiety and the number of methylene groups might be important factors that influence the insecticidal activities, which could be verified when more chlorantraniliprole derivatives are designed to increase the insecticidal activities against oriental armyworm.

### 2.3. Antifungal Activities

According to the data presented in Table 3, all of the chlorantraniliprole derivatives exhibited certain inhibiory effects against the five tested phytopathogenic fungi: *Fusarium oxysporum* (FO); *Cercospora arachidicola* (CA); *Physalospora piricola* (PP); *Alternaria solani* (AS); and *Fusarium graminearum* (FG).

The activities on FO and CA for all compounds, except **2t** and **2f** (both were 0%) showed varying inhibitory activities (ranging from 4.2% to 41.7%). Regarding the antifungal activities on PP, all tested compounds showed low to good inhibitory activities (from 5.3% to 78.9%) and two compounds (**2h** and **2j**) showed higher activities (78.9%, 63%, respectively) than other compounds. The activities on AS for all compounds except for three (**2k**, **2l** and **2t,** no activity) showed inhibitory activities ranging from 11.1% to 50.0%.

**Table 3:** The antifungal activities against five fungi.

Fungi Compd. No.	Antifungal Activities (%) Against Five Fungi				
	FO	CA	PP	AS	FG
**2b**	11.4	20.8	31.6	38.9	51.9
**2c**	8.7	12.5	10.5	22.2	37.5
**2d**	20.0	20.8	28.1	22.2	29.6
**2e**	14.3	29.2	26.3	11.1	14.8
**2f**	8.6	0.0	22.8	38.9	37.0
**2g**	40.0	29.2	54.4	33.3	44.4
**2h**	25.7	41.7	78.9	11.1	29.6
**2i**	11.4	20.8	33.3	27.8	25.9
**2j**	20.0	29.2	63.2	22.2	40.7
**2k**	11.4	12.5	22.8	0	40.7
**2l**	11.4	29.2	33.3	0	14.8
**2o**	8.7	37.5	5.3	22.2	40.6
**2p**	13.0	31.3	7.9	16.7	31.3
**2q**	13.0	12.5	7.9	16.7	18.8
**2r**	5.7	12.5	28.1	27.8	40.7
**2s**	11.4	20.8	31.6	50.0	44.4
**2t**	0.0	4.2	24.6	0	18.5
**2u**	8.7	25.0	10.5	16.7	46.9
**2y**	8.7	6.3	7.9	11.1	25.0

**FO**: *Fusarium oxysporum*; **CA**: *Cercospora arachidicola*; **PP**: *Physalospora piricola*; **AS**: *Alternaria solani*; **FG**: *Fusarium graminearum*.

The activities of all tested compounds on FG were commonly higher than those on FO and AS. All compounds showed low to average inhibitory activities (from 14.8% to 51.9%) against FG and the compound **2b** showed better activity (51.9%) than the other compounds. 

### 2.4. Molecular Docking Results

The results in Table 4 reveal that ten compounds (**2e**, **2l**, **2m**, **2o**, **2q**, **2r**, **2s**, **2t**, **2w** and **2y**) with unavailable Ki values except for four of them (**2e**, **2l**, **2m** and **2y**) displayed low biological activities (≤60%) against oriental armyworm, while the other 15 compounds (**2a**, **2b**, **2c**, **2d**, **2f**, **2g**, **2h**, **2i**, **2j**, **2k**, **2n**, **2p**, **2u**, **2v** and **2x**) with available Ki value, except one compound (**2a**), generally displayed medium to high biological activities (40%–100%). These results further confirm that the membrane-spanning domain protein (GenBank accession no. JF927788) of the ryanodine receptor selected in our previous work [8] might have specific binding site(s) for chlorantraniliprole and its derivatives, and identification of the possible binding site is worthy of further study from a protein molecular level point of view. However, the docking software program might affect the accuracy of the Ki values and the biological diversity of pests plays an important role in the experimental activity tests. These reasons could explain why compounds **2e**, **2l**, **2m** and **2y** with unavailable Ki values showed high activities (100%) and compound **2a** with available Ki values showed relatively no activity.

**Table 4:** The Ki values and the insecticidal activities against oriental armyworm.

Compd. No.	Ki Values	Insecticidal Activities *	Compd. No.	Ki values ^#^	Insecticidal Activities *
**2h**	3.64	60	**2e**	unavailable	100
**2j**	5.98	100	**2l**	unavailable	100
**2f**	8.25	100	**2m**	unavailable	100
**2b**	14.4	40	**2o**	unavailable	60
**2a**	8.41	0	**2q**	unavailable	0
**2g**	15.16	60	**2r**	unavailable	0
**2d**	17.68	100	**2s**	unavailable	40
**2p**	17.85	70	**2t**	unavailable	60
**2x**	21.07	100	**2w**	unavailable	0
**2i**	28.27	60	**2y**	unavailable	100
**2u**	63.83	100			
**2n**	64.92	100			
**2k**	99.57	40			
**2c**	93.86	100			
**2v**	916.28	40			
**Chlorantraniliprole**	50.12	100			

* Insecticidal activities of tested compounds against oriental armyworm at 50 mg/L; ^#^ Unavailable: The compound couldn’t interact with the selected receptor and the Ki values couldn’t be calculated.

## 3. Experimental Section

### 3.1. Chemistry

#### 3.1.1. General

Melting points (mp) of the products were determined in open capillary tubes and are uncorrected. The products were purified by column chromatography by using silica gel (200–300 mesh). ^1^H-NMR and ^13^C-NMR spectra were recorded on a Varian-400 instrument at room temperature with TMS as an internal standard and CDCl_3_ or DMSO-*d*_6_ as solvents. Mass spectra were recorded with a JEOL MS-D 300 mass spectrometer. The reactions were monitored by analytical thin-layer chromatography TLC with ultraviolet (UV) light and the TLC was carried out on silica gel GF_254_. All reagents were purchased from Acros (Geel, Belgium) or Alfa Aesar (Deisenhofen, Germany). The anhydrous solvents were dried and purified according to standard techniques before use.

#### 3.1.2. Syntheses

The intermediate compound **1** was obtained in six steps by literature methods [7,17,18]. The nineteen compounds **2b**–**t** were synthesized according to our previous methods [8]. The other six new title compounds **2a**, **2u**–**y** were prepared as follows:

*3-Bromo-N-(4-chloro-2-methyl-6-((2,4,4-trimethylpentan-2-yl)carbamoyl)phenyl)-1-(3-chloropyridin-2-yl)-1H-pyrazole-5-carboxamide* (**2a**). 2,4,4-Trimethylpentan-2-amine (28 mg, 0.22 mmol) was added to compound **1** (50 mg, 0.11 mmol) in tetrahydrofuran (THF, 5 mL). Then the mixture was stirred at room temperature and monitored by TLC. The crude product was recrystallized from a dichloromethane/hexane (1:2) to give the product **2a** as a white solid in 55% yield. mp. 202–206 °C ^1^H-NMR (CDCl_3_): δ 11.84 (s, 1H, NHCOC), 8.37 (d, *J* = 4.8 Hz, 1H, 6-H pyridine), 7.78 (d, *J* = 8 Hz, Ph-H), 7.78 (d, *J* = 8 Hz, 1H, 4-H pyridine), 7.31 (dd, *J* = 4.8, 8 Hz, 1H, 5-H pyridine), 7.23 (d, *J* = 1.6 Hz, 1H, Ph-H), 7.01 (s, 1H, pyrazole-H), 2.18 (s, 3H, Ph-CH_3_), 1.56 (s, 2H, CH_2_), 1.30 (s, 6H, (CH_3_)_2_C), 0.89 (s, 9H, (CH_3_)_3_C); ^13^C-NMR (CDCl_3_): δ 172.42, 155.66, 148.91, 146.77, 140.10, 139.07, 136.29, 134.78, 133.14, 130.47, 129.06, 128.09, 127.95, 125.79, 110.77, 55.99, 52.97, 31.23, 31.18, 27.26, 19.44; HRMS (ESI): *m/z* [M+H]^+^ calcd for C_25_H_28_BrCl_2_N_5_O_2_: 602.0701. Found: 602.1055.

*3-Bromo-N-(4-chloro-2-((2-(2-(methoxy)oxoethyl))carbamoyl)-6-methylphenyl)-1-(3-chloropyridin-2-yl)-1H-pyrazole-5-carboxamide* (**2u**). To a solution of methyl glycinate (29 mg, 0.33 mmol) in dichloromethane (DCM, 2 mL), a solution of **1** (148 mg, 0.33 mmol) in DCM (3 mL) was added dropwise at 0 °C. Then the resulting mixture was stirred at room temperature and monitored by TLC. The reaction mixture was evaporated to remove most of the DCM to give the crude product, which was recrystallized from *n*-hexane to give **2u** as a white solid in 40% yield. mp. 142–146 °C; ^1^H-NMR (CDCl_3_): δ 9.84 (s, 1H, CONH-Ar), 8.42 (d, *J* = 7.2 Hz, 1H, pyridyl-H), 7.84 (d, *J* = 8 Hz, 1H, pyridyl-H), 7.36 (dd, *J* = 4.8 Hz, 8 Hz, 1H, pyridyl-H), 7.29 (s, 1H, Ph-H), 7.25 (d, *J* = 5.2 Hz, 1H, Ph-H), 7.05 (s, 1H, pyrazolyl-H), 6.76 (br, s, 1H, NHCO-Ar), 4.12 (d, *J* = 5.6 Hz, 2H, COCH_2_NH), 3.77 (s, 3H, CH_3_O), 2.17 (s, 3H, Ph-CH_3_); ^13^C-NMR (CDCl_3_): δ 169.55, 167.89, 156.34, 156.18, 148.90, 146.75, 138.98, 138.56, 133.37, 132.20, 131.79, 131.19, 128.94, 128.13, 125.67, 124.78, 110.91, 52.62, 41.69, 18.82; HRMS (ESI): *m/z* [M+H]^+^ calcd for C_20_H_17_BrCl_2_N_5_O_4_: 553.0521. Found: 553.0526.

*Methyl2-(2-(2-(3-bromo-1-(3-chloropyridin-2-yl)-1H-pyrazole-5-carboxamido-5-chloro-3-methyl-benzamido)acetic acid* (**2v**). To a solution of glycine (24 mg, 0.33 mmol) in a mixed solvent of pyridine/water (1:1.5, 5 mL), a solution of **1** (0.11 mmol) in pyridine/water (1:1.5, 2 mL) was added dropwise at 0 °C Then the mixture was stirred at room temperature and monitored by TLC. When the reaction mixture was adjusted to pH 2–3 by addition of 1 mol/L hydrochloric acid, a white solid precipitated, which was collected and dried to give **2v** in 52% yield. mp. 212–214 °C; ^1^H-NMR (DMSO-*d*_6_): δ 12.67 (s, 1H, COOH), 10.54 (s, 1H, CONH-Ar), 8.83 (s, 1H, Ar-CONH), 8.49 (d, *J* = 4.4 Hz, 1H, pyridyl-H), 8.17 (d, *J* = 4 Hz, 1H, pyridyl-H), 7.61 (dd, *J* = 4.8 Hz, 8 Hz, 1H, pyridyl-H), 7.53 (s, 1H, pyrazolyl-H), 7.50 (s, 1H, Ph-H), 7.39 (s, 1H, Ph-H), 3.82 (d, *J* = 5.6 Hz, 2H, COCH_2_NH), 2.16 (s, 3H, Ph-CH_3_); ^13^C-NMR (DMSO-*d*_6_): δ 170.55, 165.68, 155.54, 148.34, 146.88, 139.07, 135.57, 131.33, 130.94, 127.74, 126.58, 126.40, 125.63, 110.83, 40.97, 17.60; HRMS (ESI): *m*/*z* [M+H]^+^ calcd for C_19_H_15_BrCl_2_N_5_O_4_: 527.9664. Found: 527.9668.

*3-Bromo-N-(4-chloro-2-((2-(5-(dimethylamino)naphthalen-1-ylsulfonyl))carbamoyl)-6-methylphenyl)-1-(3-chloropyridin-2-yl)-1H-pyrazole-5-carboxamide* (**2w**). To a solution of 5-(dimethylamino)-naphthalene-1-sulfonamide (94 mg, 0.4 mmol) and NaH (14 mg, 0.6 mmol) in THF (5 mL), a solution of **1** (180 mg, 0.4 mmol) in THF (3 mL) was added dropwise at 0 °C. Then the resulting mixture was stirred for 10 min at room temperature. The reaction mixture was washed with 1 mol/L hydrochloric acid to pH 5. The aqueous phase was extracted with DCM (20 mL) and then dried to give the crude residue that was recrystallized from a DCM/hexane (1:1.5) mixture to give the product **2w** as a yellow solid in 77% yield. mp. 142–146 °C; ^1^H-NMR (DMSO-*d*_6_): δ 10.23 (s, 1H, CONH-Ar), 8.53 (dd, *J* = 1.6, 4.8 Hz, 1H, pyridyl-H), 8.17 (dd, *J* = 1.6, 8Hz, 1H, pyridyl-H), 8.32 (s, 1H, Ph-H), 8.30 (s, 1H, Ph-H), 8.10 (dd, *J* = 4.8Hz, 8 Hz, 1H, pyridyl-H), 7.252–7.690 (m, 6H, pyridine-H), 6.95 (s, 1H, pyrazolyl-H), 3.84 (s, 6H, 2NCH_3_), 2.11 (s, 3H, Ph-CH_3_); ^13^C-NMR (DMSO-*d*_6_): δ 163.90, 155.66, 151.34, 148.00, 146.93, 139.20, 139.15, 132.86, 132.67, 131.93, 130.77, 128.89, 128.18, 127.44, 126.58, 126.32, 126.01, 123.24, 118.06, 115.06, 110.52, 109.19, 44.93, 17.37; HRMS (ESI): *m/z* [M+H]^+^ calcd for C_29_H_24_BrCl_2_N_6_O_4_S: 701.0140. Found: 701.0092.

*3-Bromo-N-(4-chloro-2-((2-cyanoacetyl)carbamoyl)-6-methylphenyl)-1-(3-chloropyridin-2-yl)-1H-pyrazole-5-carboxamide* (**2x**). To a solution of 2-cyanoacetamide (11 mg, 0.13 mmol) and NaH (1 mg, 0.17 mmol) in THF (3 mL), a solution of **1** (0.11 mmol) in THF (1 mL) was added dropwise at room temperature. Then the mixture was stirred for 30 min and monitored by TLC. The reaction mixture was evaporated to give the product **2x** as a white solid in 50% yield. mp. 163–166 °C; ^1^H-NMR (DMSO-*d*_6_): δ 10.31 (s, 1H, CONH-Ar), 8.49 (d, *J* = 4.4 Hz, 1H, pyridyl-H), 8.16 (d, *J* = 4 Hz, 1H, pyridyl-H), 7.60 (dd, *J* = 4.8 Hz, 8 Hz, 1H, pyridyl-H), 7.57 (s, 1H, pyrazolyl-H), 7.48 (s, 1H, Ph-H), 7.33 (s, 1H, Ph-H), 3.58(d, *J* = 5.6 Hz, 2H, COCH_2_NH), 2.23 (s, 3H, Ph-CH_3_); ^13^C-NMR (DMSO-*d*_6_): δ 182.92, 169.50, 152.51, 145.12, 143.97,136.17, 135.92, 129.07, 127.88, 124.62, 123.57, 123.40, 122.90, 107.58, 36.50, 14.46; HRMS (ESI): *m/z* [M+H]^+^ calcd for C_20_H_14_BrCl_2_N_6_O_3_: 553.9610. Found: 554.9688.

*3-Bromo-N-(4-chloro-2-((2-heptyl)carbamoyl)-6-methylphenyl)-1-(3-chloropyridin-2-yl)-1H-pyrazole-5-carboxamide* (**2y**). n-Heptylamine (25 mg, 0.22 mmol) was added to a solution of compound **1** (50 mg, 0.11 mmol) in THF (5 mL) at 50 °C. After 5 h, TLC showed the complete consumption of compound **1**. The mixed solution was evaporated to remove the THF. The residue was dissolved in DCM (20 mL). The organic layer was washed with H_2_O, and dried to give a crude product that was then recrystallized from DCM/hexane (1:1.5) to give the product **2y** as a white solid in 58% yield; mp. 162–168 °C; ^1^H-NMR (CDCl_3_): δ 10.12 (s, 1H, NHCOCH_2_), 8.44 (dd, *J* = 4.8 Hz, 1.6 Hz, 1H, 6-H pyridine), 7.83 (dd, *J* = 4.8 Hz, 1.6 Hz, 1H, 4-H pyridine), 7.36 (dd, *J* = 4.8 Hz, 8 Hz, 1H, 5-H pyridine), 7.21 (s, 1H, Ph-H), 7.17 (d, *J* = 6.4 Hz, 1H, NHCO), 7.15 (s, 1H, Ph-H), 6.14 (s, 1H, pyrazole-H), 3.35 (m, *J* = 7.2 Hz, 2H, CH_2_NH), 2.16 (s, 3H, Ph-CH_3_), 1.52 (m, *J* = 7.2 Hz, 2H, CH_2_CH_3_), 1.28 (m, *J* = 14 Hz, 8H, (CH_2_)_4_), 0.89 (t, *J* = 7.2 Hz, 3H, CH_3_); ^13^C-NMR (CDCl_3_): δ 167.84, 156.56, 149.09, 146.79, 138.81, 138.72, 133.22, 132.65, 132.26, 131.27, 129.00, 128.21, 125.63, 124.35, 111.03, 40.34, 31.68, 29.37, 28.91, 26.92, 22.59, 18.70, 14.06; HRMS (ESI): *m/z* [M+H]^+^ calcd for C_24_H_26_BrCl_2_N_5_O_2_: 566.0725. Found: 566.0740.

### 3.2. Biological Tests

#### 3.2.1. Biological Assay Methods

All of the tested compounds had the purity of more than 95%, and all bioassays were performed on representative test organisms reared in the laboratory. Evaluations were based on a percentage scale of 0–100, in which 0 = no activity and 100 = total kill. The standard deviations of the tested biological values were ±5%.

#### 3.2.2. Larvicidal Activities against Oriental Armyworm (*Mythimna separata* Walker)

The larvicidal activities against oriental armyworm were tested by foliar application [7]. The test was repeated at 25 °C ± 1 °C according to statistical requirements. The tested compounds were dissolved in acetone and diluted with water to the required concentrations from 200 to 5 mg/L for bioassay. Individual corn leaves were placed on moistened pieces of filter paper in Petri dishes. The leaves were then dipped in the test solution and allowed to dry. The dishes were infested with 10 fourth-instar oriental armyworm larvae. Percentage mortalities were evaluated 24 h after treatment. Each treatment was replicated for three times. For comparative purposes, chlorantraniliprole, indoxacarb and avermectins were selected as the controls under the same conditions.

#### 3.2.3. Antifungal Bioassay: Inhibitory Effects on Phytopathogenic Fungi

The five phytopathogenic fungi chosen included *Fusarium oxysporum* (FO), *Cercospora arachidicola* (CA), *Physalospora piricola* (PP), *Alternaria solani* (AS), and *Fusarium graminearum* (FG). All the fungi are typical and often occur in the Chinese agro-ecosystem. The antifungal activities of nineteen chlorantraniliprole derivatives (**2b**–**l**, **2o**–**u** and **2x**) were tested in vitro by the poisoned food technique at the concentration of 50 µg/mL by dissolving the compounds in DMSO and sterilized water (containing 1% Tween) and diluting with PDA in a Petri dish [24,25,26].

### 3.3. Molecular Docking Methods

In our study, the membrane-spanning domain protein of the ryanodine receptor was selected from GenBank (accession No. JF927788) [8] as the possible specific receptor, which was proposed to bind with small molecules to give the Ki values using AutoDock4. For the AutoDock4 docking methods readers can refer to our previous work [8]. The Ki values were used to evaluate the binding energy between the small molecules and their possible receptor. The analysis of the relationship between the Ki values and the insecticidal activities against oriental armyworm could thus afford further information about the specific receptor.

## 4. Conclusions

A series of novel chlorantraniliprole derivatives containing different amide groups and anthraniloyl moieties were designed and synthesized. Their insecticidal activities against oriental armyworm and the antifungal activities against five typical fungi were evaluated. The results indicated that all tested compounds except three (**2a**, **2r** and **2w**) exhibited favorable insecticidal activities against oriental armyworm. In particular, compounds **2u** and **2x** showed obviously better activities than indoxacarb even when the concentration was lowered to 5 mg/L. The preliminary structure-activity relationship of the title compounds indicated that compounds with a cyano group (R_2_ = CN) had low insecticidal activities. Two methylene groups and a secondary amine in the amide moiety were necessary factors for increasing the insecticidal activities. Moreover, all of the tested compounds exhibited activities against five typical fungi. The molecular docking results revealed that most of the compounds with available Ki values exhibited moderate to high activities against oriental armyworm. The relationship between the Ki values and the insecticidal activities suggested that the proposed membrane-spanning domain protein (GenBank accession no. JF927788) of the ryanodine receptor has special binding site(s) and might be the receptor of chlorantraniliprole and its analogs. Confirmation of this is a worthy topic for further study.

## Supplementary Materials

Supplementary materials can be accessed at: http://www.mdpi.com/1420-3049/20/03/3854/s1.
