# Supplementary material for: Synthesis, Larvicidal Activities and Antifungal Activities of Novel Chlorantraniliprole Derivatives and Their Target in the Ryanodine Receptor
Source: Molecules. 2015 Mar 2;20(3):3854–67. doi: 10.3390/molecules20033854 (PMC6272368; doi:10.3390/molecules20033854)
Supplement: Supplementary file 1 [file molecules-20-03854-s001.pdf]

Supplementary Materials

1. H-NMR

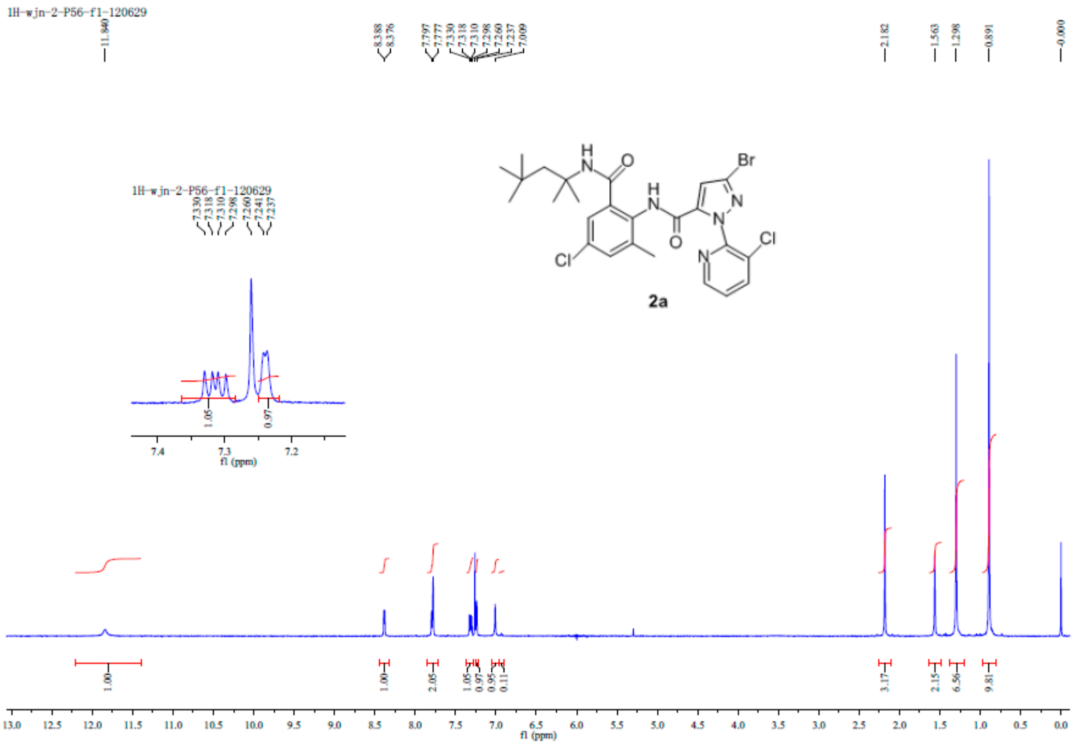

Figure S1. ^{1}H-NMR spectrum of compound **2a**.

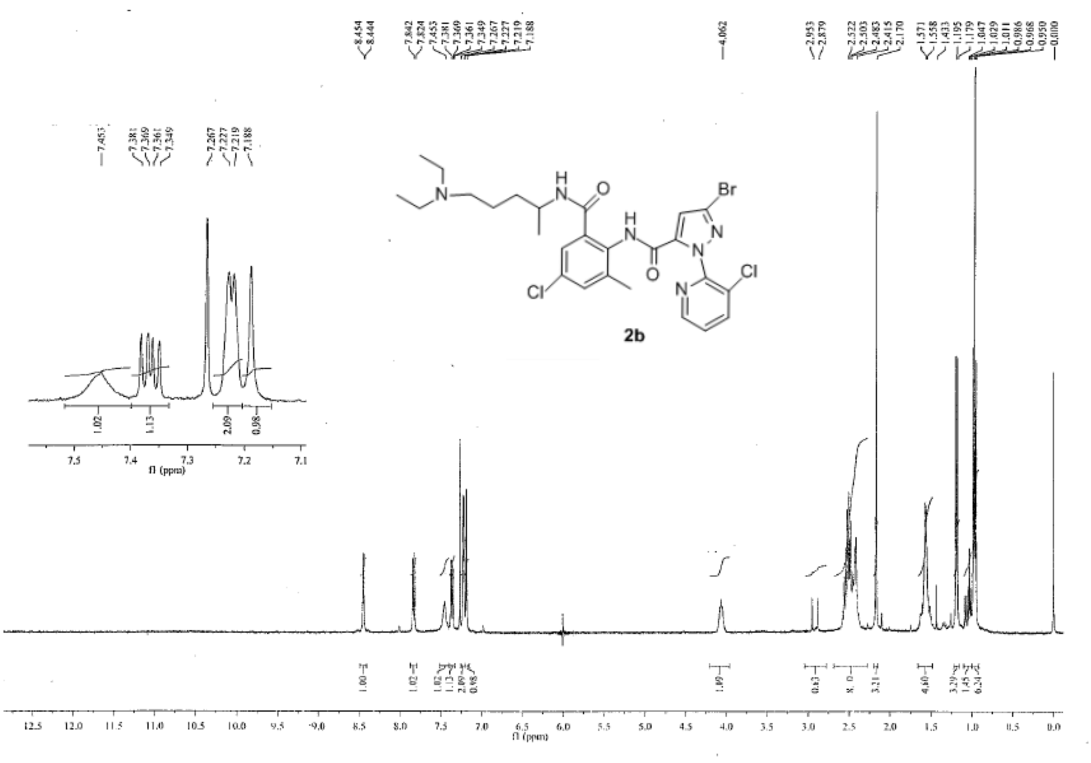

Figure S2. ^{1}H-NMR spectrum of compound **2b**.

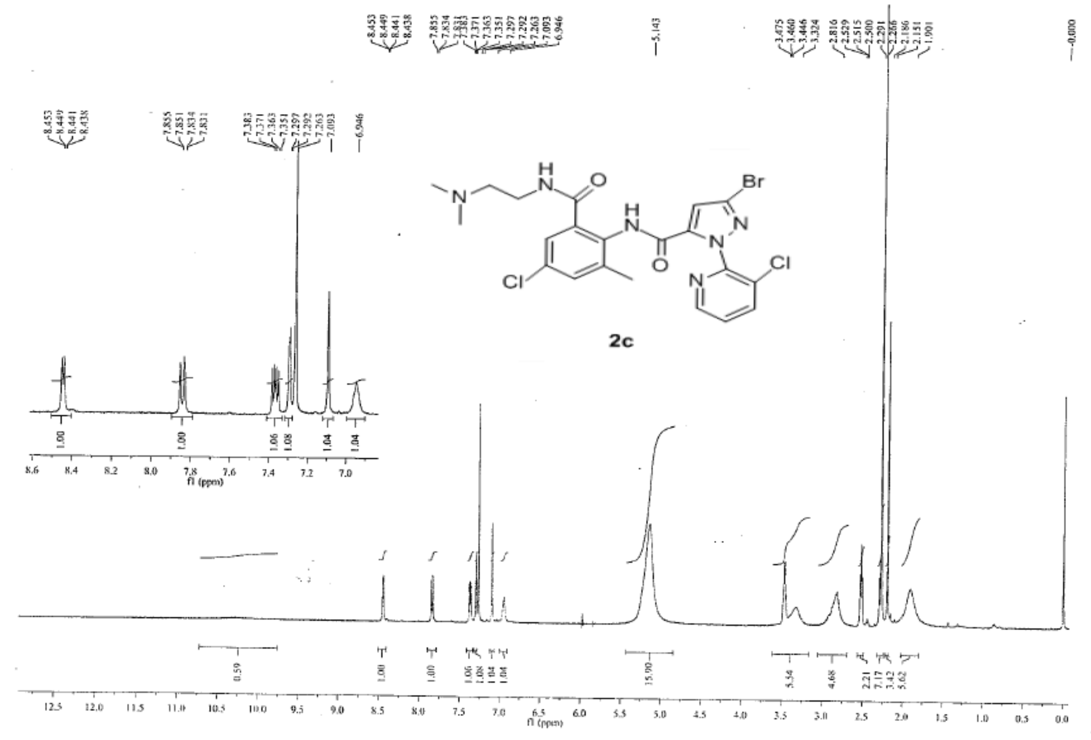

Figure S3. ^{1}H-NMR spectrum of compound **2c**.

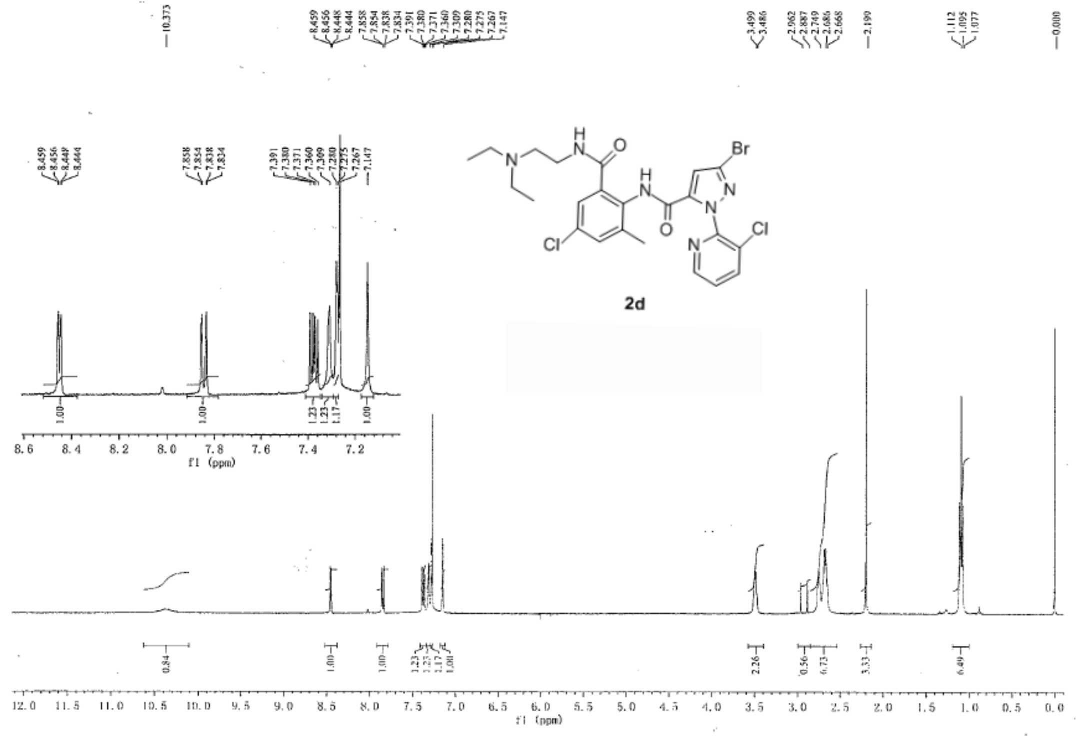

Figure S4. ^{1}H-NMR spectrum of compound **2d**.

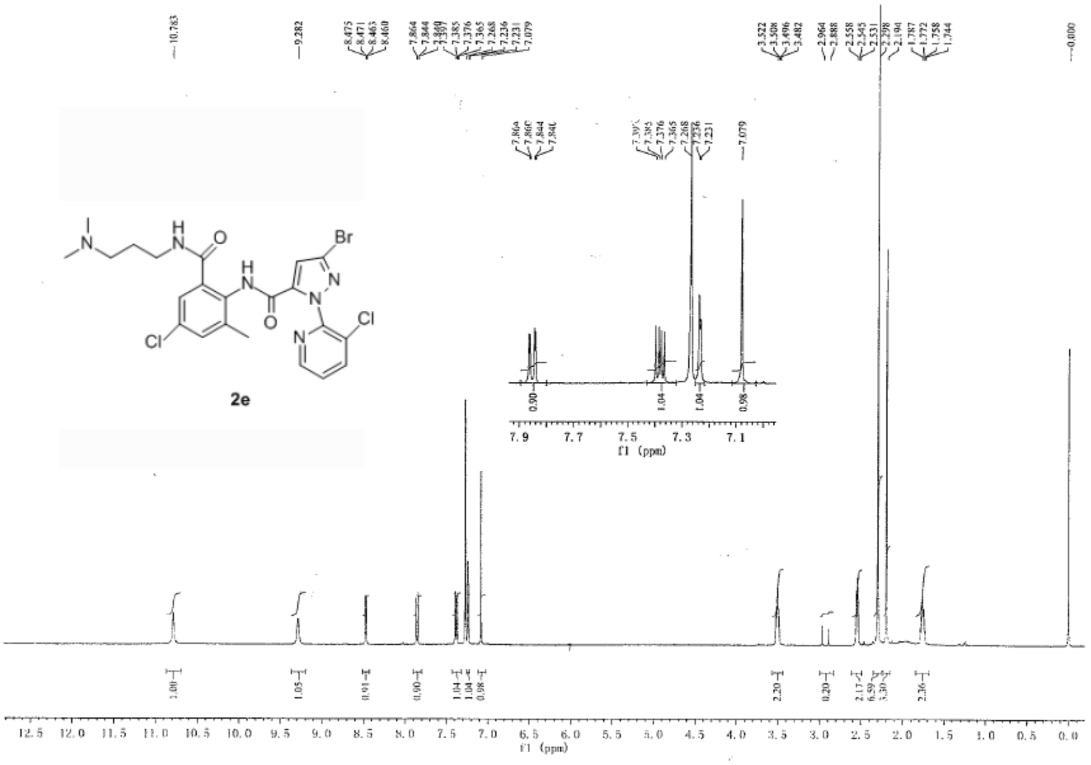

Figure S5. ^{1}H-NMR spectrum of compound **2e**.

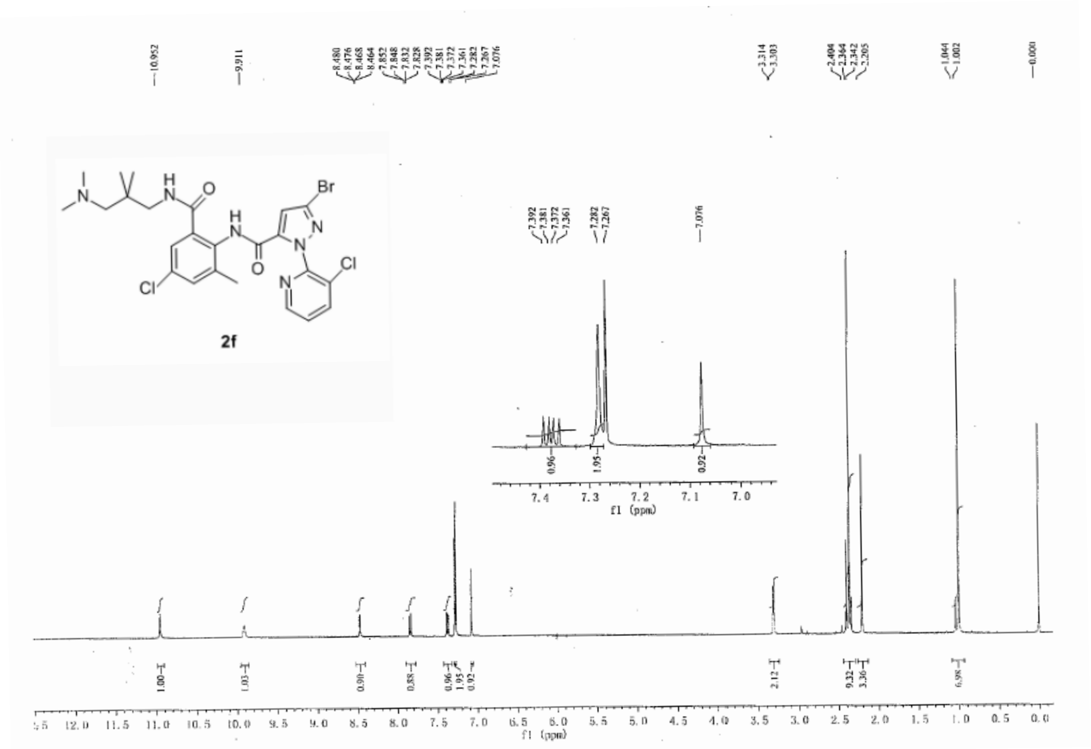

Figure S6. ^{1}H-NMR spectrum of compound **2f**.

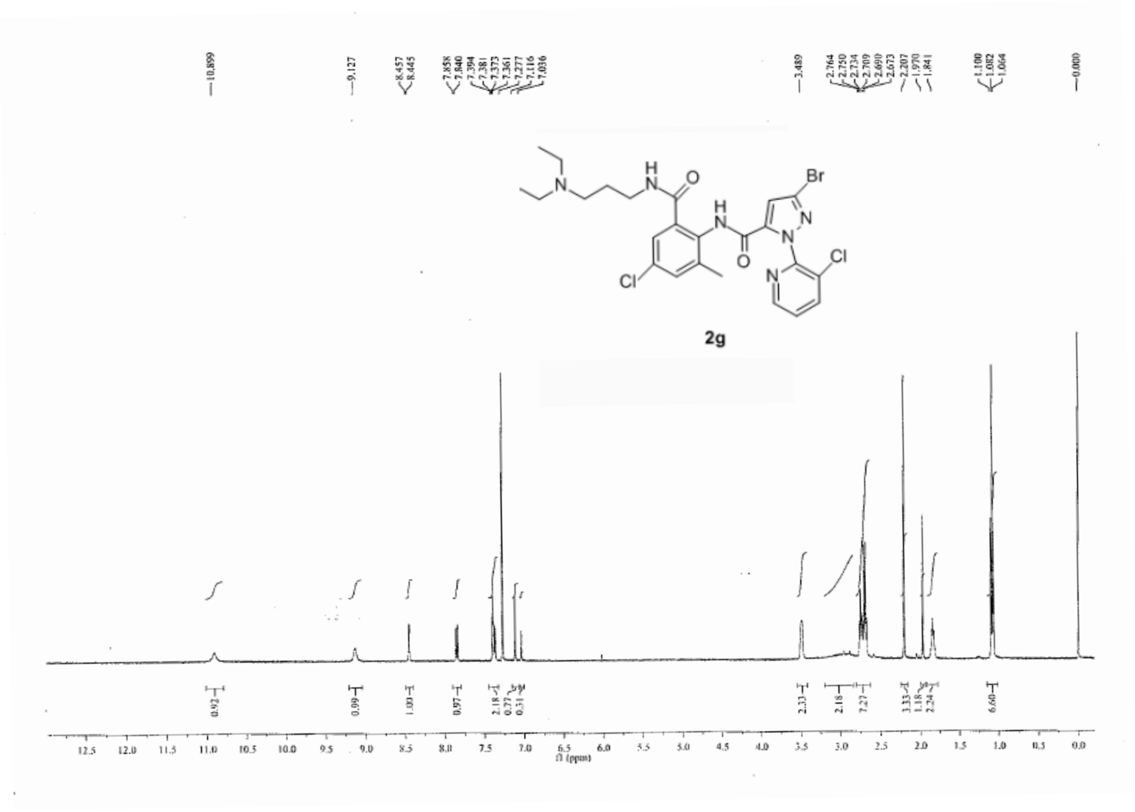

Figure S7. ^{1}H-NMR spectrum of compound **2g**.

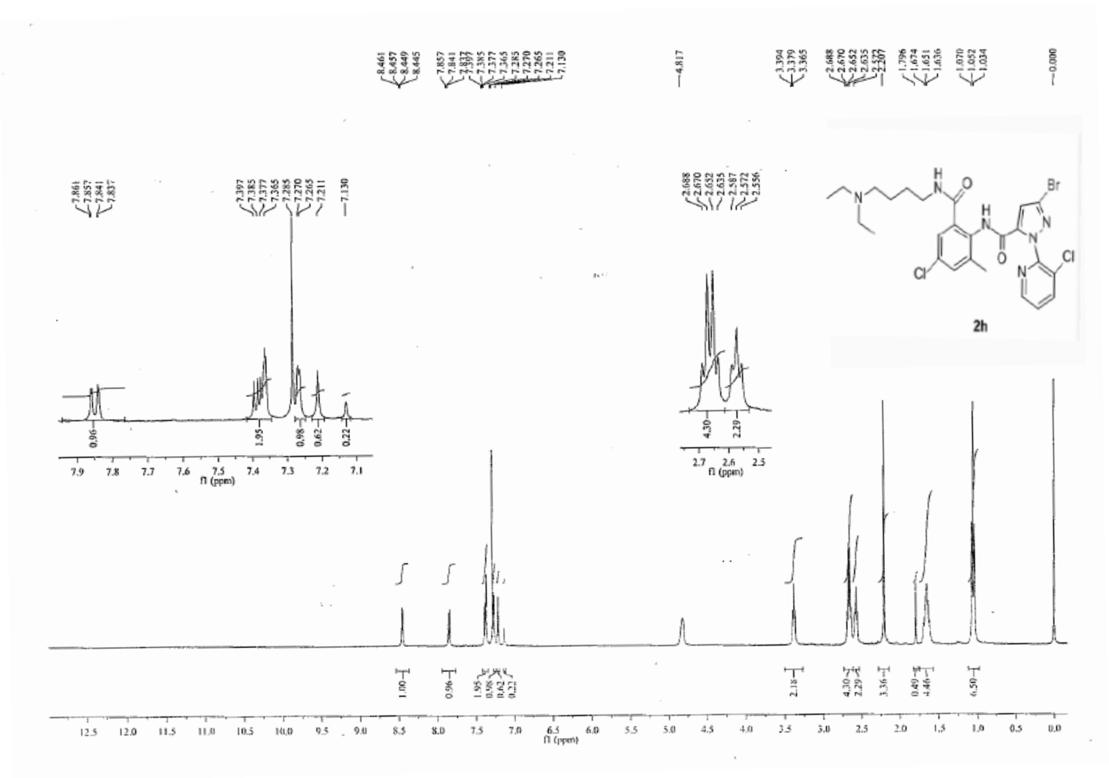

Figure S8. ^{1}H-NMR spectrum of compound **2h**.

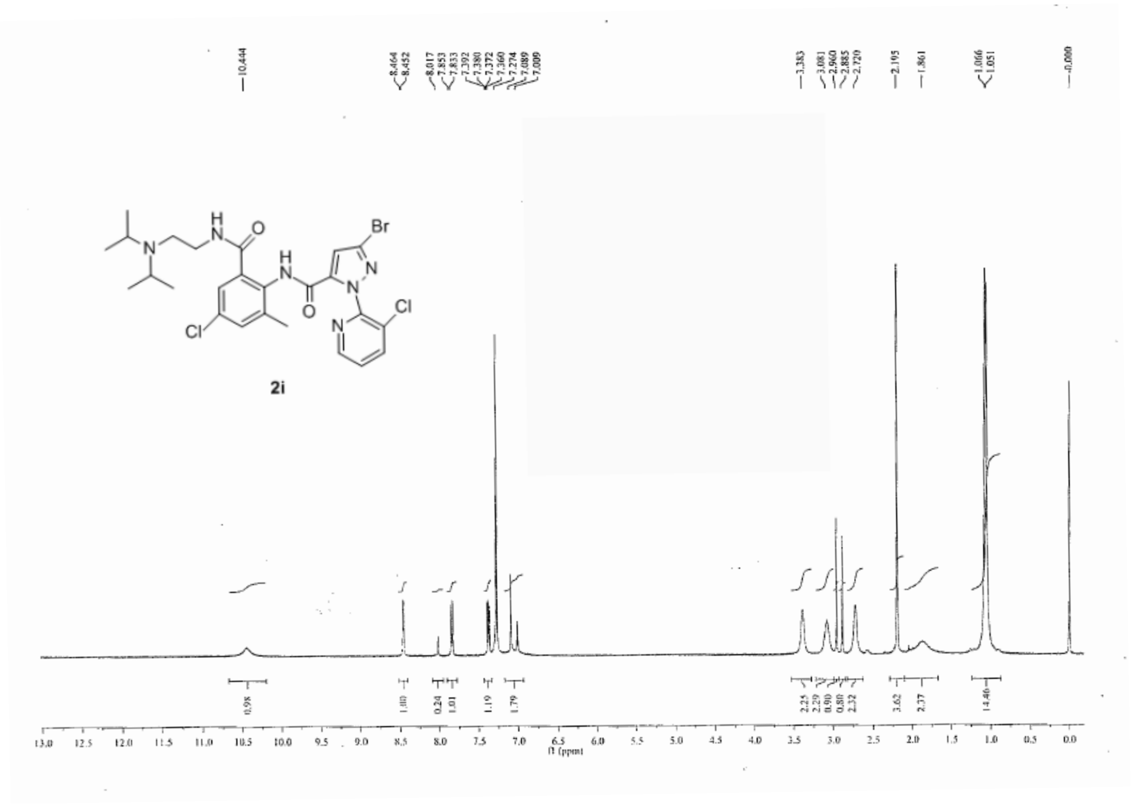

Figure S9. ^{1}H-NMR spectrum of compound **2i**.

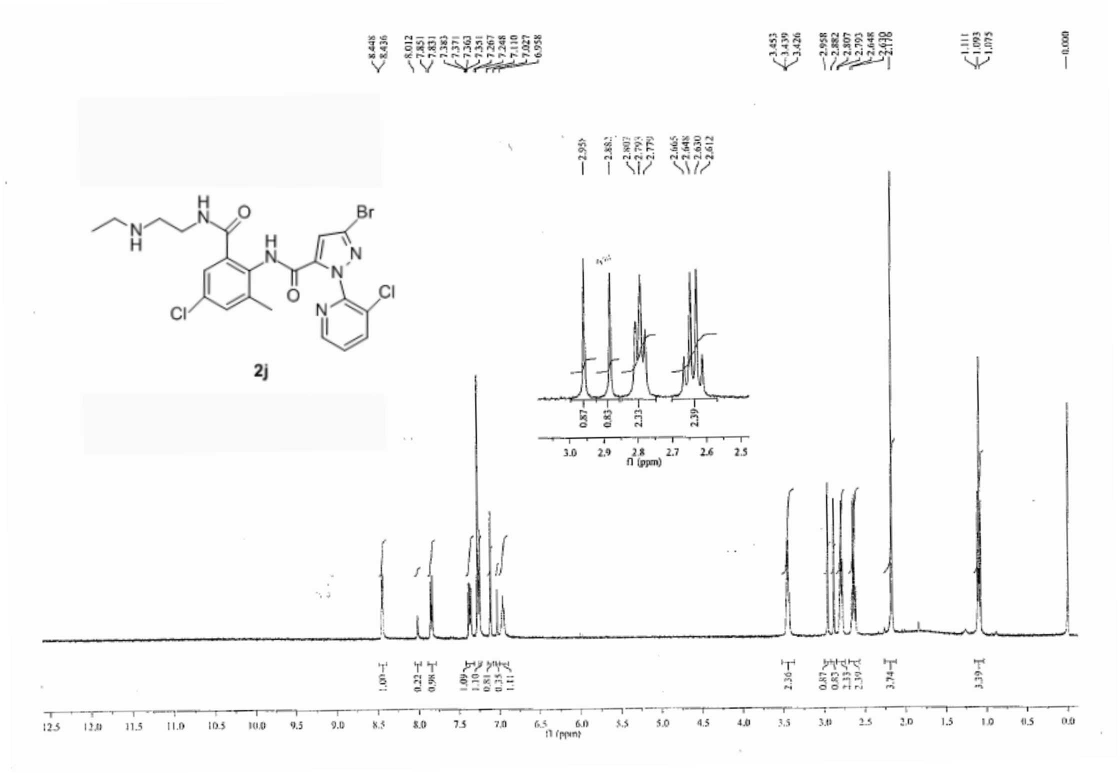

Figure S10. ^{1}H-NMR spectrum of compound **2j**.

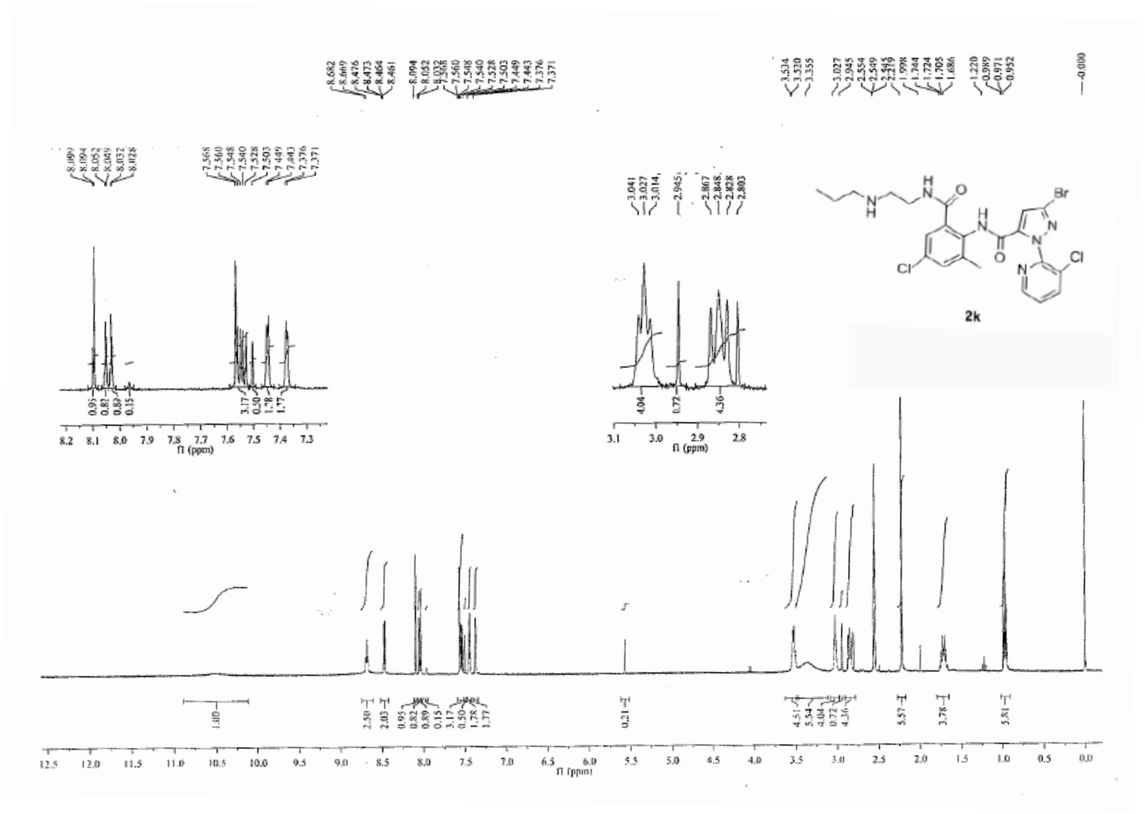

Figure S11. ^{1}H-NMR spectrum of compound **2k**.

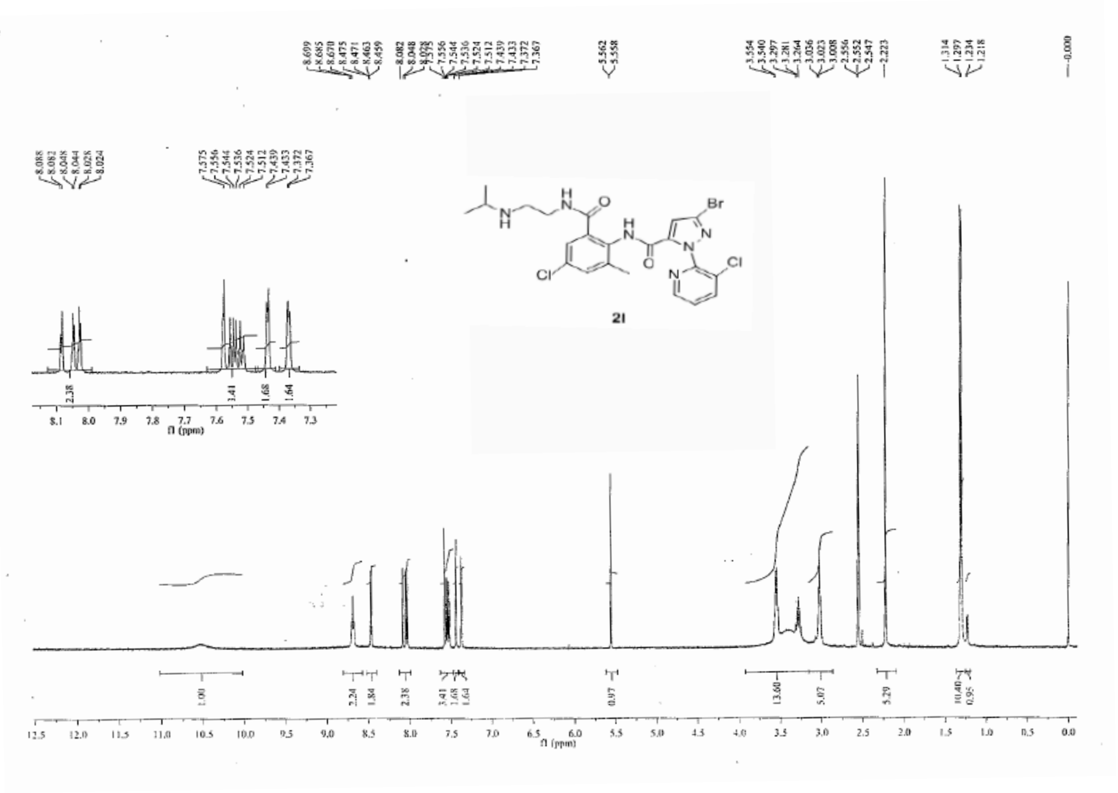

Figure S12. ^{1}H-NMR spectrum of compound **2l**.

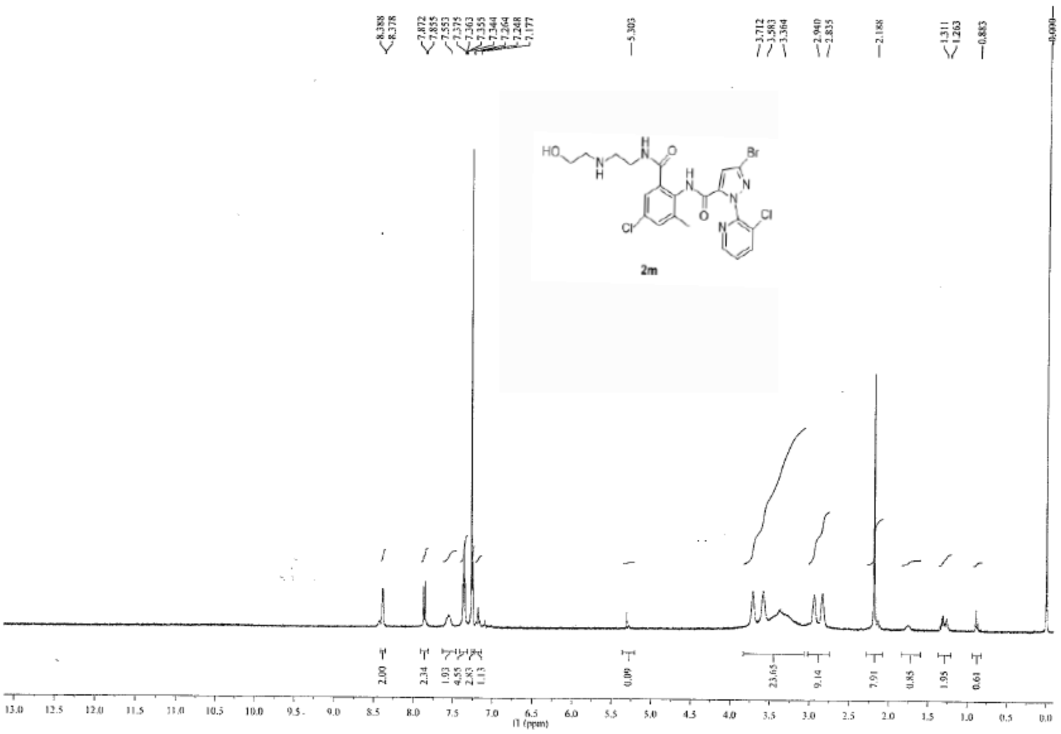

Figure S13. ^{1}H-NMR spectrum of compound **2m**.

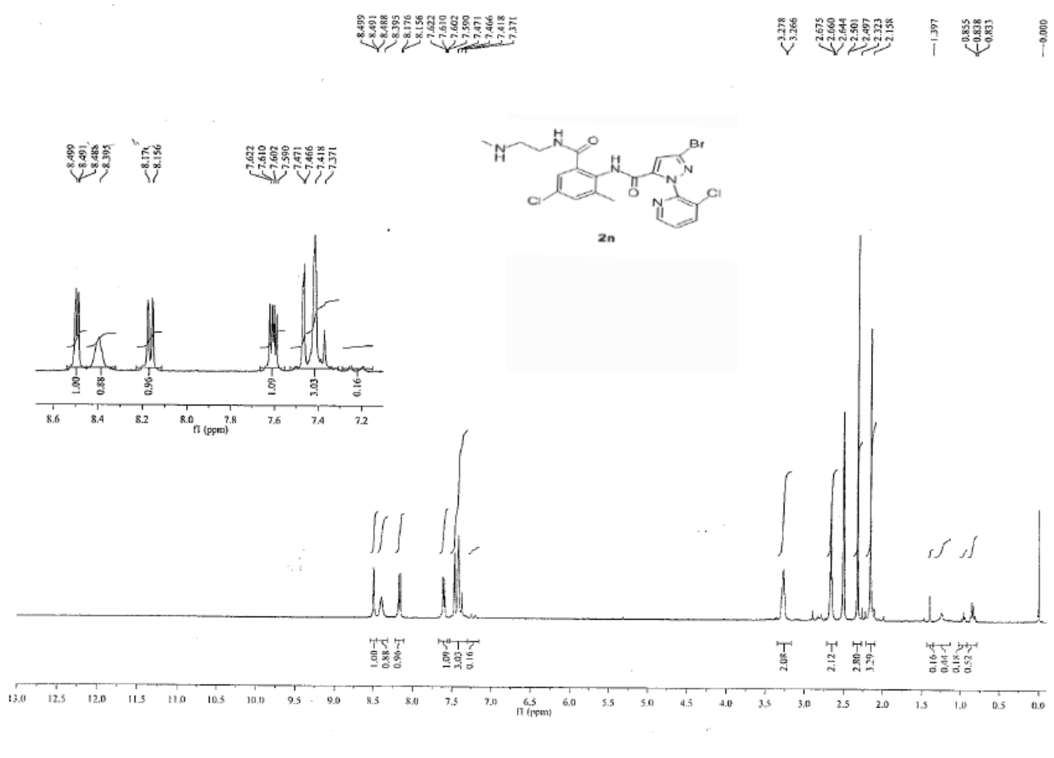

Figure S14. ^{1}H-NMR spectrum of compound **2n**.

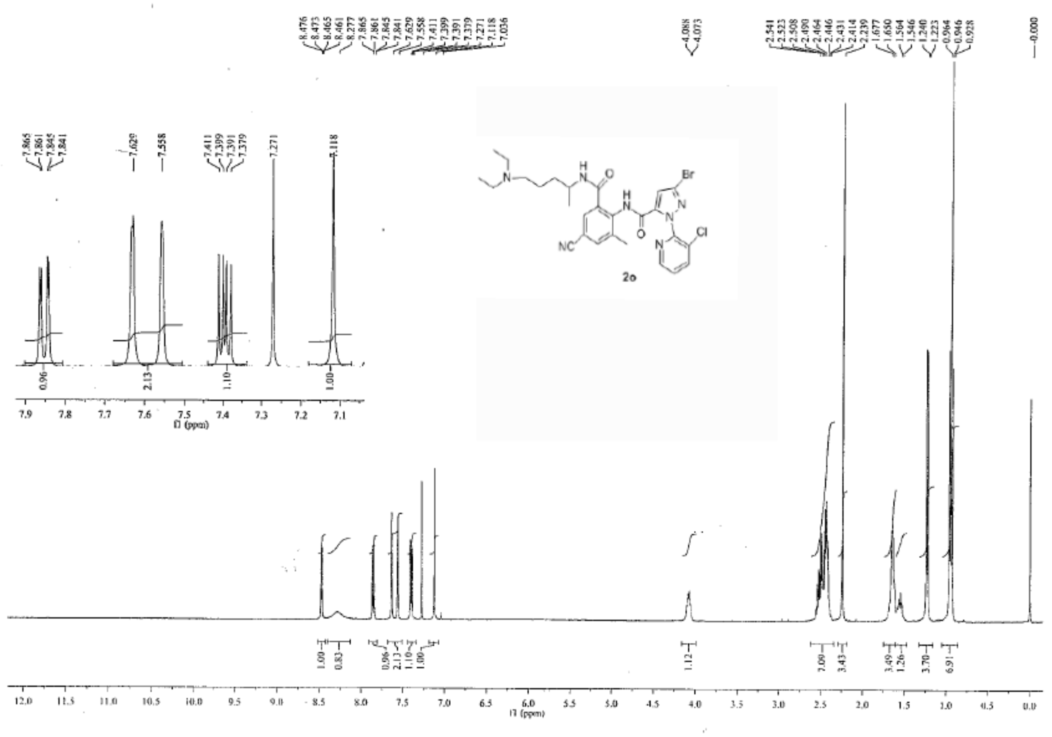

Figure S15. ^{1}H-NMR spectrum of compound **2o**.

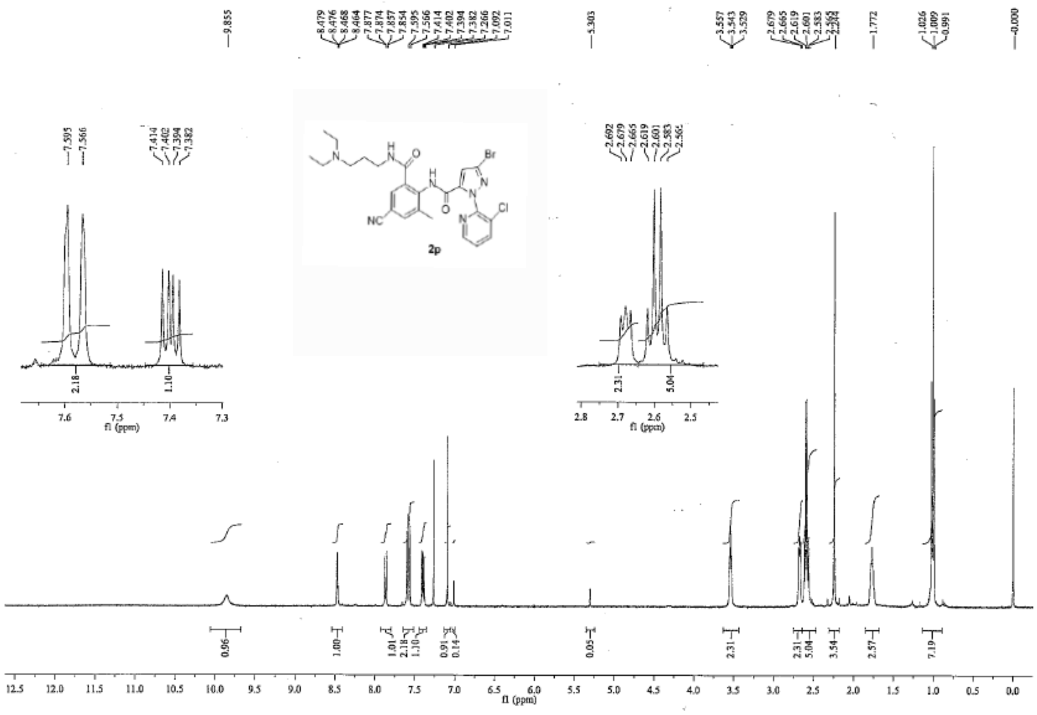

Figure S16. ^{1}H-NMR spectrum of compound **2p**.

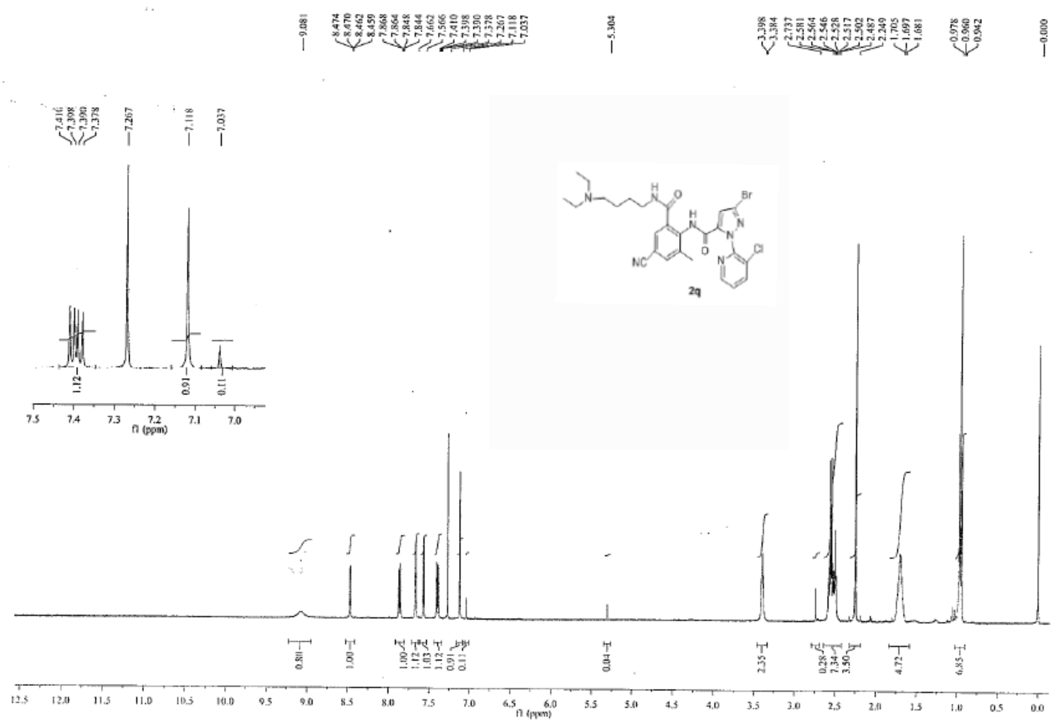

Figure S17. ^{1}H-NMR spectrum of compound **2q**.

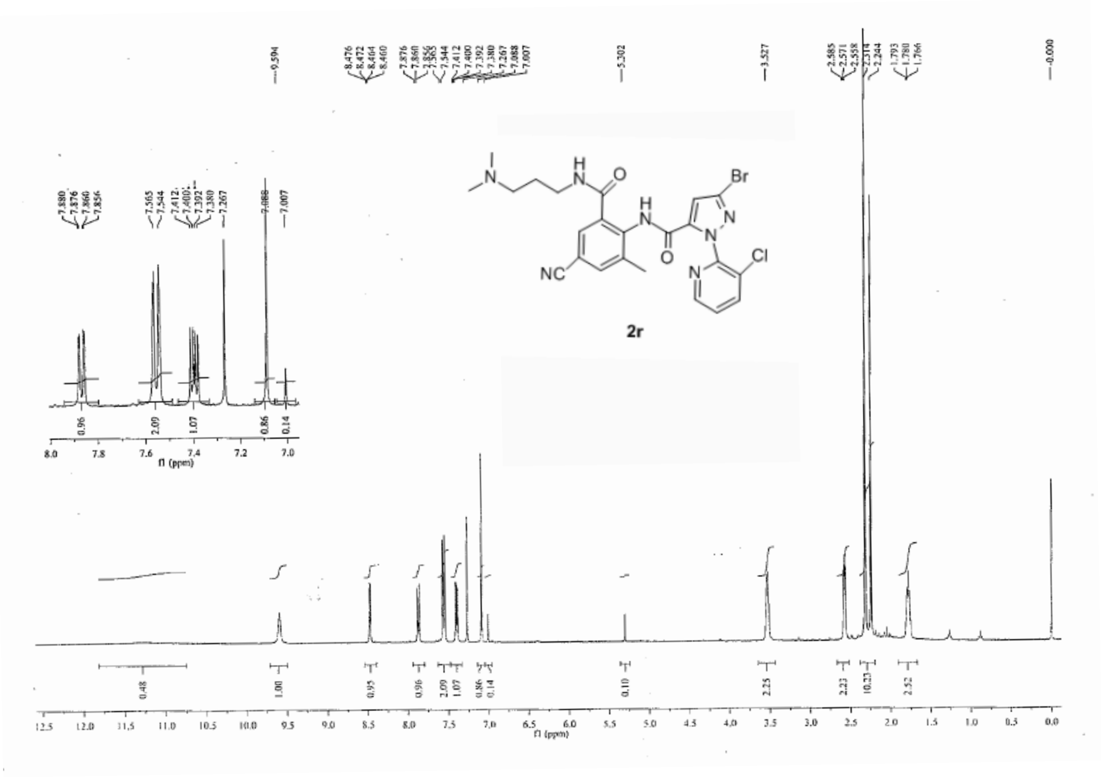

Figure S18. ^{1}H-NMR spectrum of compound **2r**.

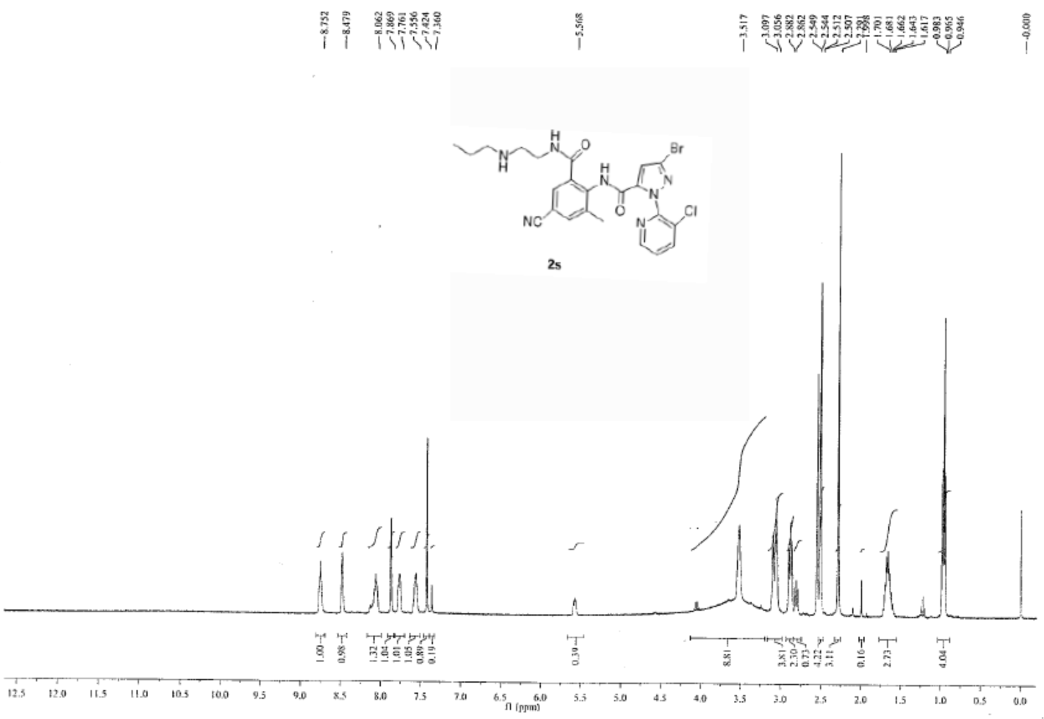

Figure S19. ^{1}H-NMR spectrum of compound **2s**.

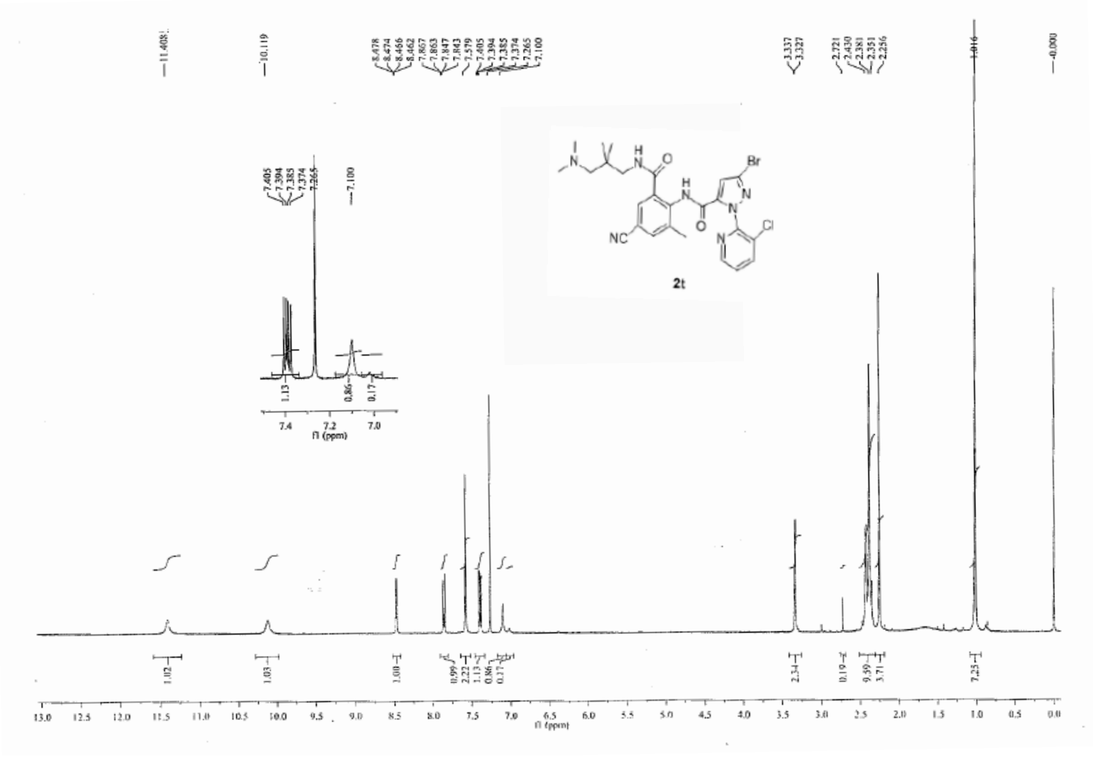

Figure S20. ^{1}H-NMR spectrum of compound **2t**.

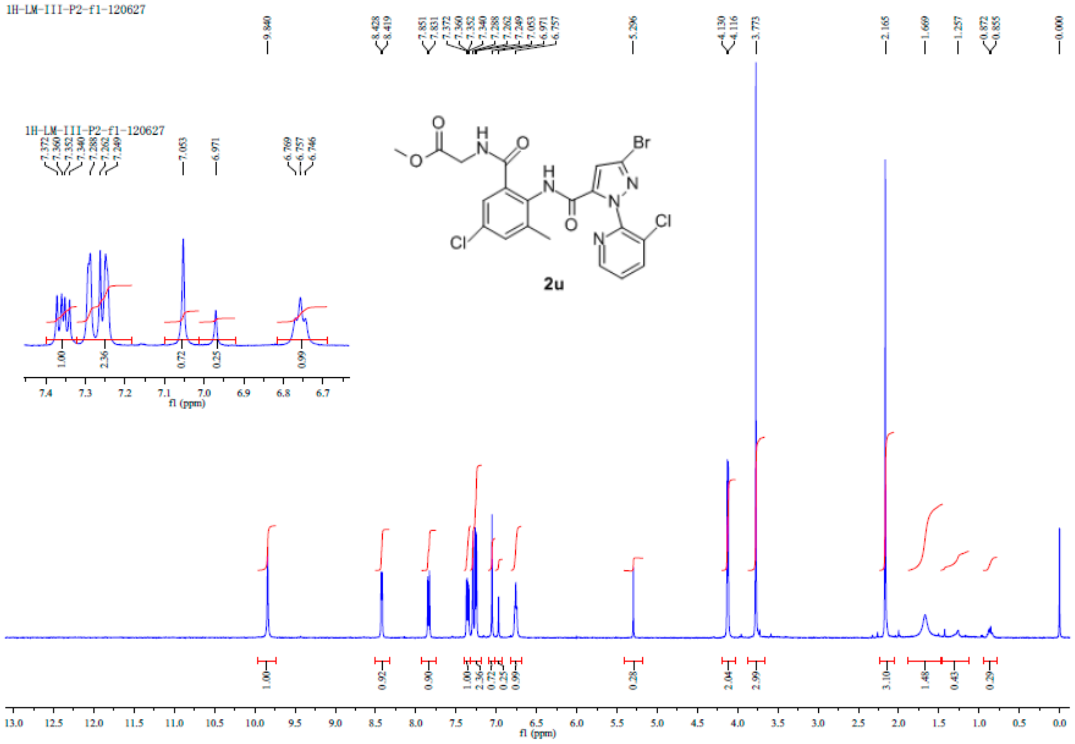

Figure S21. ^{1}H-NMR spectrum of compound **2u**.

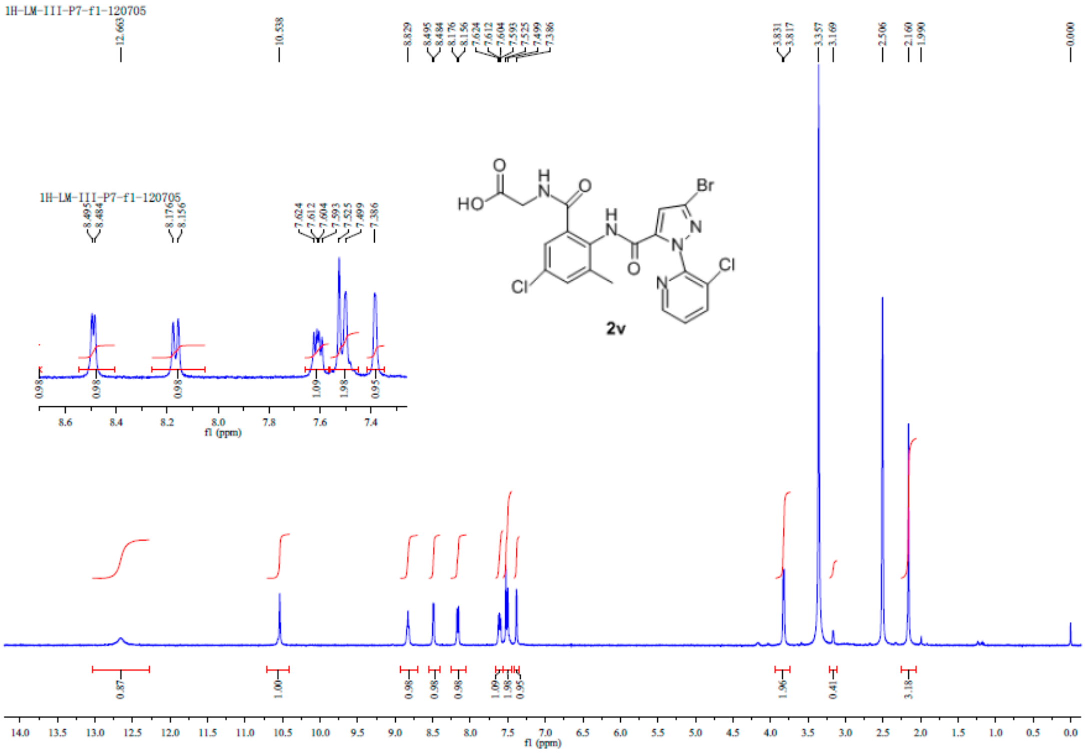

Figure S22. ^{1}H-NMR spectrum of compound **2v**.

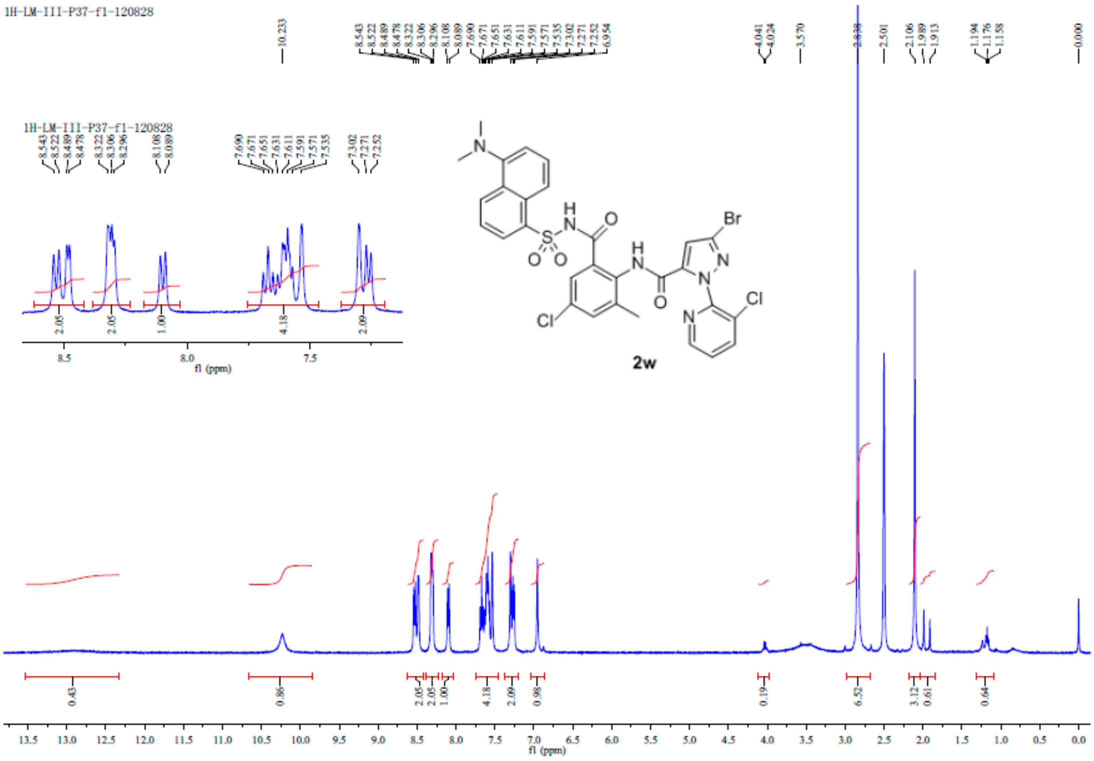

2w

Figure S23. ^{1}H-NMR spectrum of compound **2w**.

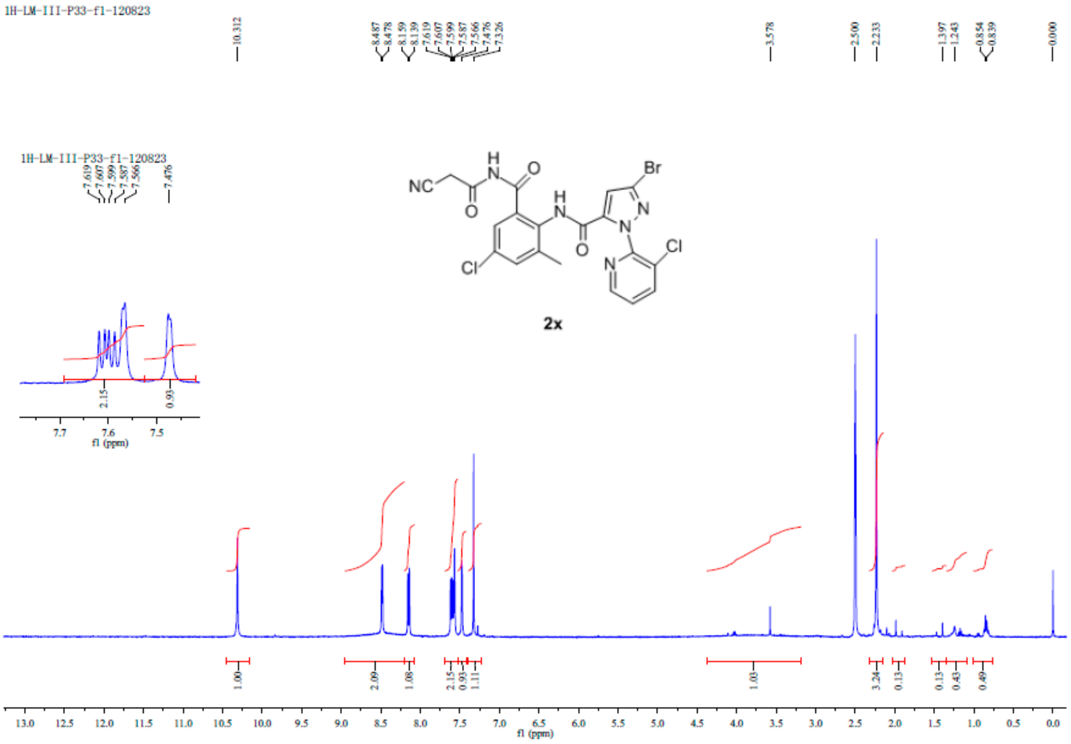

2x

Figure S24. ^{1}H-NMR spectrum of compound **2x**.

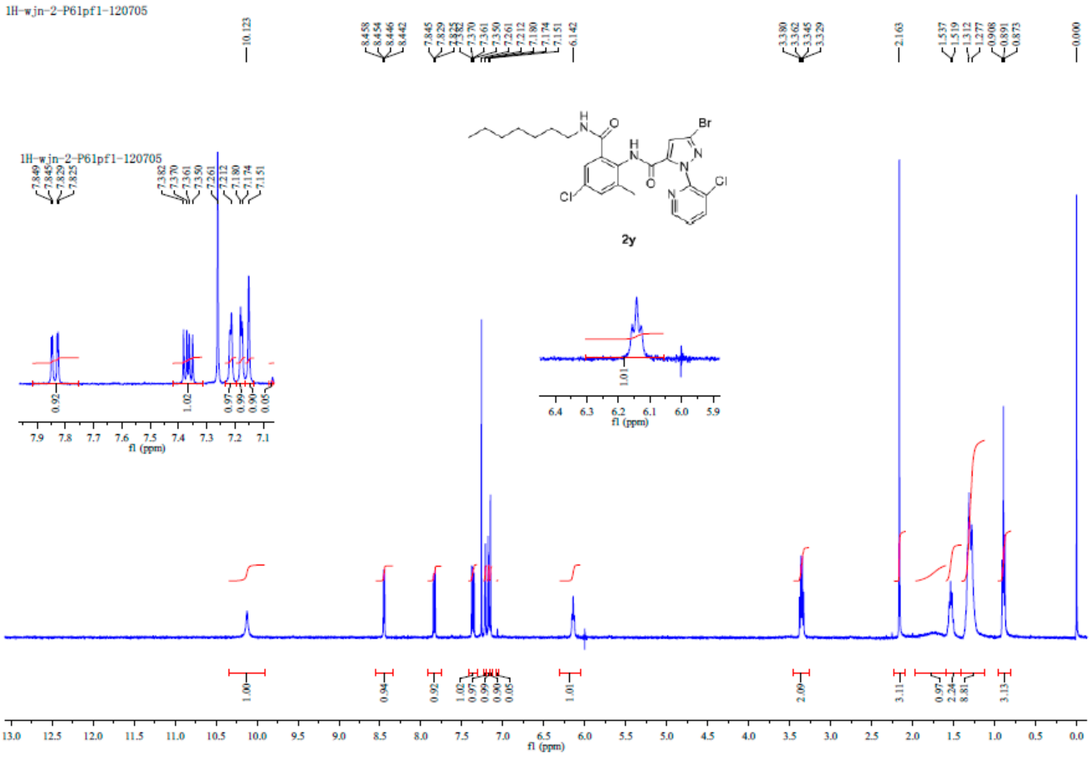

Figure S25. ^{1}H-NMR spectrum of compound **2y**.

2. C-NMR

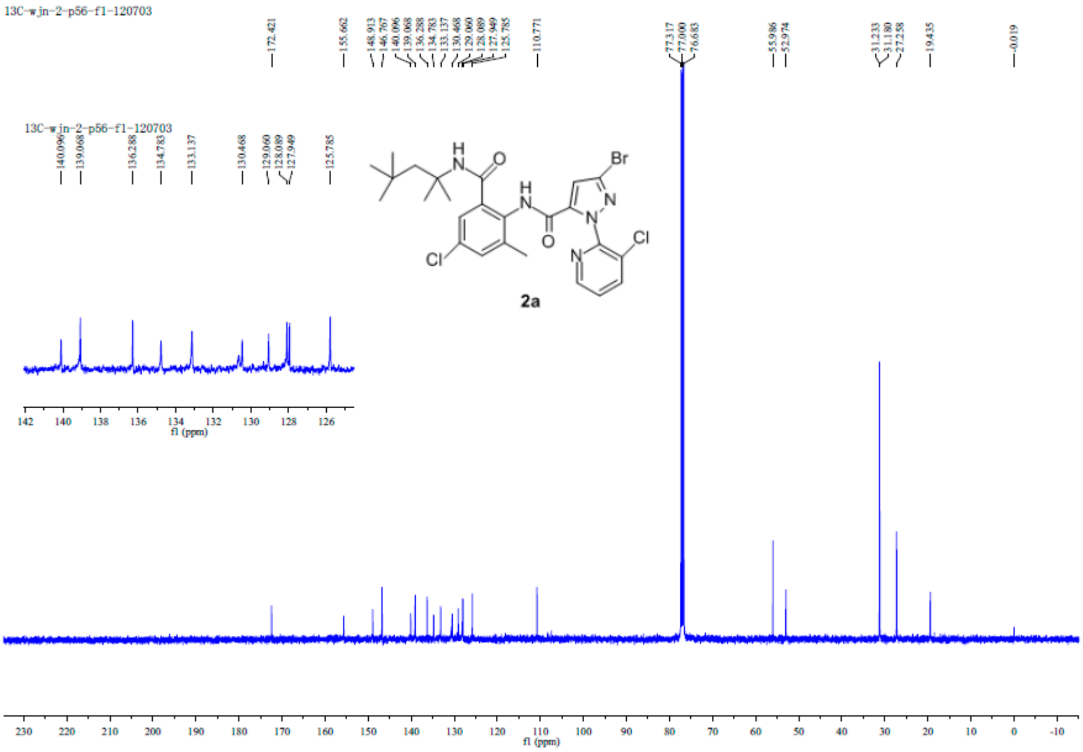

Figure S26. ^{13}C-NMR spectrum of compound **2a**.

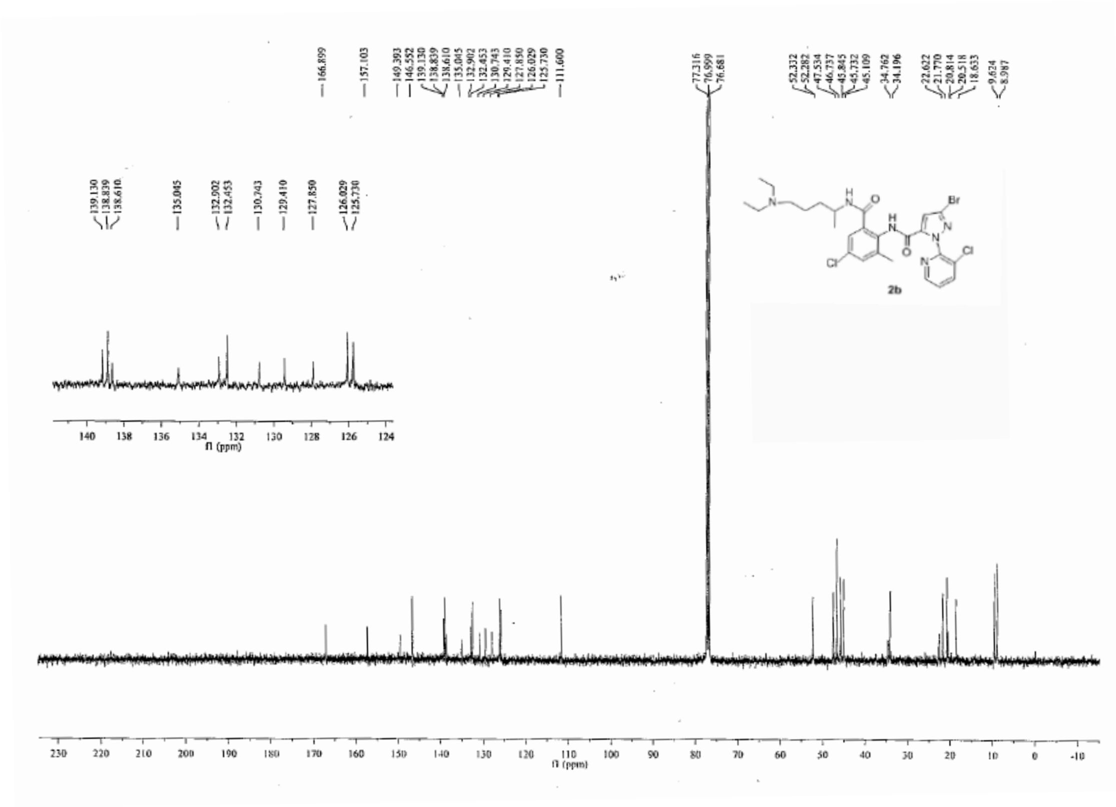

Figure S27. ^{13}C-NMR spectrum of compound **2b**.

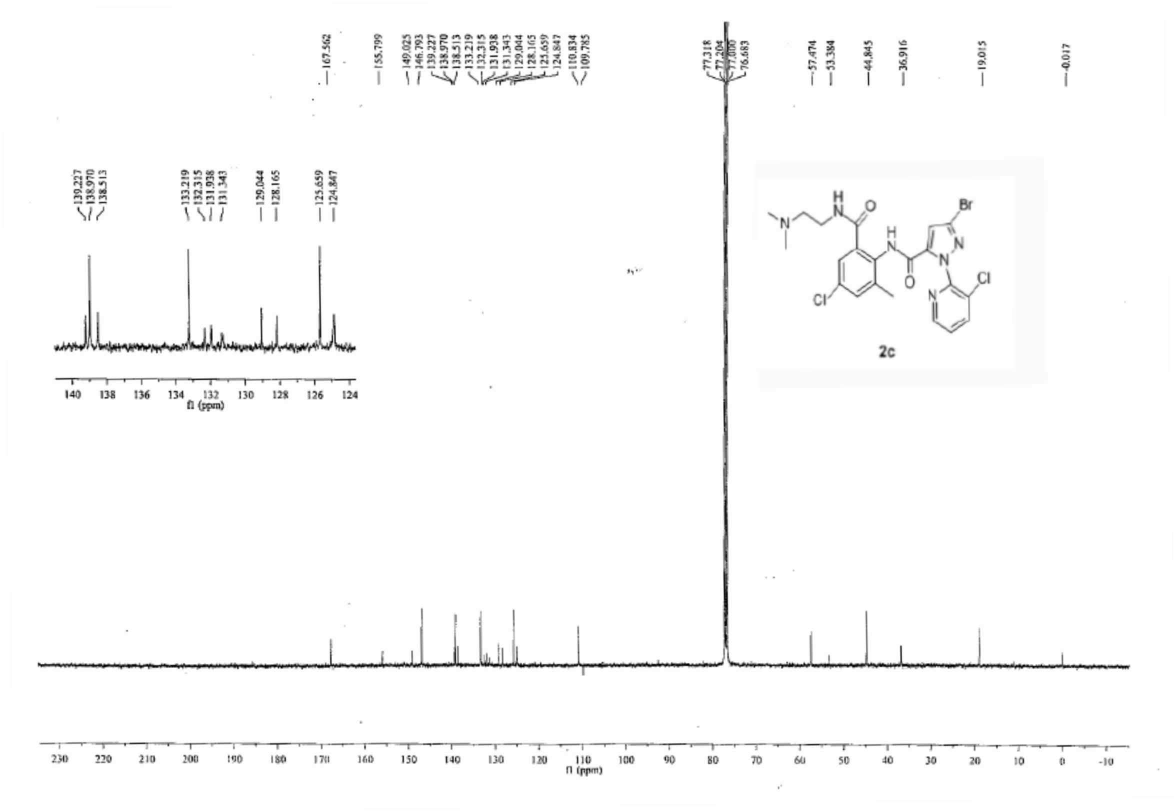

Figure S28. ^{13}C-NMR spectrum of compound **2c**.

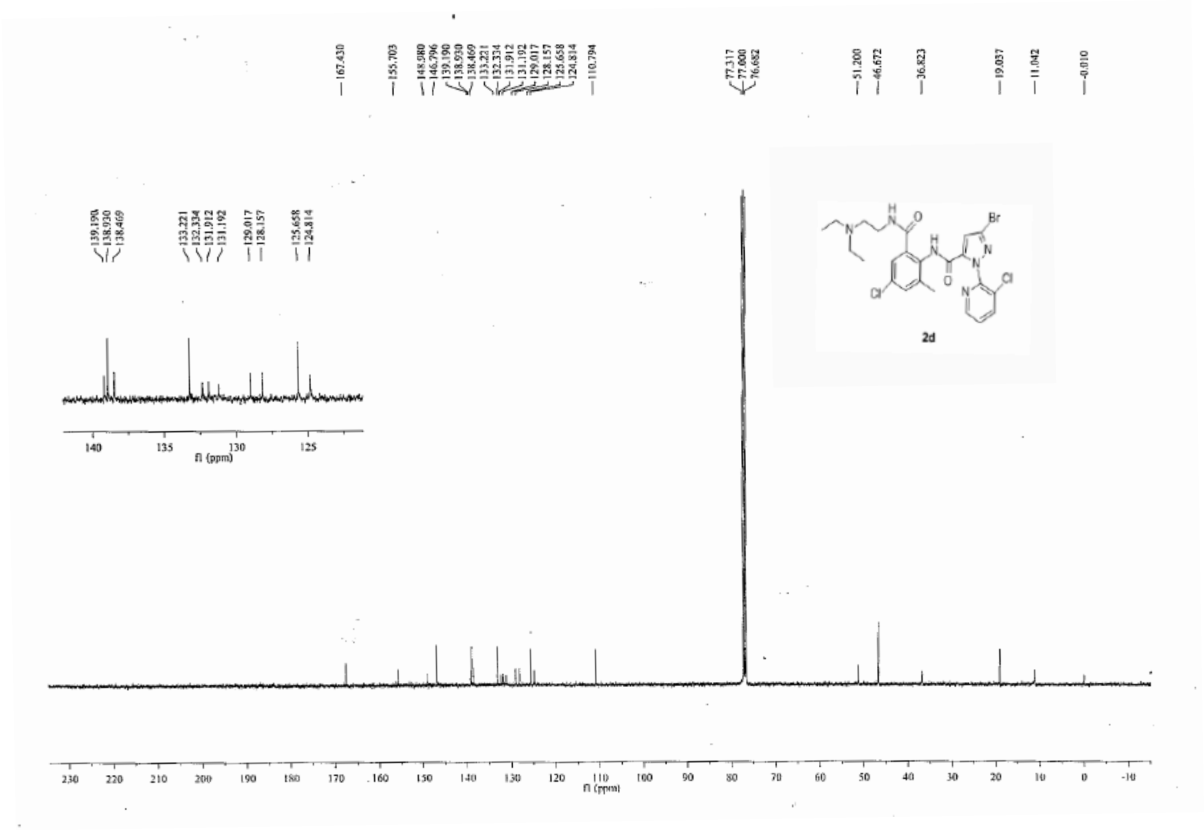

Figure S29. ^{13}C-NMR spectrum of compound **2d**.

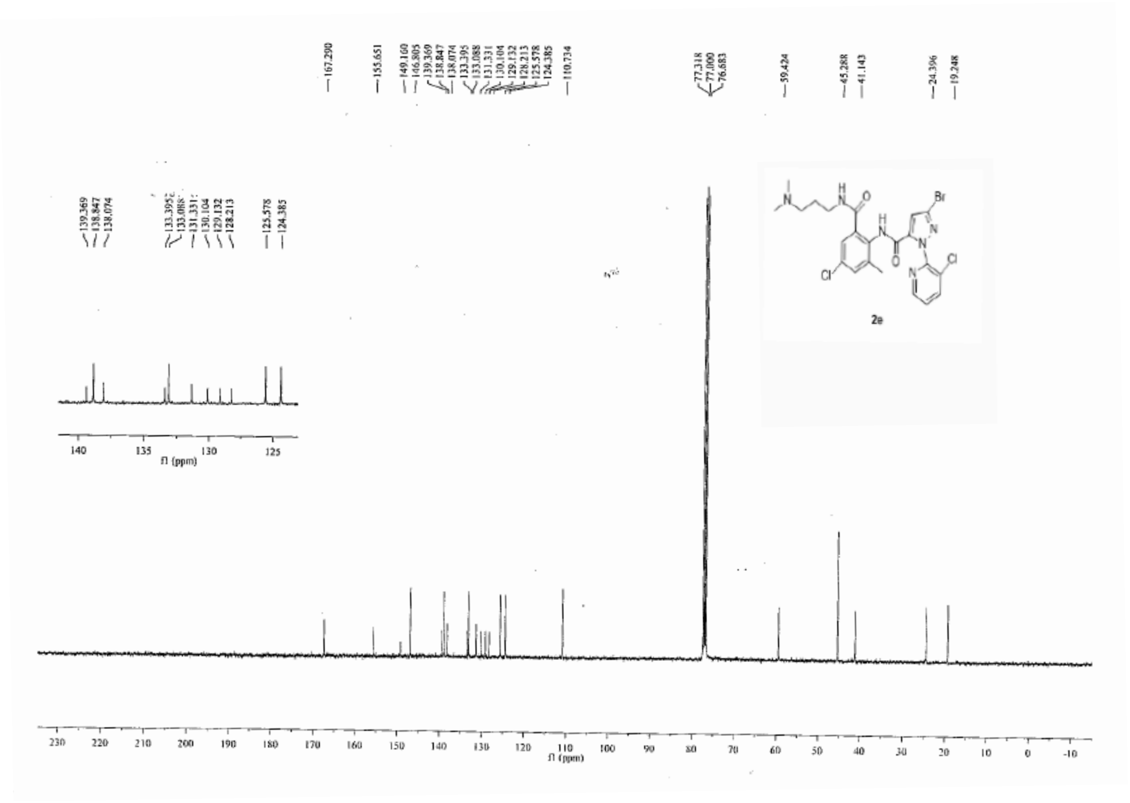

Figure S30. ^{13}C-NMR spectrum of compound **2e**.

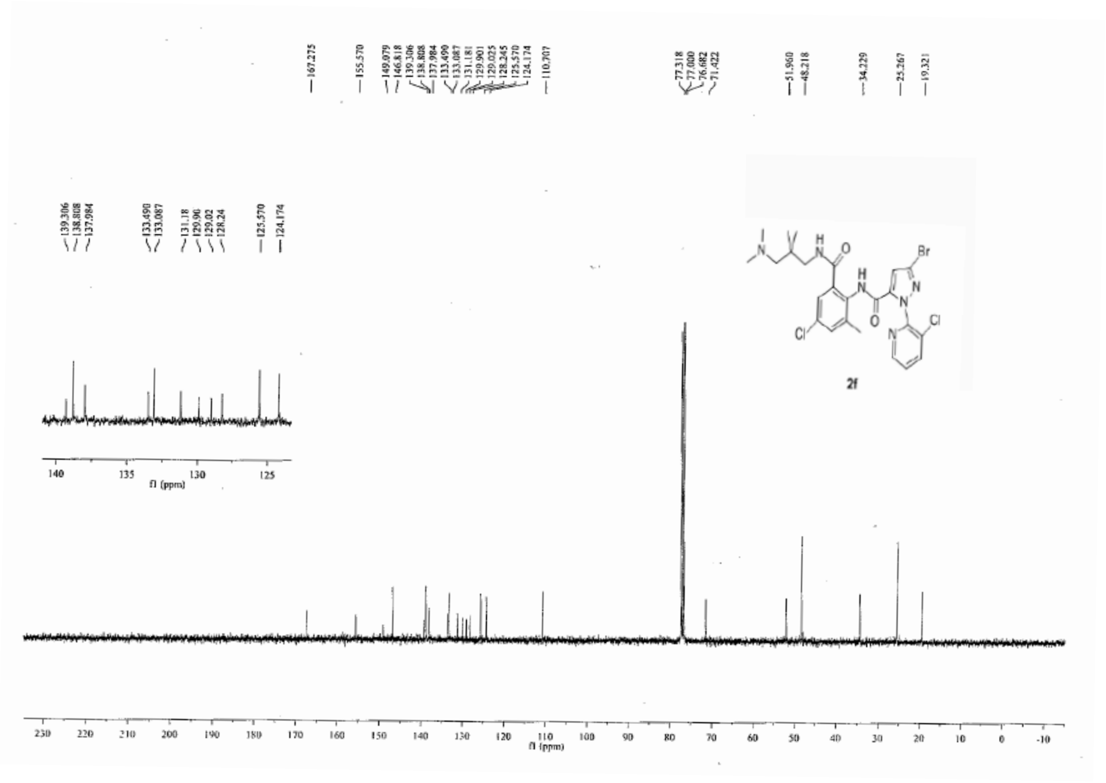

Figure S31. ^{13}C-NMR spectrum of compound **2f**.

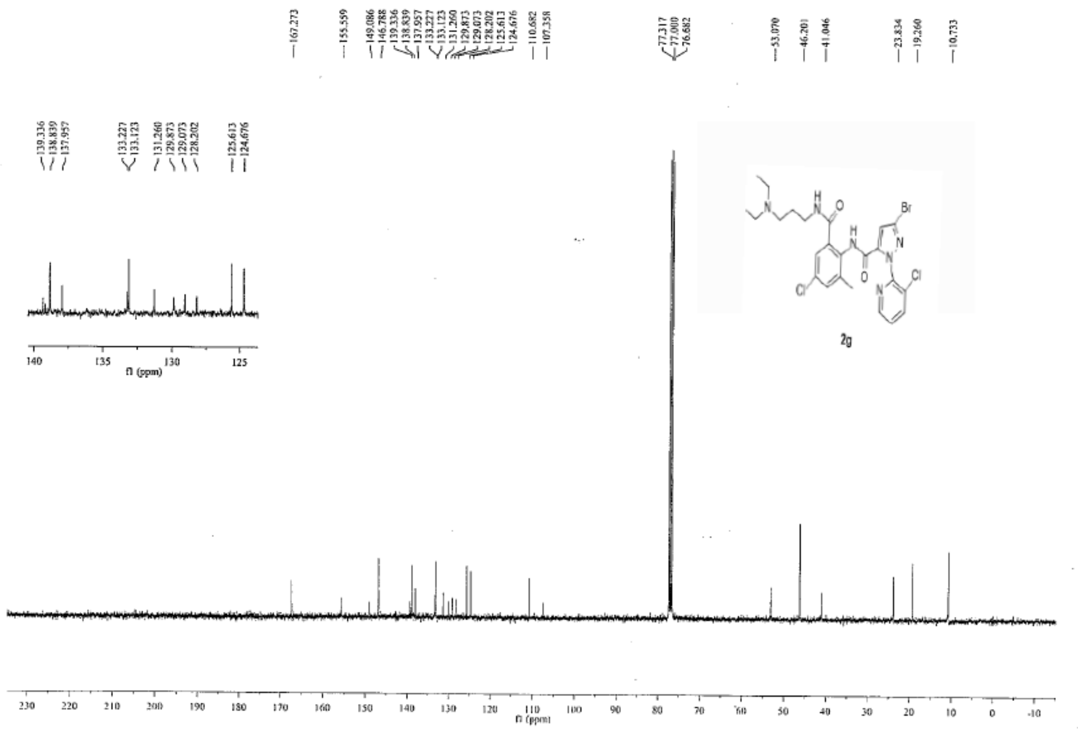

Figure S32. ^{13}C-NMR spectrum of compound **2g**.

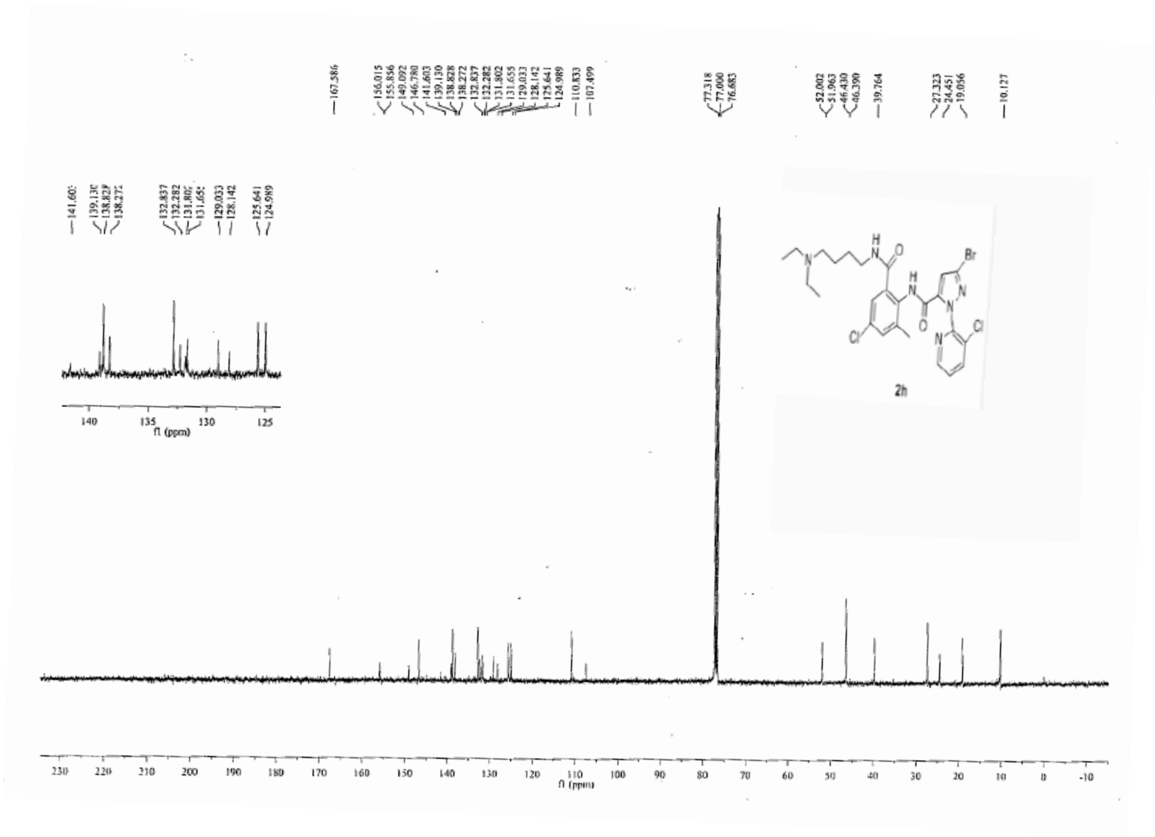

Figure S33. ^{13}C-NMR spectrum of compound **2h**.

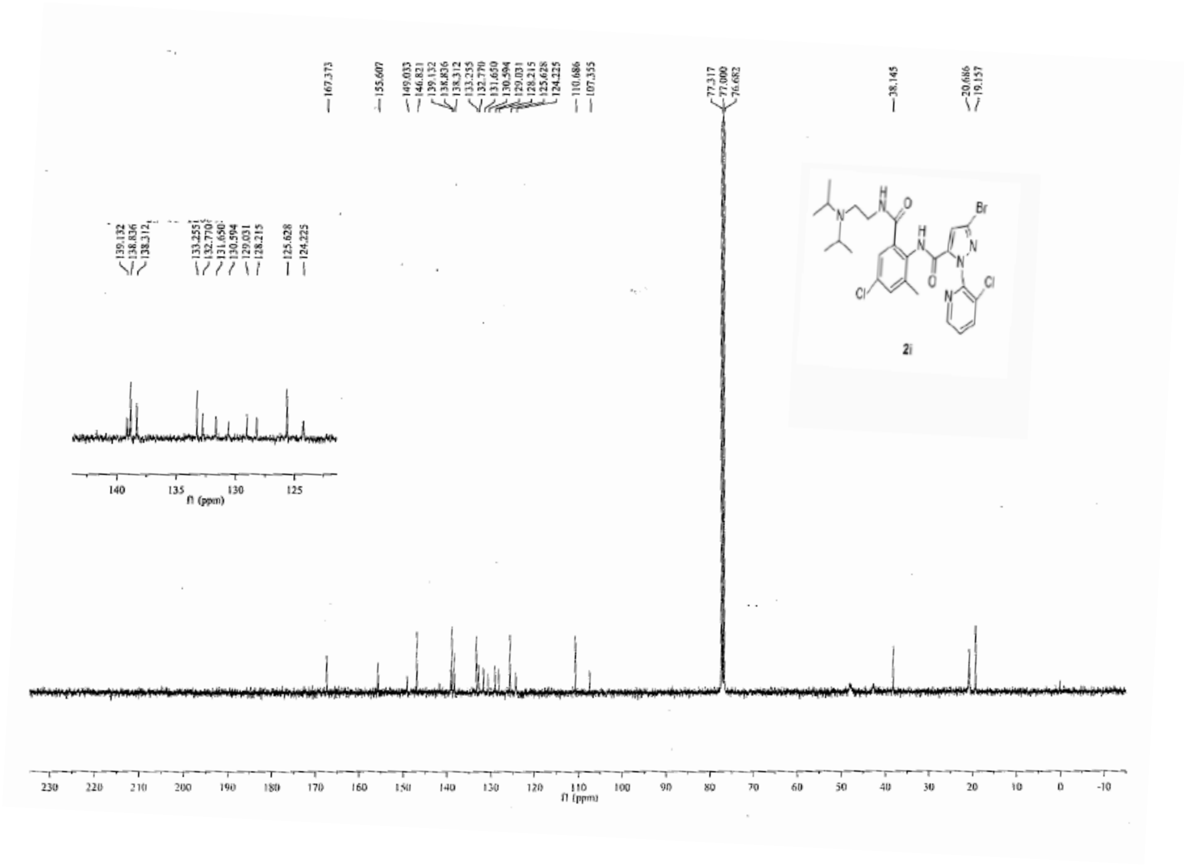

Figure S34. ^{13}C-NMR spectrum of compound **2i**.

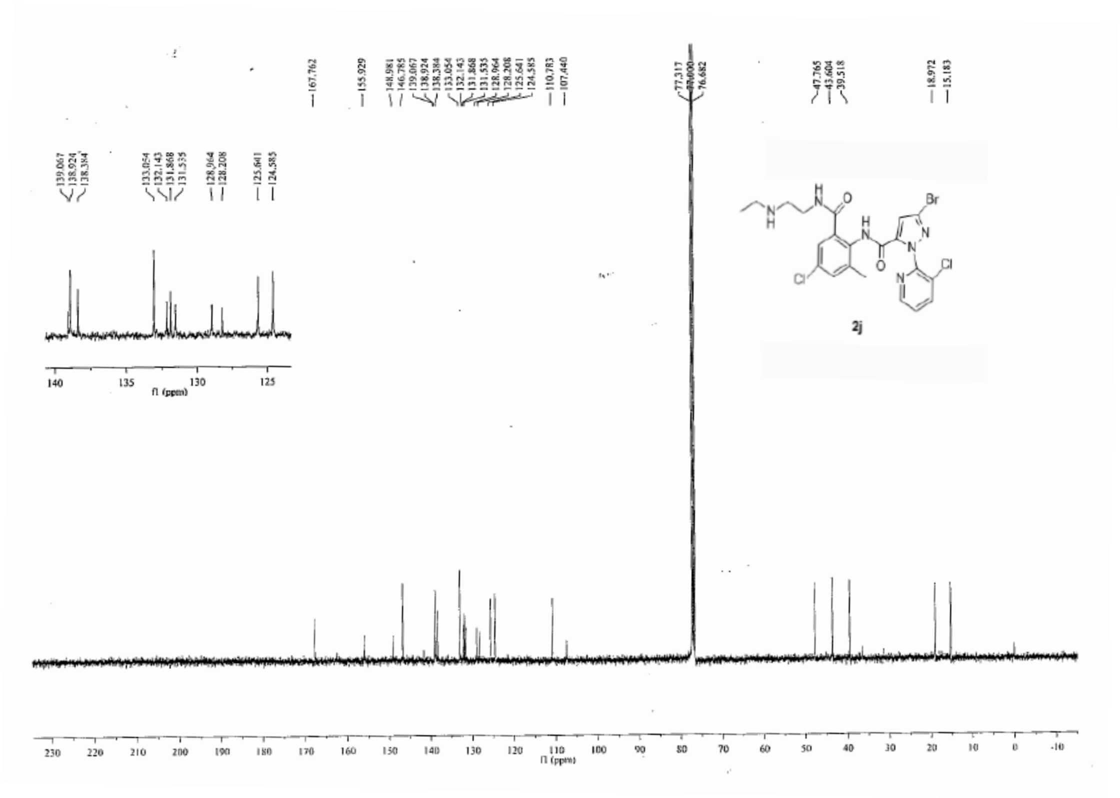

Figure S35. ^{13}C-NMR spectrum of compound **2j**.

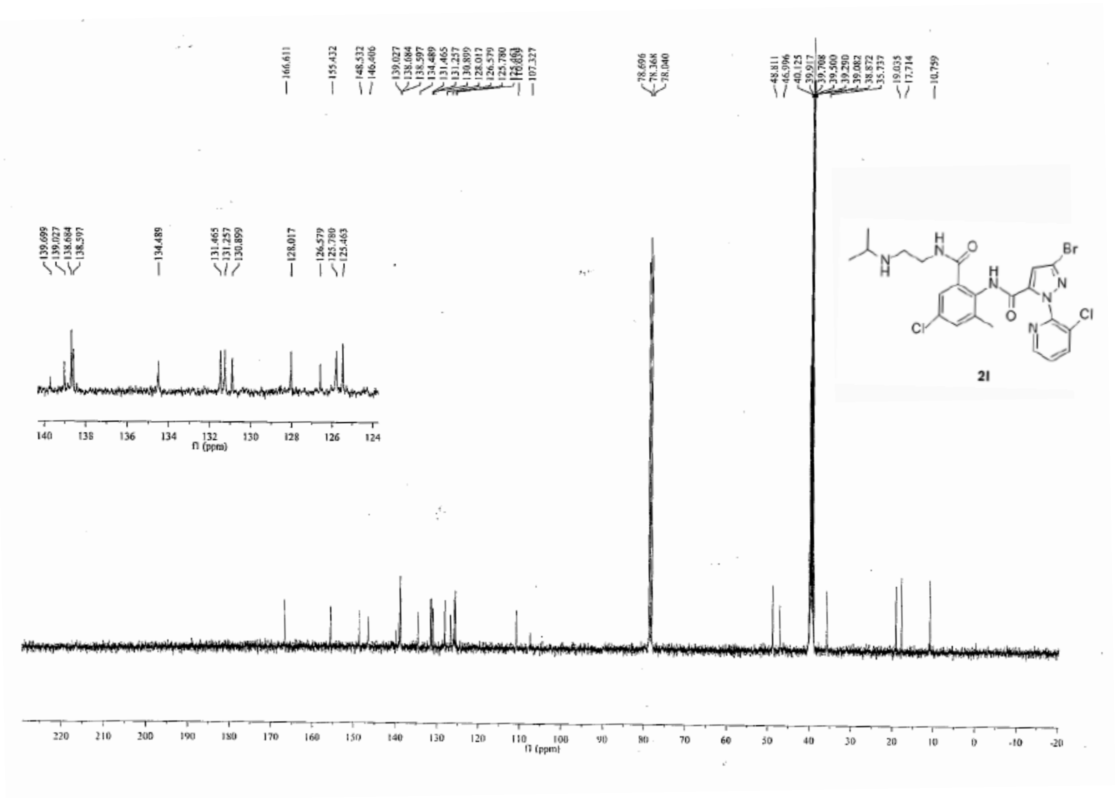

Figure S36. ^{13}C-NMR spectrum of compound **2k**.

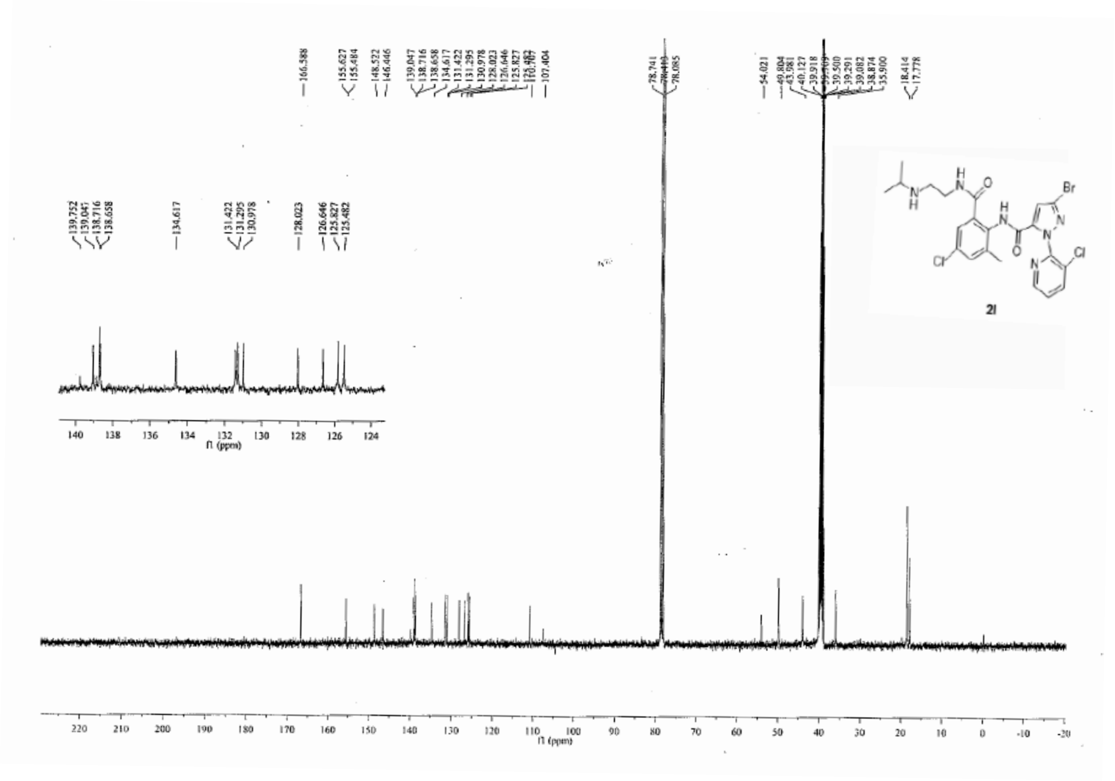

Figure S37. ^{13}C-NMR spectrum of compound **2l**.

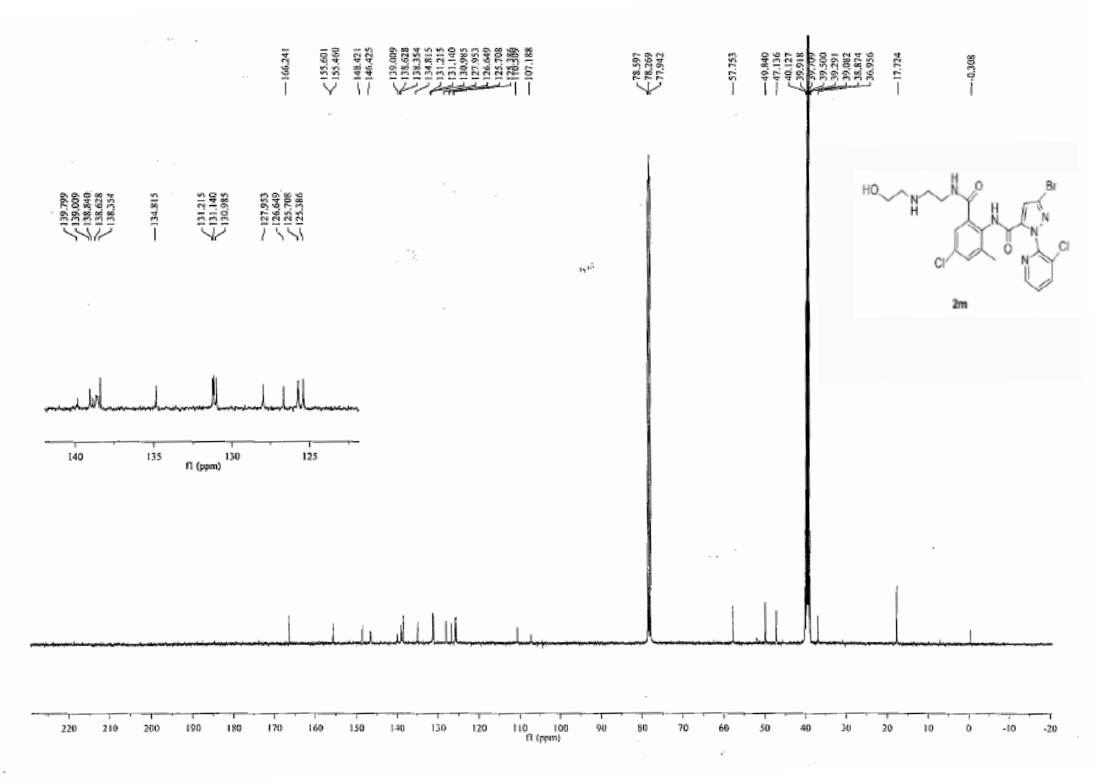

Figure S38. ^{13}C-NMR spectrum of compound **2m**.

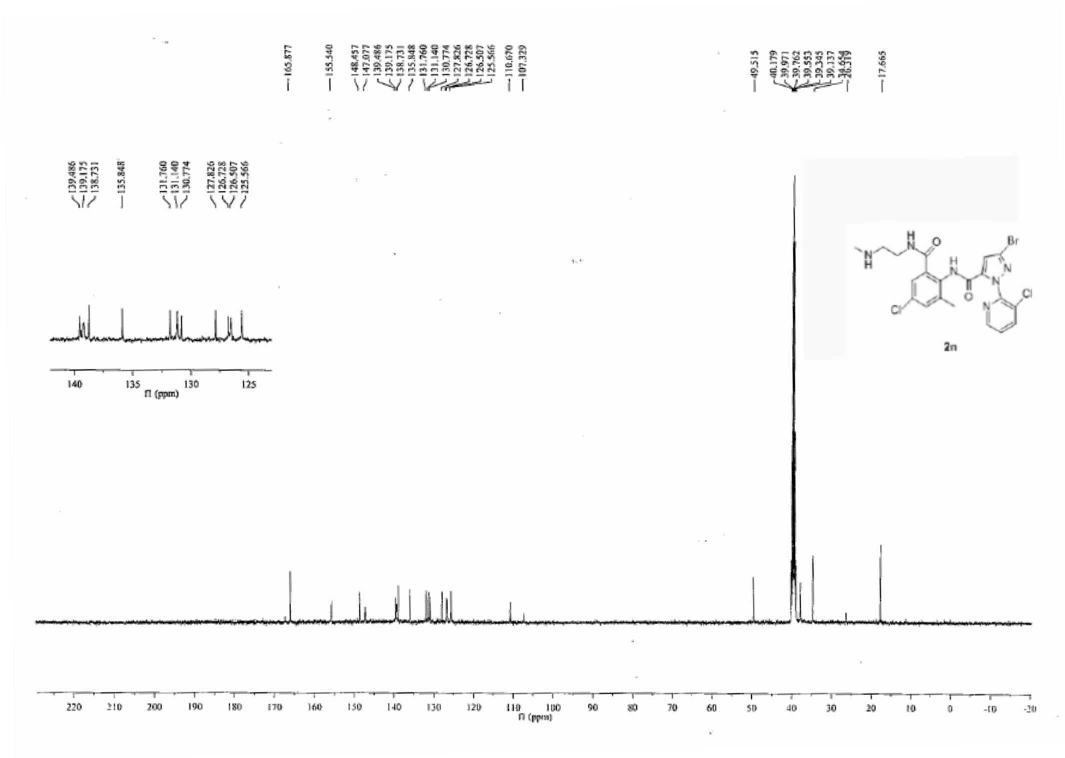

Figure S39. ^{13}C-NMR spectrum of compound **2n**.

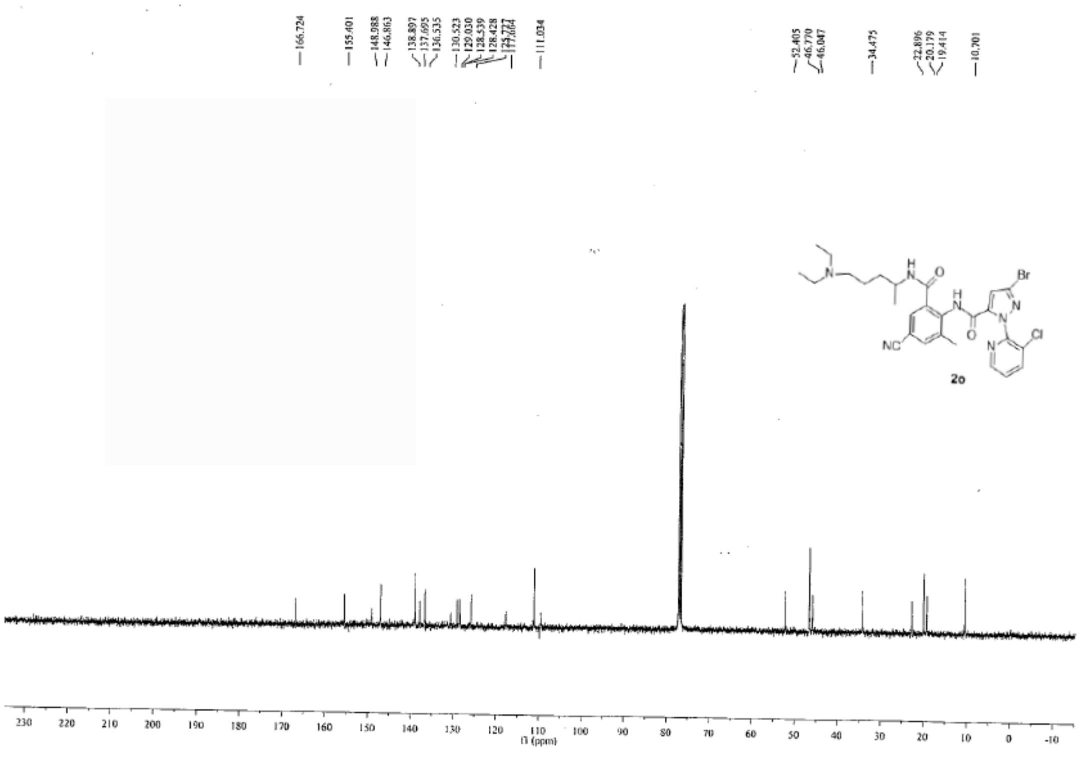

Figure S40. ^{13}C-NMR spectrum of compound **2o**.

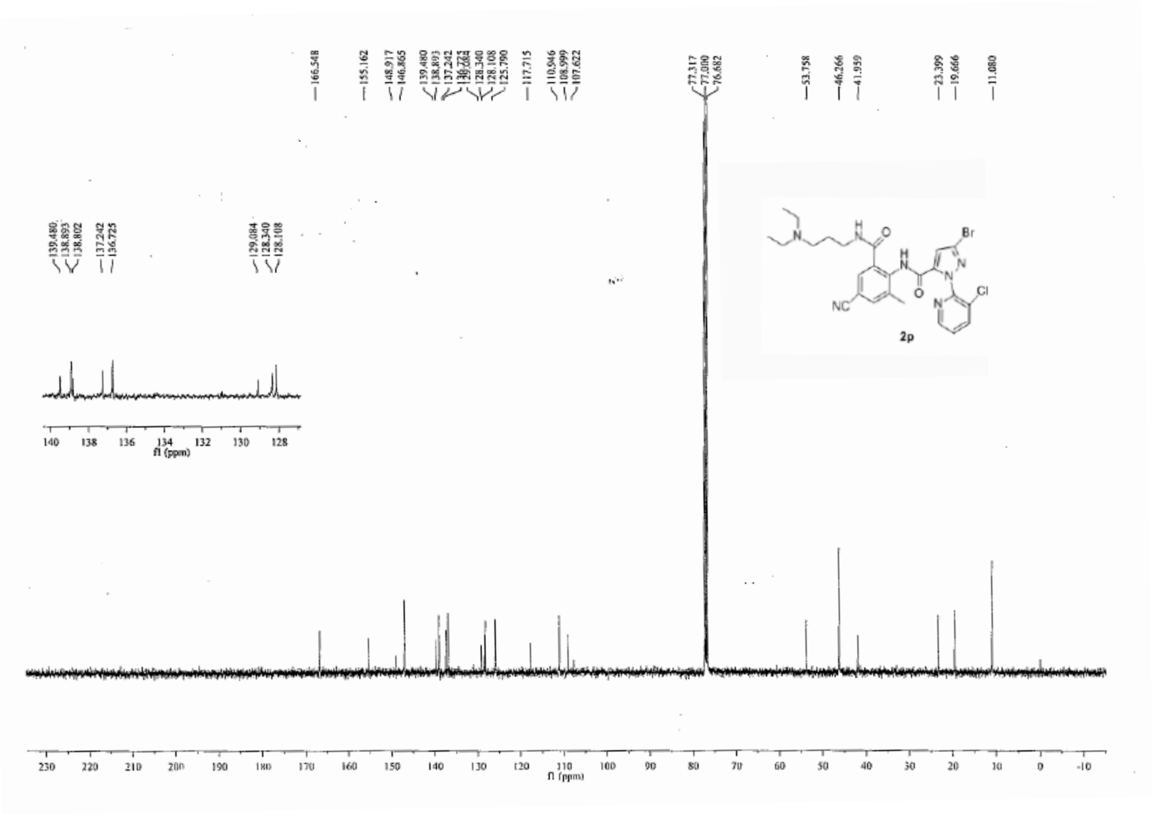

Figure S41. ^{13}C-NMR spectrum of compound **2p**.

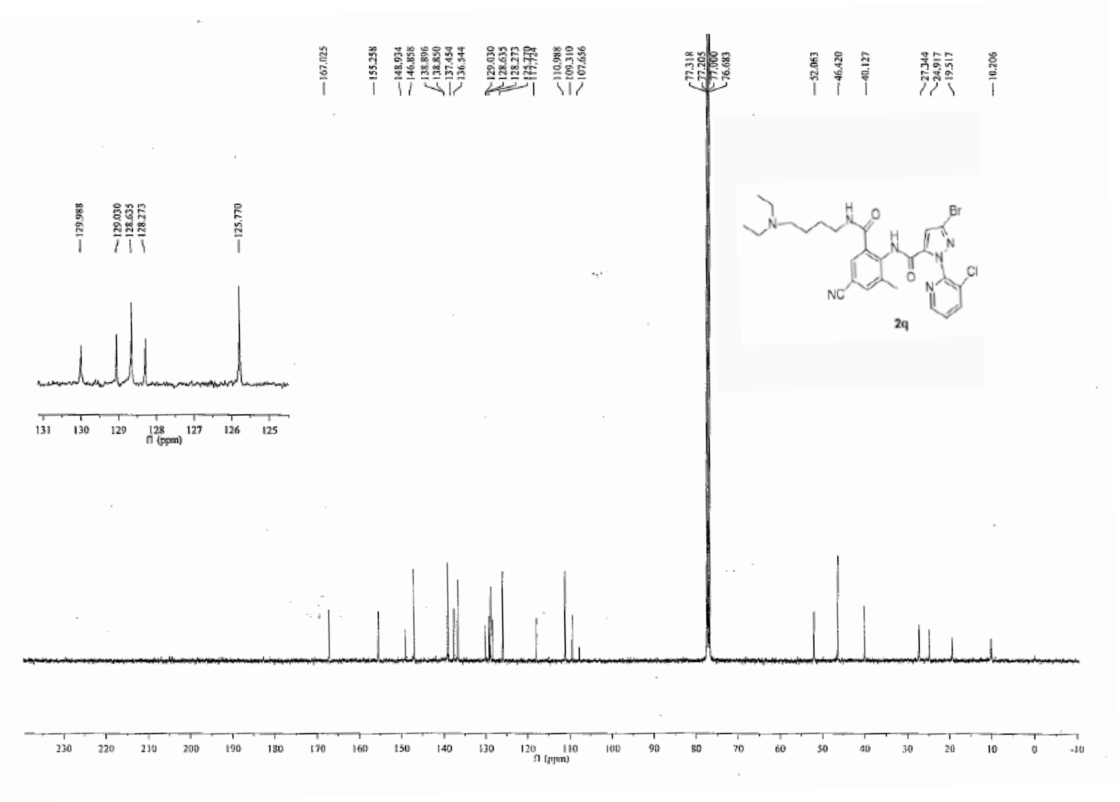

Figure S42. ^{13}C-NMR spectrum of compound **2q**.

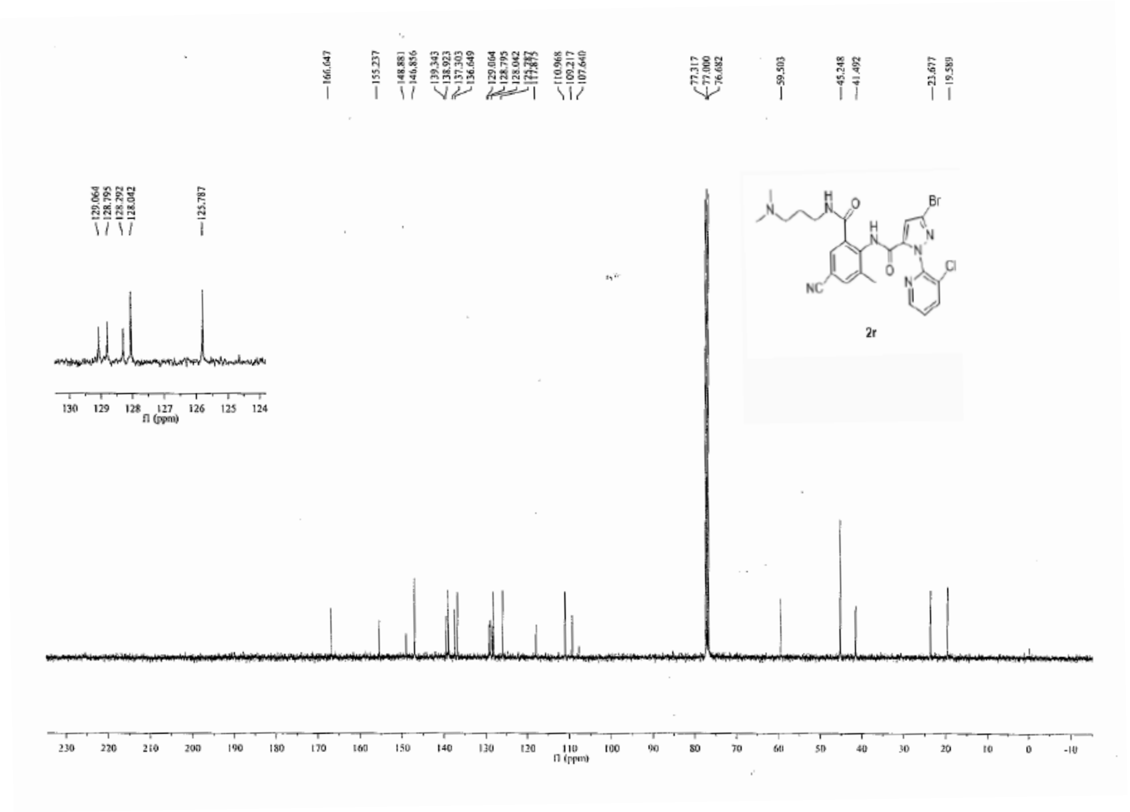

Figure S43. ^{13}C-NMR spectrum of compound **2r**.

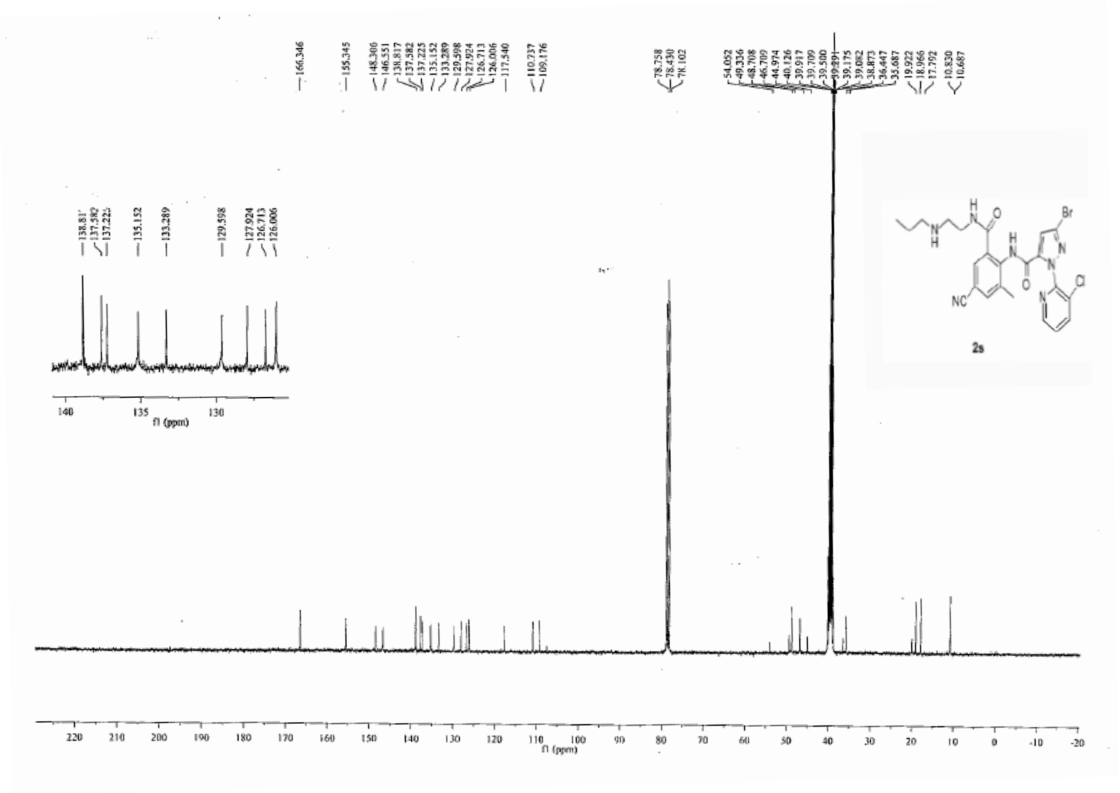

Figure S44. ^{13}C-NMR spectrum of compound **2s**.

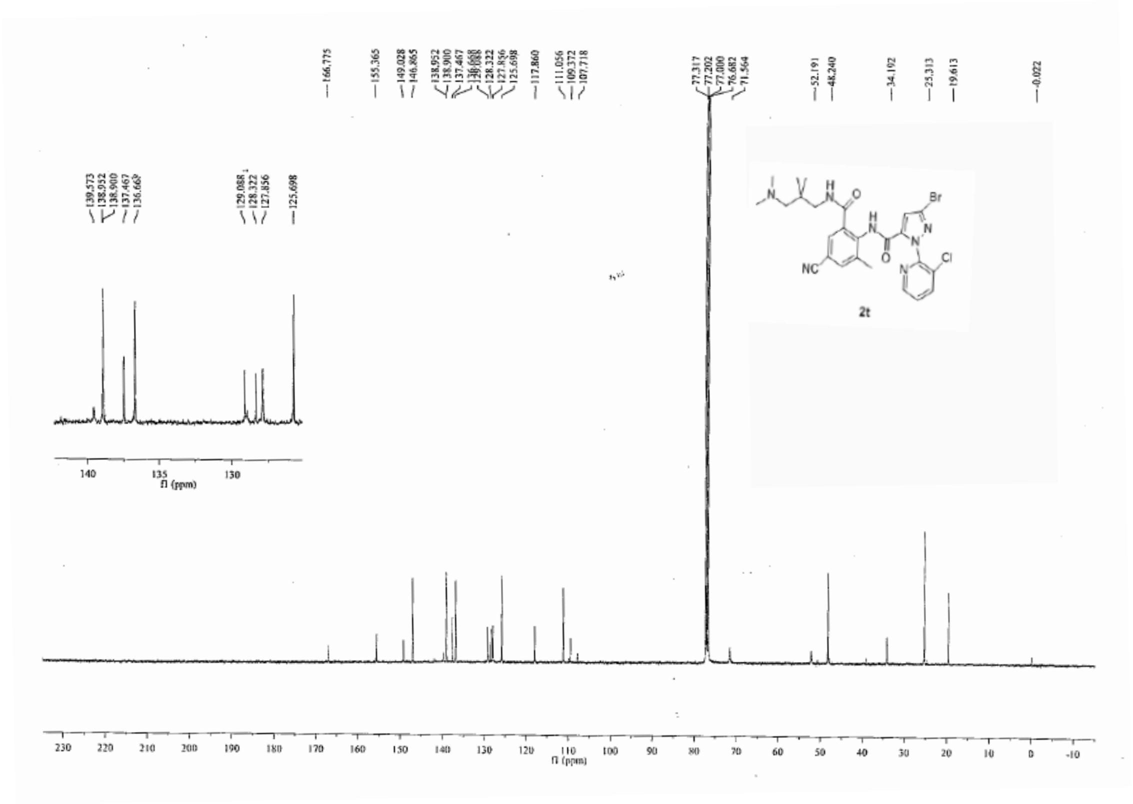

Figure S45. ^{13}C-NMR spectrum of compound **2t**.

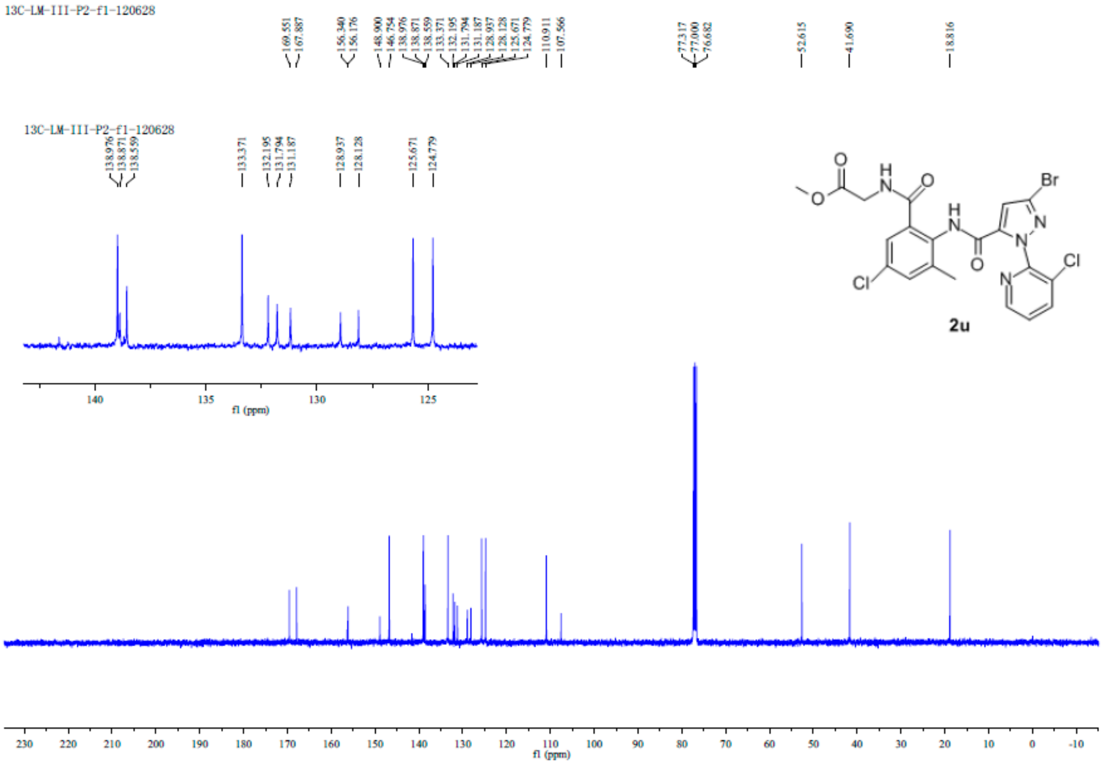

Figure S46. ^{13}C-NMR spectrum of compound **2u**.

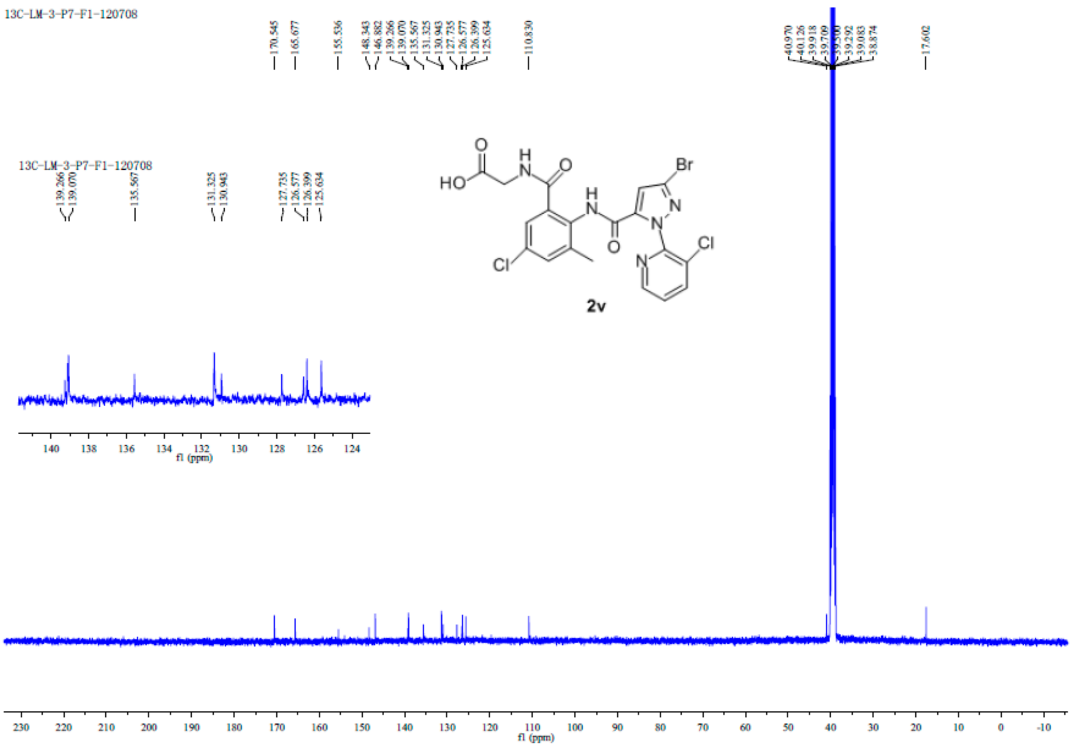

Figure S47. ^{13}C-NMR spectrum of compound **2v**.

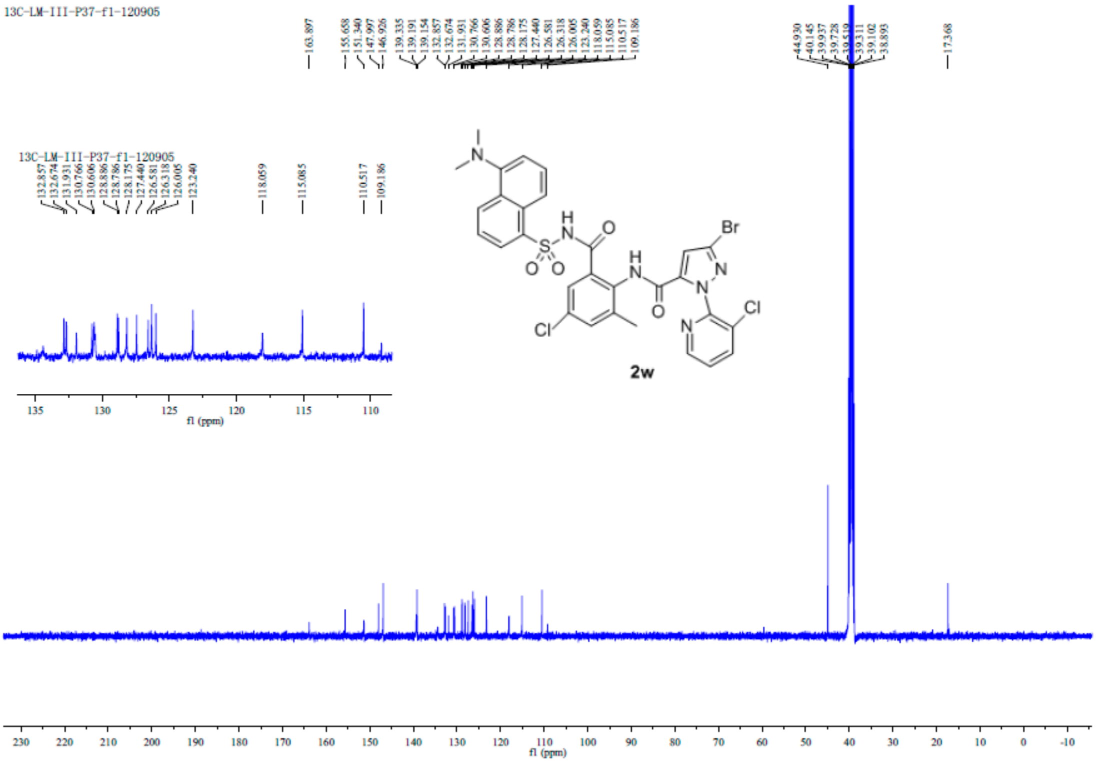

Figure S48. ^{13}C-NMR spectrum of compound **2w**.

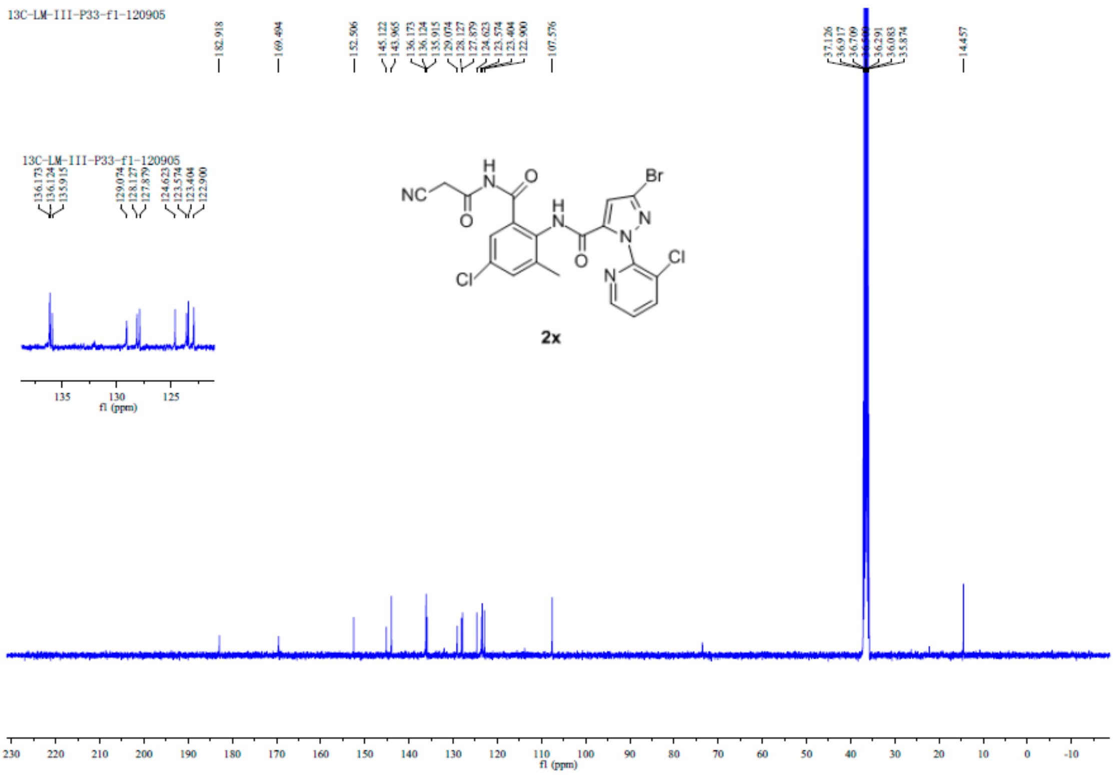

Figure S49. ^{13}C-NMR spectrum of compound **2x**.

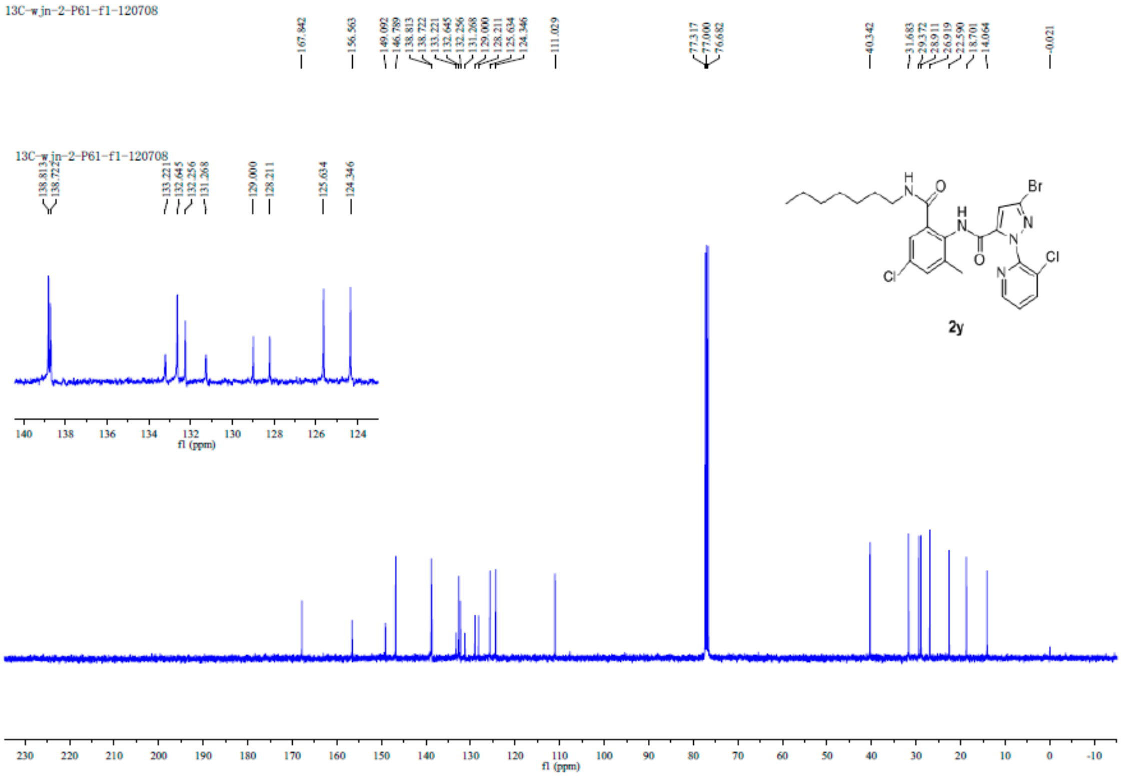

Figure S50. ^{13}C-NMR spectrum of compound **2y**.

3. HR-MS

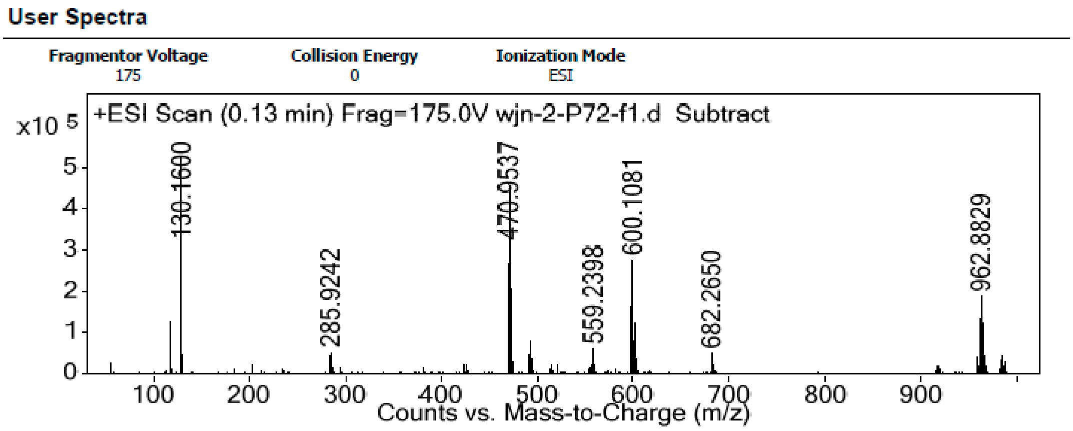

Peak List

m/z	z	Abund
118.1241	1	123911.5
130.16	1	523782
468.9557	1	266677.5
470.9537	1	444060.7
472.9508	1	205094.7
598.1097	1	161127.4
600.1081	1	272552.1
602.1055	1	121480.7
960.8835	1	133528
962.8829	1	185235.3

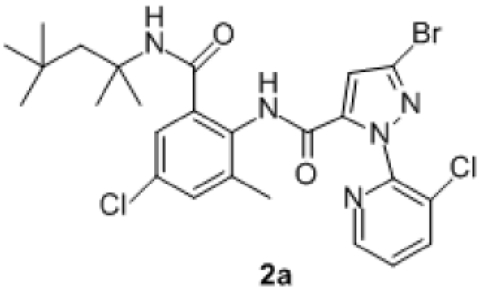

2a

Figure S51. HR-MS spectrum of compound **2a**.

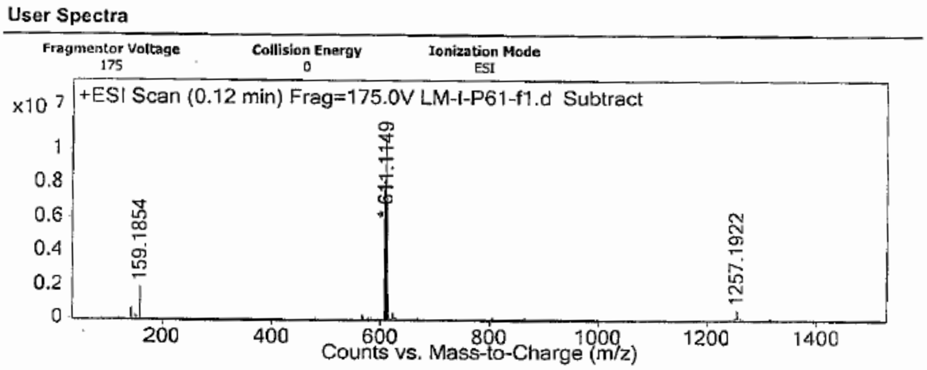

Peak List

m/z	z	Abund
142.1597		678082.6
159.1854		1867007
609.1153	1	8225942.5
610.116	1	3282837.8
611.1149	1	10883353
611.3984		682827.1
612.1142		5100877
613.1104	1	7212181.5
614.1108	1	2559866.5
615.107	1	1506750.1

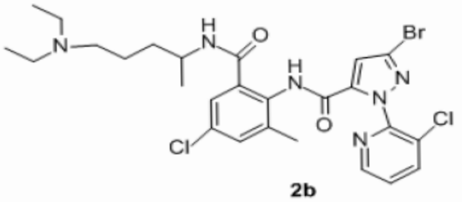

2b

Figure S52. HR-MS spectrum of compound **2b**.

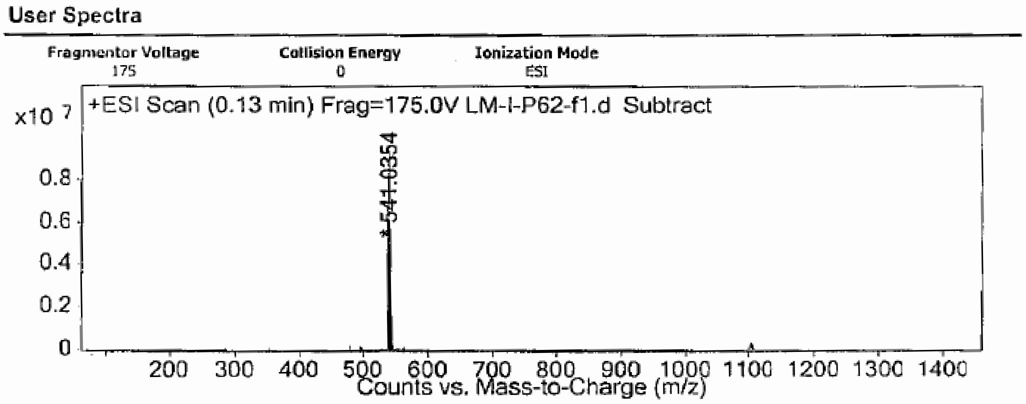

Peak List

m/z	z	Abund
539.0364	1	6839567.5
540.0376	1	2056720.6
541.0354	1	9539774
541.2309		842799.8
541.318		510480.3
542.0355	1	3298000
543.0314	1	5662681
543.2331		491559.4
544.0327	1	1541619
545.0291	1	1010198.8

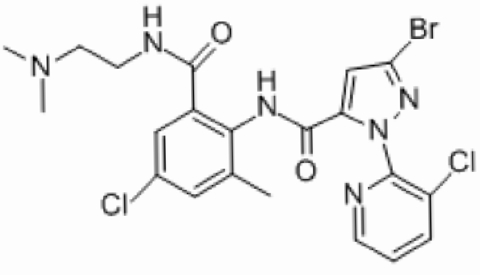

2c

Figure S53. HR-MS spectrum of compound **2c**.

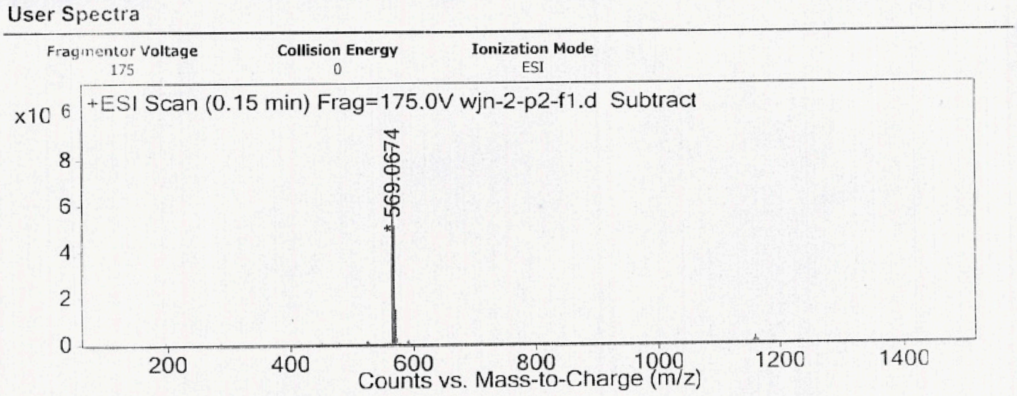

Peak List

m/z	z	Abund
567.0684	1	6086118
567.2721		485601.6
568.0699	1	1932571.3
569.0674	1	8710474
569.2649		751107.5
570.0678	1	3081166
571.0637	1	5068411
571.2718		450103.3
572.0649	1	1456247.1
573.0618	1	887745.3

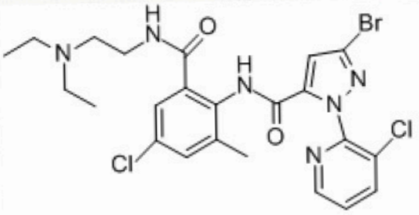

2d

Figure S54. HR-MS spectrum of compound **2d**.

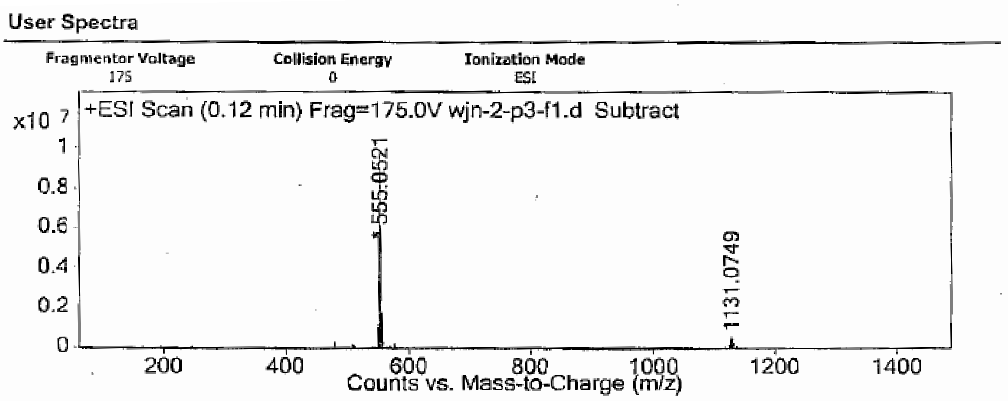

Peak List

m/z	z	Abund
553.053	1	7179975.5
553.2544		508242.2
554.0542	1	2293498
555.0521	1	9874690
555.2473		907458.8
556.0521	1	3656231.8
557.048	1	6022600.5
558.0491	1	1734178
559.0455	1	1089732.3
1131.0749		530363.4

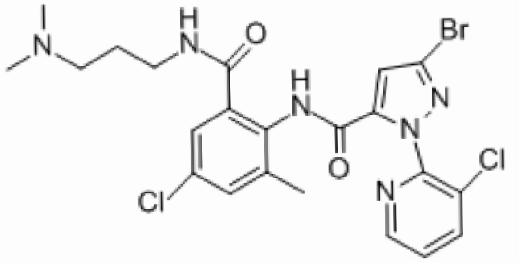

2e

Figure S55. HR-MS spectrum of compound **2e**.

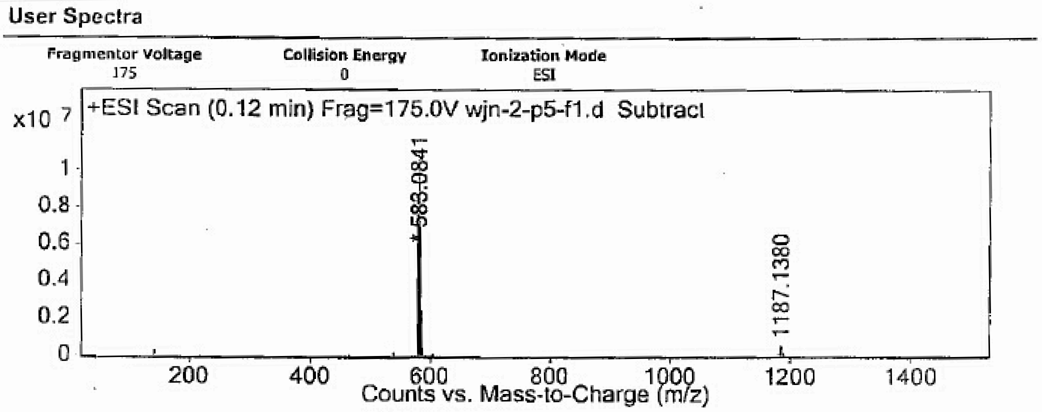

Peak List

m/z	z	Abund
581.0845	1	8488467
581.2841		680058.8
582.0851	1	3105136.8
583.0841	1	11037943
583.362		676582.5
584.0834	1	4881015
585.0796	1	7297667.5
586.08	1	2419787.5
587.0761	1	1465293
1187.138		654161.3

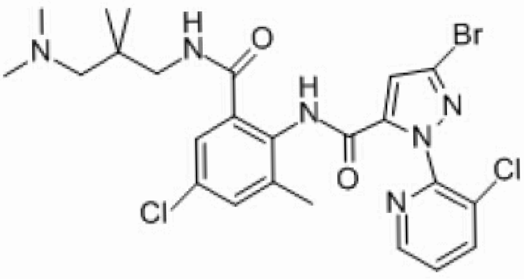

2f

Figure S56. HR-MS spectrum of compound **2f**.

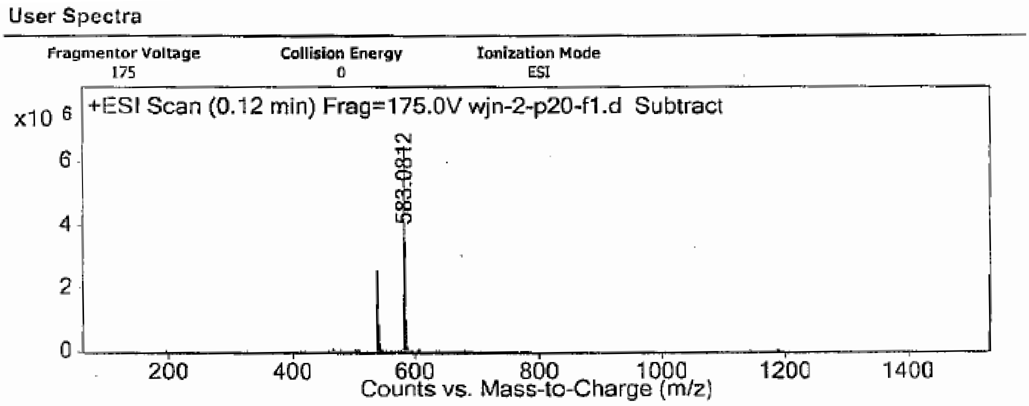

Peak List

m/z	z	Abund
537.1326	1	2552710.5
538.1362	1	720627.1
539.1301	1	2541021.5
541.1289	1	862659.5
581.0826	1	4242290.5
582.0848	1	1267022
583.0812	1	6434385
584.0824	1	2121076.8
585.078	1	3442944.8
586.0807	1	962253.3

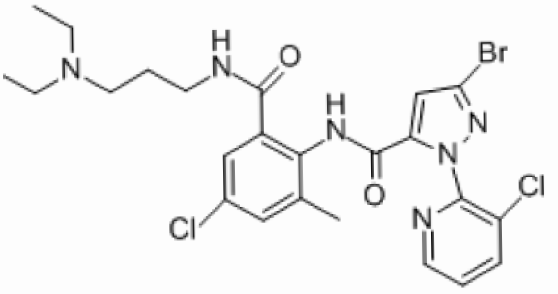

2g

Figure S57. HR-MS spectrum of compound **2g**.

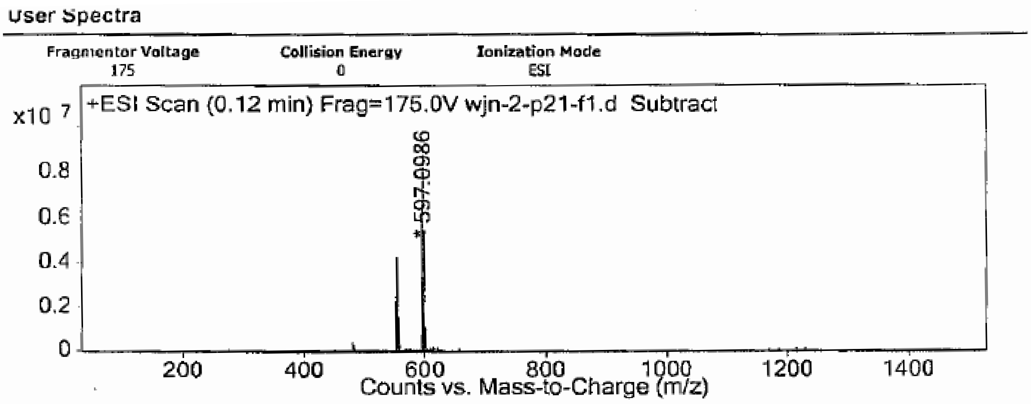

Peak List

m/z	z	Abund
551.1492	1	4181802.8
552.1514	1	1304508.4
553.1466	1	4186059.5
555.1439	1	1518909
595.0996	1	6517437
596.101	1	2253578.3
597.0986	1	9178428
598.0989	1	3565117.5
599.0949	1	5430470.5
600.0959	1	1722469.9

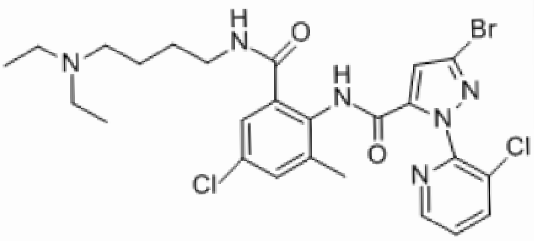

2h

Figure S58. HR-MS spectrum of compound **2h**.

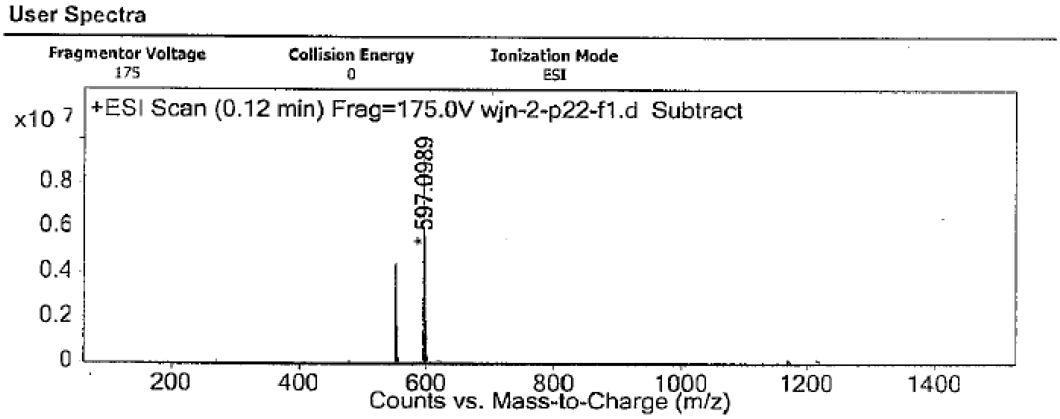

Peak List

m/z	z	Abund
551.1496	1	4350049
552.1518	1	1389648.5
553.147	1	4416446
555.144	1	1634510.1
595.0999	1	6730949.5
596.1012	1	2348590.8
597.0989	1	9423324
598.0991	1	3727104.8
599.0951	1	5624904
600.0963	1	1790023.4

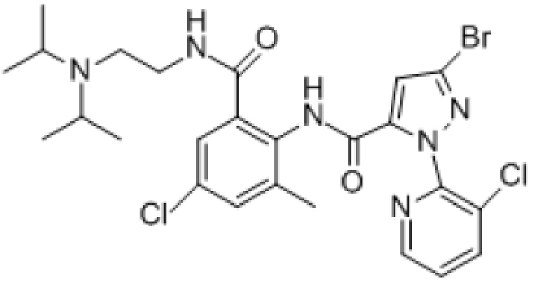

2i

Figure S59. HR-MS spectrum of compound **2i**.

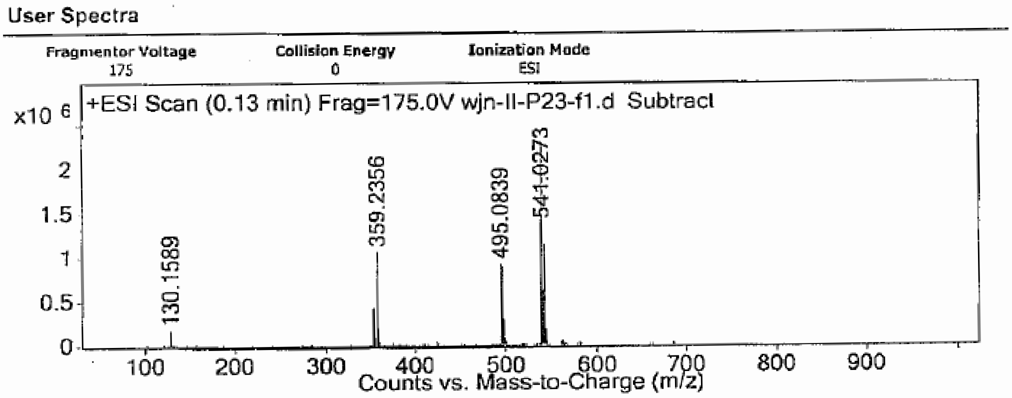

Peak List

m/z	z	Abund
354.2851	1	411753.6
359.2356	1	1065501.4
495.0839	1	923978.9
497.0793	1	890554.1
499.0815	1	294453.5
539.0295	1	1436330.8
540.0394	1	369547
541.0273	1	2306543
542.0368	1	628012.1
543.0255	1	1138796.8

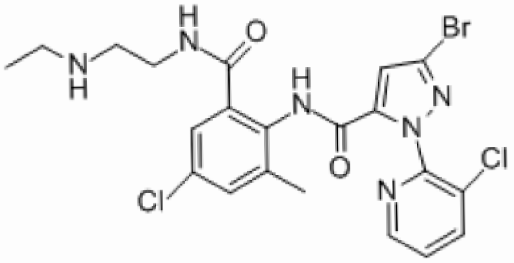

2j

Figure S60. HR-MS spectrum of compound **2j**.

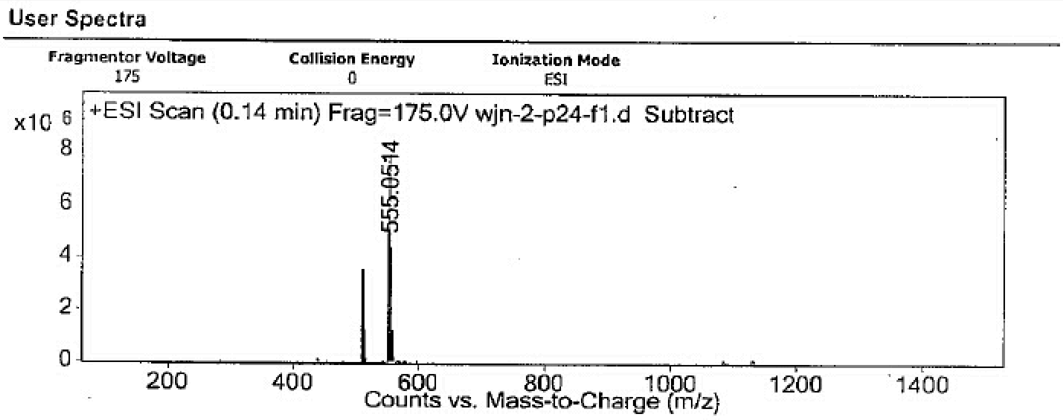

Peak List

m/z	z	Abund
509.1024	1	3522169.5
510.1051	1	951091.7
511.0998	1	3510106.8
513.0974	1	1232857.9
553.0526	1	5343900.5
554.0545	1	1581421.8
555.0514	1	7869936
556.0522	1	2563860.8
557.0477	1	4380764
558.0495	1	1187812.8

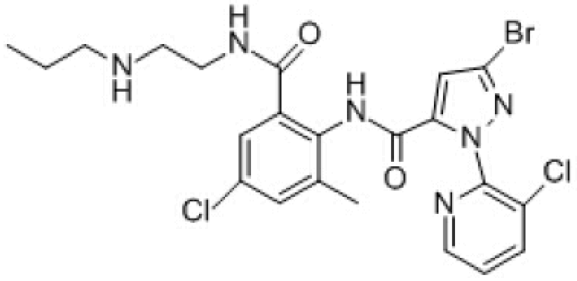

2k

Figure S61. HR-MS spectrum of compound **2k**.

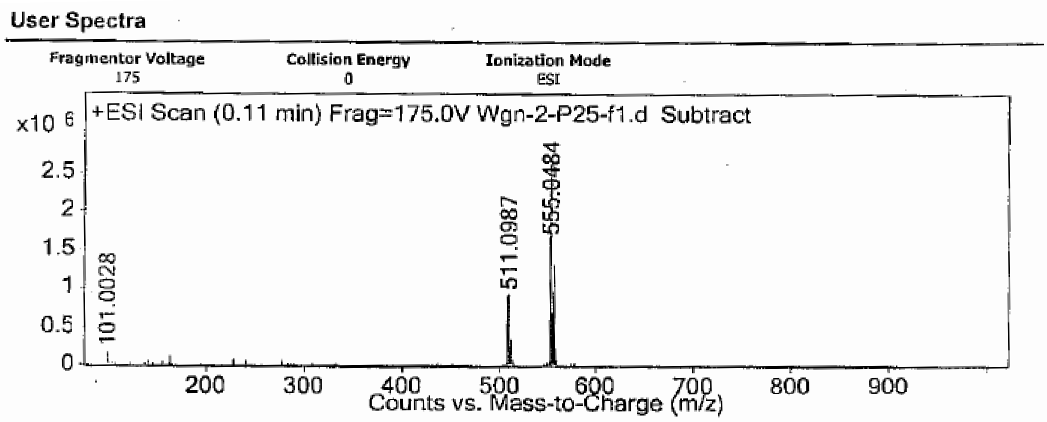

Peak List

m/z	z	Abund
509.1017	1	906035.3
510.1047	1	256031
511.0987	1	914025.5
513.0968	1	327095.2
553.0502	1	1665467.4
554.0547	1	463617.5
555.0484	1	2729755
556.0528	1	695615
557.0462	1	1298244.5
558.0493	1	349623

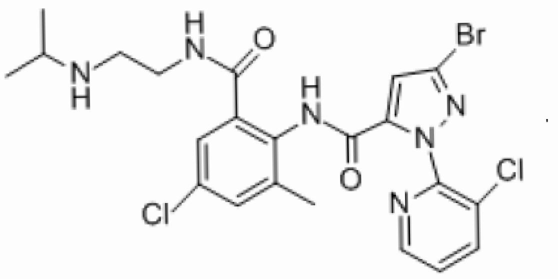

2l

Figure S62. HR-MS spectrum of compound **2l**.

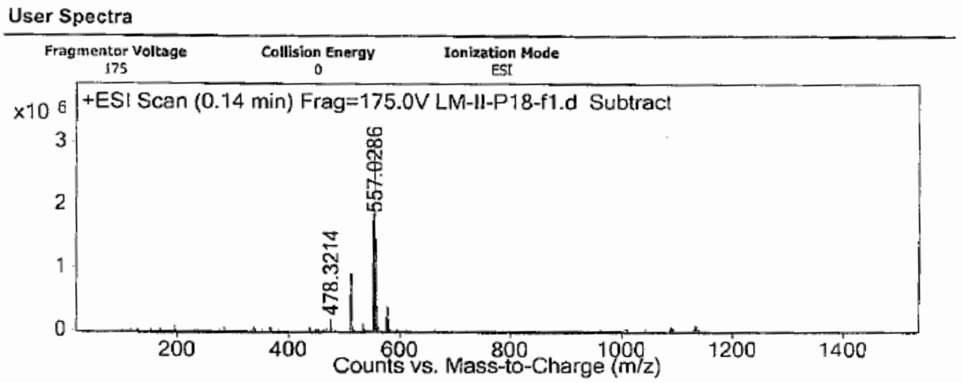

Peak List

m/z	z	Abund
511.0816	1	910876.1
513.0793	1	911321.9
515.0773	1	330544.9
555.0307	1	1842440.6
556.0348	1	506028.7
557.0286	1	3025280.3
558.0316	1	755073.3
559.0262	1	1451913.4
560.0297	1	377341.4
579.0112		375617.2

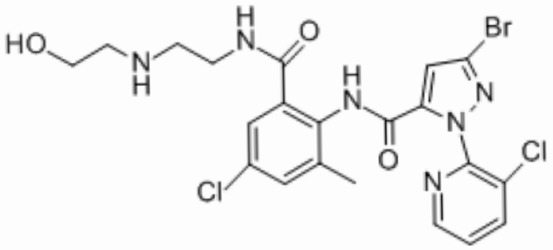

2m

Figure S63. HR-MS spectrum of compound **2m**.

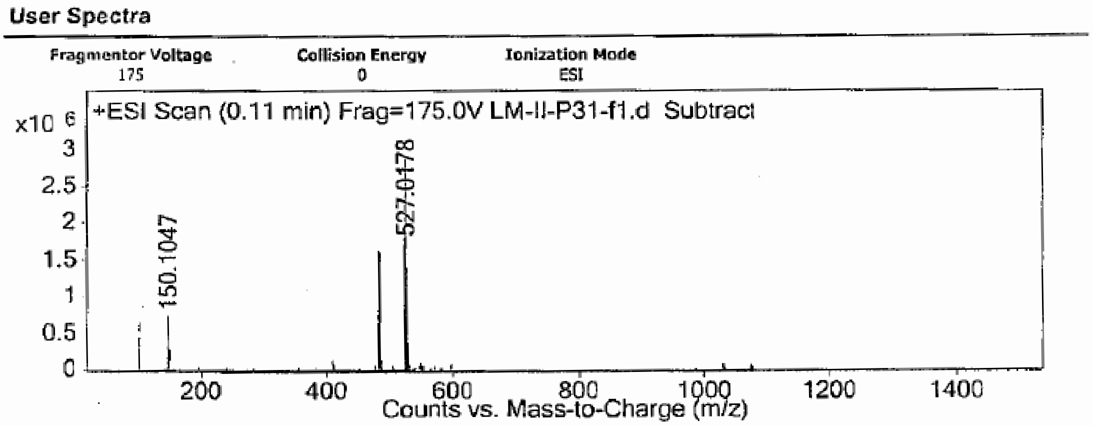

Peak List

m/z	z	Abund
102.1286		658462.4
150.1047		731816.4
481.0703	1	1611672.5
483.0676	1	1606749.8
485.0671	1	505946
525.0199	1	1773646.1
526.0247	1	460097
527.0178	1	2918975.5
528.0215	1	701667.3
529.0155	1	1386773.9

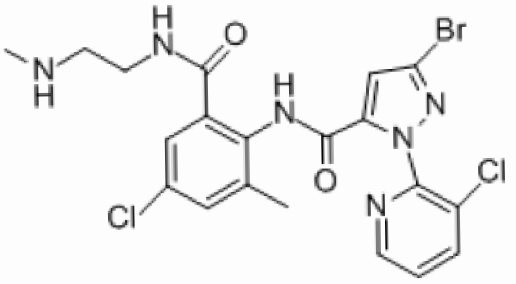

2n

Figure S64. HR-MS spectrum of compound **2n**.

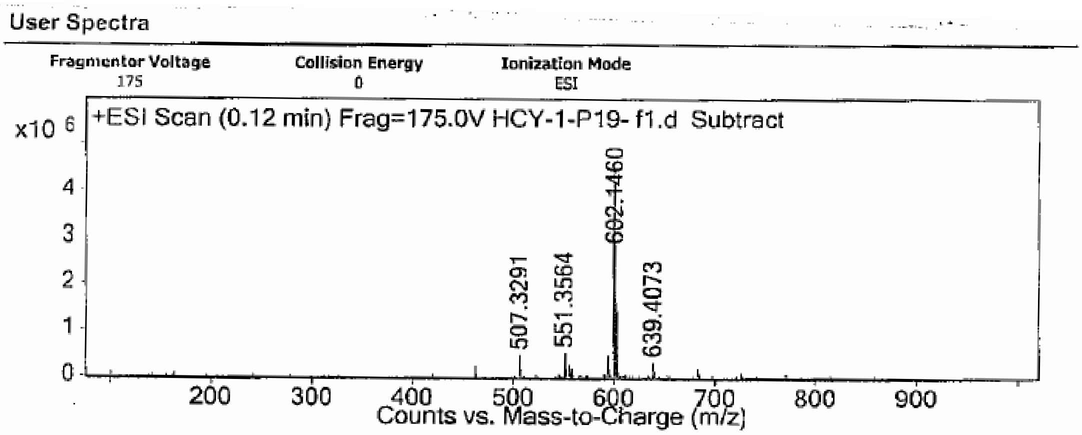

Peak List

m/z	z	Abund
507.3291		452978.6
551.3564		509313.2
595.3817		449043.7
600.1475	1	3538377.3
601.1502	1	1189805.6
602.146	1	4616274
602.3592		356043.1
603.1478	1	1598358.8
604.1437	1	1399279.1
605.147		429666.5

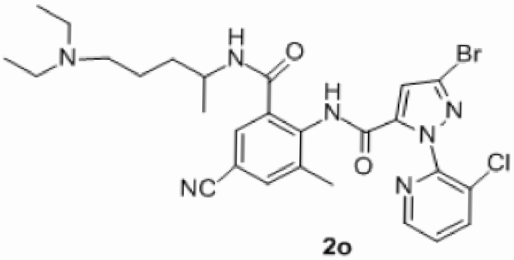

2o

Figure S65. HR-MS spectrum of compound **2o**.

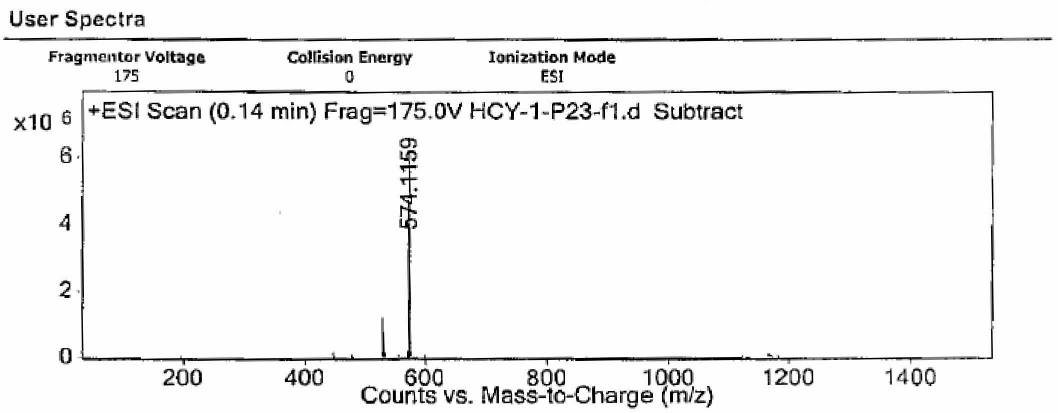

Peak List

m/z	z	Abund
528.1675	1	1183470.8
529.1711	1	384610.2
530.1653	1	788080.2
572.1174	1	4816736.5
573.1194	1	1557544.6
574.1159	1	6151599
574.3197		503864.8
575.1171	1	2054443.9
576.1131	1	1898072.5
577.1177	1	486316.5

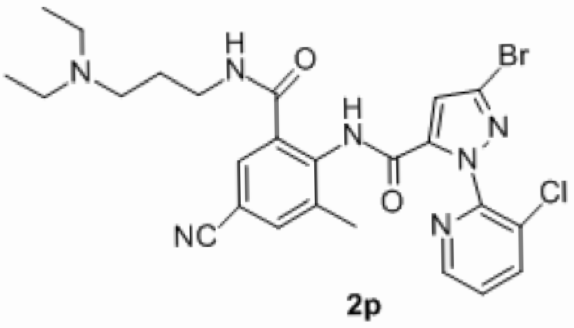

2p

Figure S66. HR-MS spectrum of compound **2p**.

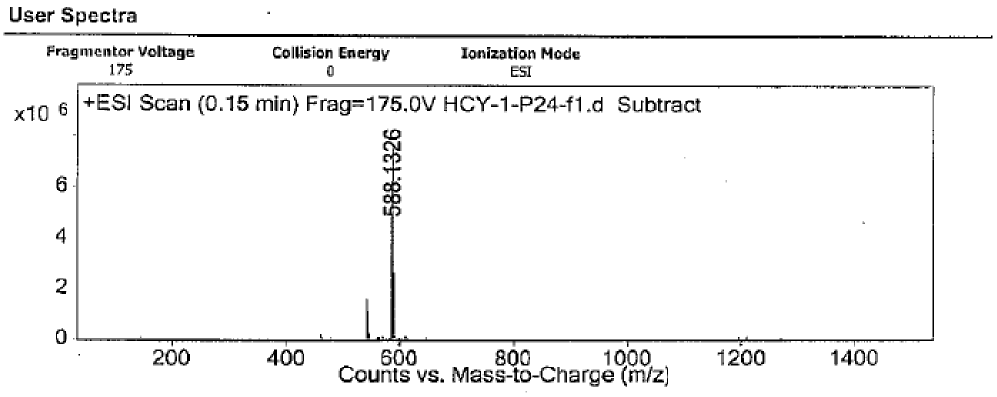

Peak List

m/z	z	Abund
542.183	1	1592069.8
543.188	1	493103.1
544.181	1	1082175.3
586.1339	1	6209576.5
587.1354	1	2182361.3
588.1326	1	7716560.5
588.3347		616641.9
589.1333	1	2874137
590.1291	1	2619945
591.1317	1	734669.8

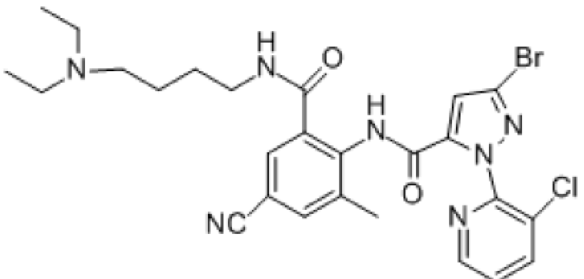

2q

Figure S67. HR-MS spectrum of compound **2q**.

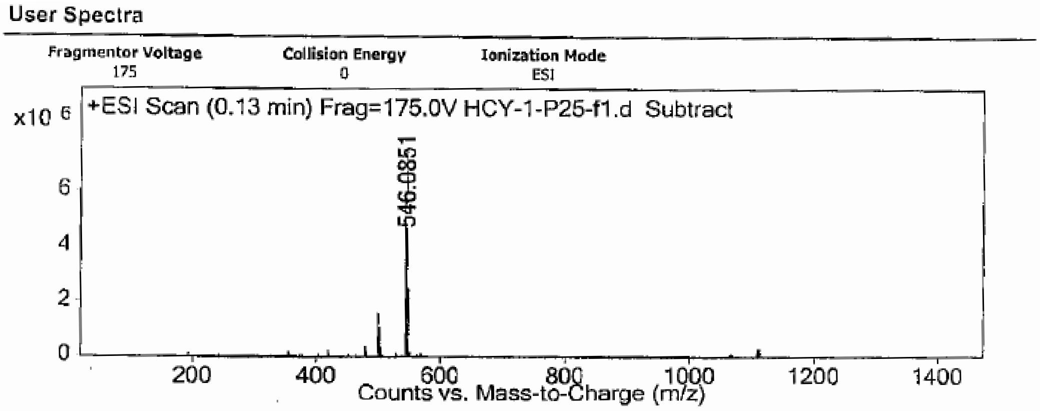

Peak List

m/z	z	Abund
500.1358	1	1529888.5
501.1398	1	457509.8
502.1341	1	1050037.1
544.0866	1	6038569
545.088	1	1890519.5
546.0851	1	7505626.5
546.2849		561615.4
547.086	1	2484396.8
548.0817	1	2462508
549.0856	1	611834.1

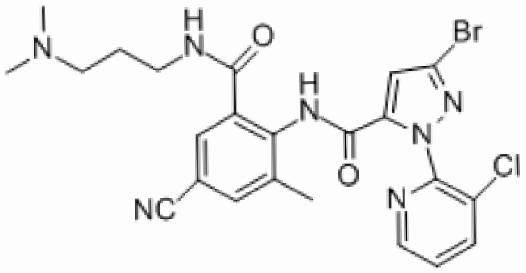

2r

Figure S68. HR-MS spectrum of compound **2r**.

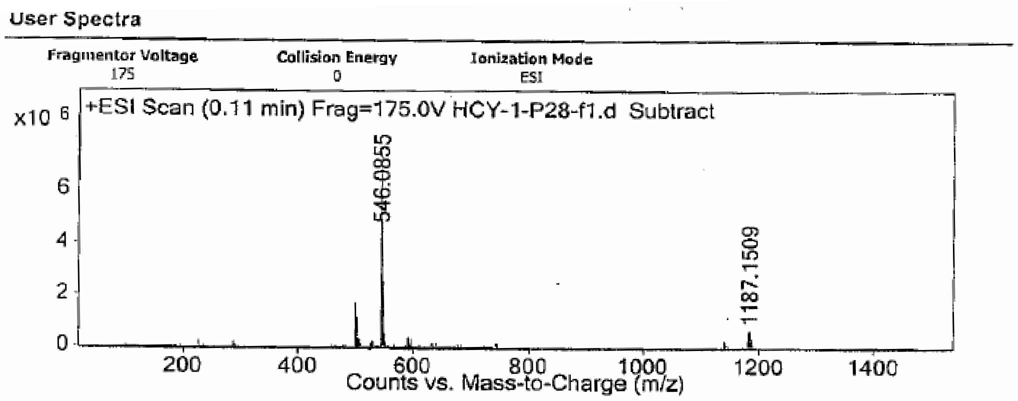

Peak List

m/z	z	Abund
500.1361	1	1699800
502.1342	1	1141259.4
544.0869	1	6157081.5
545.0884	1	1940494.5
546.0855	1	7599645.5
546.3979		698677
547.0863	1	2546678.3
548.0821	1	2487351
549.086	1	628449.8
1187.1509	1	637096.7

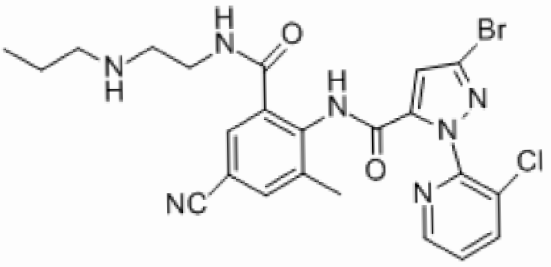

2s

Figure S69. HR-MS spectrum of compound **2s**.

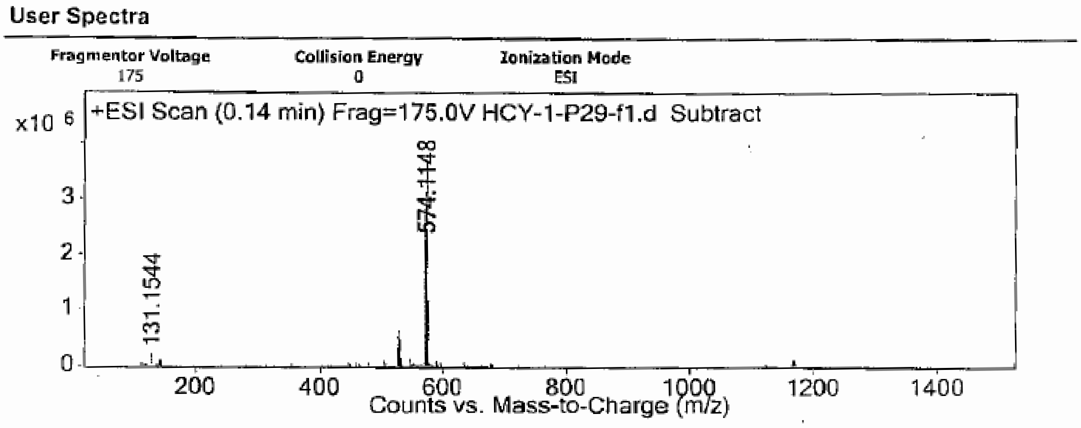

Peak List

m/z	z	Abund
131.1544		212879.2
528.1692	1	648969.2
530.1657	1	489020.4
572.1166	1	2878061
573.12	1	859990.4
574.1148	1	3797398.8
574.3202		280792.6
575.1175	1	1174055.6
576.1129	1	1064154.3
577.1166		310025.8

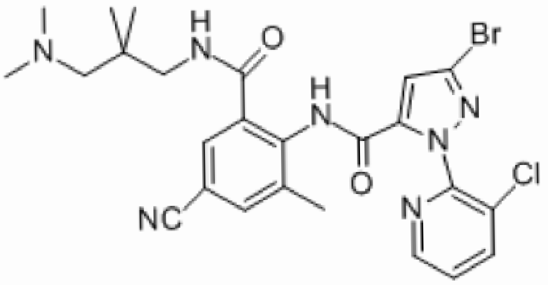

2t

Figure S70. HR-MS spectrum of compound **2t**.

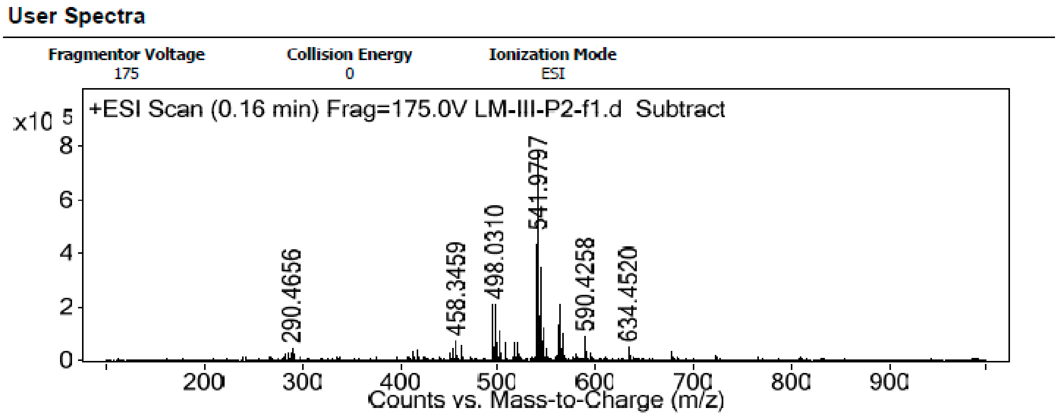

Peak List

m/z	z	Abund
496.0333	1	207511.7
498.031	1	208656.1
502.3725	1	109257.6
539.9837	1	433868.7
541.9797	1	782432.2
542.9837	1	165118.3
543.9792	1	342599.4
546.3989	1	121343.5
561.9643	1	132692.8
563.9633	1	211319.5

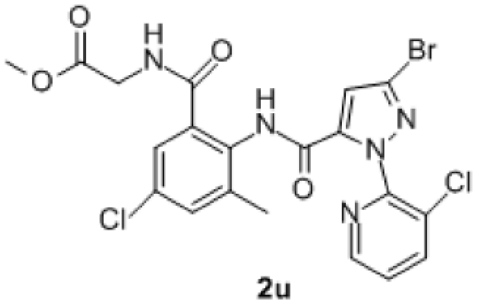

2u

Figure S71. HR-MS spectrum of compound **2u**.

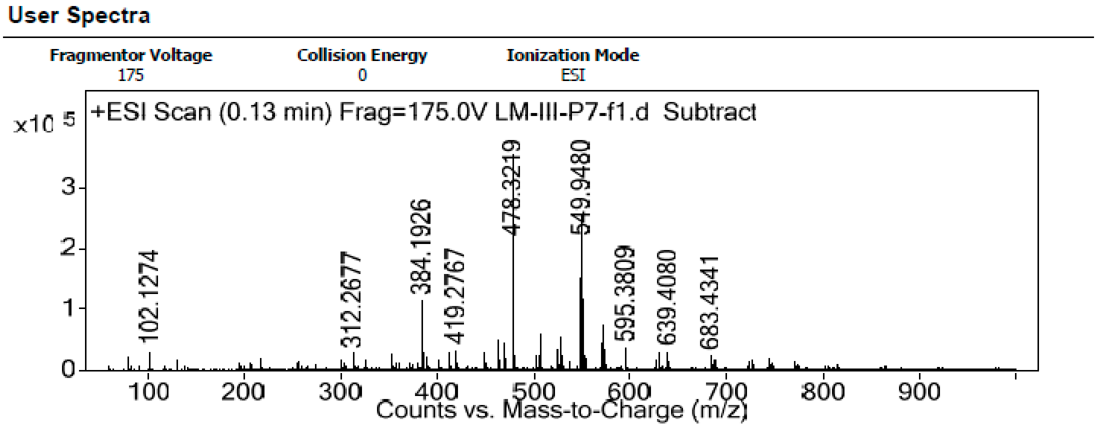

Peak List

m/z	z	Abund
384.1926	1	113644.4
478.3219	1	353356.2
479.3249	1	124734.4
507.3297		57486.6
527.9668		52194.4
547.9505	1	150210.7
549.948	1	256024.5
550.9512	1	54022.7
551.9459	1	117012
571.9301	1	75525.9

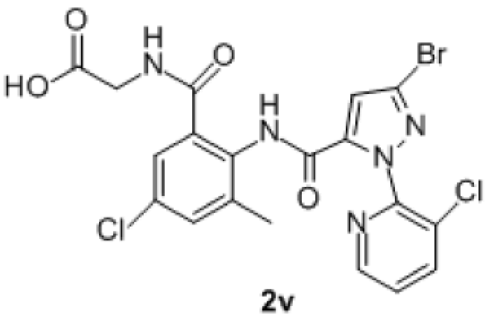

2v

Figure S72. HR-MS spectrum of compound **2v**.

User Spectra

Fragmentor Voltage	Collision Energy	Ionization Mode
175	0	ESI

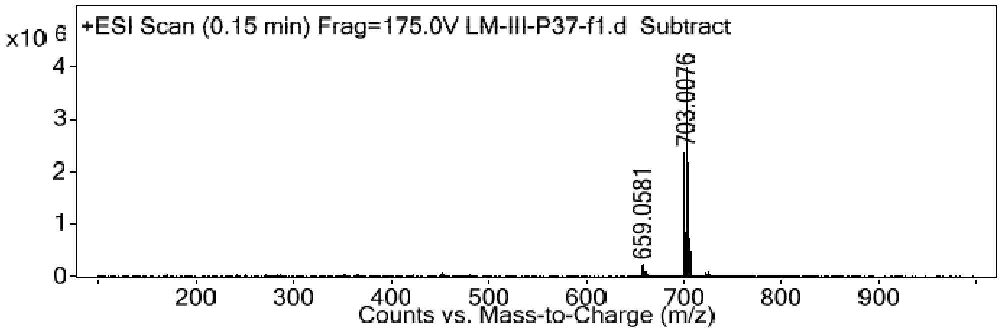

Peak List

m/z	z	Abund
659.0581		211451.5
701.0092	1	2351131.5
702.0128	1	838430.5
703.0076	1	3974236
703.2376		305006.4
704.0095	1	1447541.1
705.0049	1	2159214.5
706.0087	1	707274.3
707.005	1	472477.3

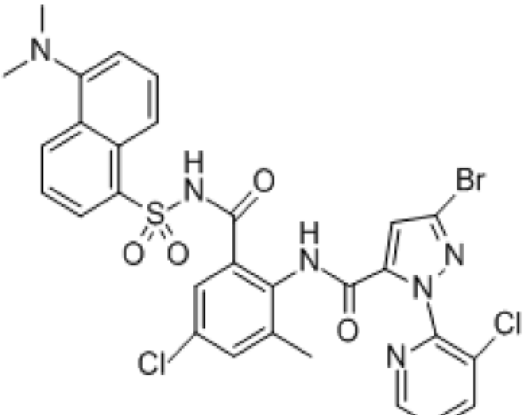

2w

Figure S73. HR-MS spectrum of compound **2w**.

User Spectra

Fragmentor Voltage	Collision Energy	Ionization Mode
175	0	ESI

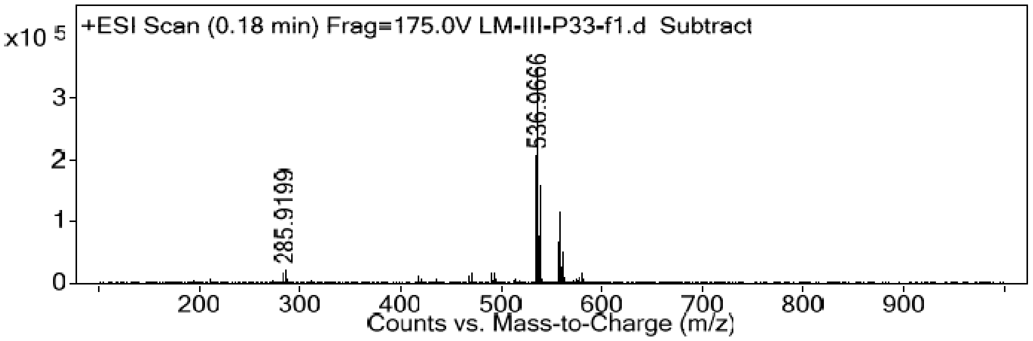

Peak List

m/z	z	Abund
534.9688	1	205748.3
535.9707	1	44536.5
536.9666	1	347538.7
537.9685	1	76448.3
538.9644	1	157774
539.9654	1	34401.2
556.95		66123.5
558.948	1	114424.5
559.9497	1	24350.9
560.9457	1	47238.7

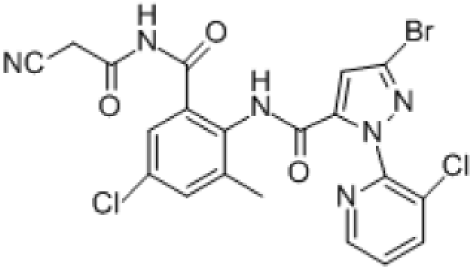

2x

Figure S74. HR-MS spectrum of compound **2x**.

User Spectra

Fragmentor Voltage	Collision Energy	Ionization Mode
175	0	ESI

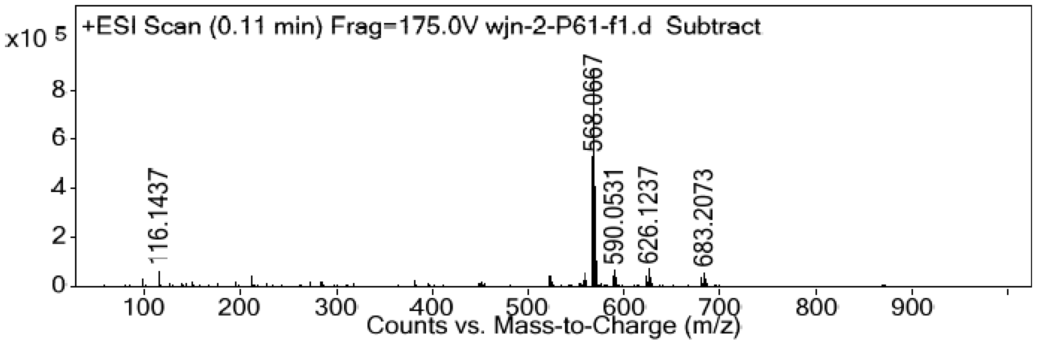

Peak List

m/z	z	Abund
116.1437	1	60162.7
566.074	1	528298.6
567.0763	1	136970
568.0667	1	883447.9
569.0744	1	235335.3
570.07	1	403314.6
571.0719	1	103076.4
572.0684	1	58898
590.0531	1	63897.5
626.1237	1	69808.2

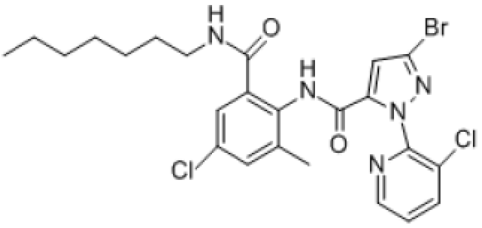

2y

Figure S75. HR-MS spectrum of compound **2y**.
